# Nusinersen effectiveness and safety in pediatric patients with 5q-spinal muscular atrophy: a multi-center disease registry in China

**DOI:** 10.1007/s00415-024-12442-w

**Published:** 2024-07-02

**Authors:** Xiaoli Yao, Jing Peng, Rong Luo, Xiuxia Wang, Xinguo Lu, Liwen Wu, Ruifeng Jin, Jianmin Zhong, Jianmin Liang, Siqi Hong, Lin Yang, Xiaoli Zhang, Shanshan Mao, Jun Hu, Zhe Tao, Dan Sun, Hua Wang, Li Zhang, Yanyan Xia, Ken Chen, Yi Wang

**Affiliations:** 1 Department of Neurology, The First Affiliated Hospital, Sun Yat-Sen University, Guangzhou, China; 2 Department of Pediatrics, Xiangya Hospital Central South University, Changsha, China; 3 Department of Pediatric Neurology, West China Second University Hospital, Sichuan University, Chengdu, China; 4 Department of Pediatric Internal Medicine, The Second Hospital of Hebei Medical University, Shijiazhuang, China; 5 Department of Neurology, Shenzhen Children’s Hospital, Shenzhen, China; 6 Department of Neurology, Hunan Children’s Hospital, Changsha, China; 7 Department of Neurology, Children’s Hospital Affiliated to Shandong University, Jinan, China; 8 Department of Neurology, Jiangxi Provincial Children’s Hospital, Nanchang, China; 9 Department of Pediatric Neurology, The First Hospital of Jilin University, Changchun, China; 10 Department of Neurology, Children’s Hospital of Chongqing Medical University, Chongqing, China; 11 Department of Pediatric Internal Medicine, The Second Affiliated Hospital of Xi’An Jiaotong University, Xi’an, China; 12 Department of Pediatric Neurology, The Third Affiliated Hospital of Zhengzhou University, Zhengzhou, China; 13 Department of Neurology, Children’s Hospital Zhejiang University School of Medicine, Hangzhou, China; 14 Department of Pediatric Internal Medicine, Fujian Medical University Union Hospital, Fuzhou, China; 15 Department of Neurology, Dalian Women and Children’s Medical Group, Dalian, China; 16 Department of Pediatric Neurology, Wuhan Children’s Hospital, Tongji Medical College Huazhong University of Science and Technology, Wuhan, China; 17 Department of Pediatric Neurology, Shengjing Hospital of China Medical University, Shenyang, China; 18 Biogen Biotechnology (Shanghai) Co., Ltd, Shanghai, China; 19 Real World Solutions, IQVIA Solutions Enterprise Management Consulting (Shanghai) Co., Ltd, Shanghai, China; 20 Department of Neurology, Children’s Hospital of Fudan University, Shanghai, China

**Keywords:** Spinal muscular atrophy, Nusinersen, Pediatric, Registry

## Abstract

**Objective:**

To evaluate the effectiveness and safety of nusinersen for the treatment of 5q-spinal muscular atrophy (SMA) among Chinese pediatric patients.

**Methods:**

Using a longitudinal, multi-center registry, both prospective and retrospective data were collected from pediatric patients with 5q-SMA receiving nusinersen treatment across 18 centers in China. All patients fulfilling the eligibility criteria were included consecutively. Motor function outcomes were assessed post-treatment by SMA type. Safety profile was evaluated among patients starting nusinersen treatment post-enrollment. Descriptive analyses were used to report baseline characteristics, effectiveness, and safety results.

**Results:**

As of March 2nd, 2023, 385 patients were included. Most patients demonstrated improvements or stability in motor function across all SMA types. Type II patients demonstrated mean changes [95% confidence interval (CI)] of 4.4 (3.4–5.4) and 4.1 (2.8–5.4) in Hammersmith Functional Motor Scale-Expanded (HFMSE), and 2.4 (1.7–3.1) and 2.3 (1.2–3.4) in Revised Upper Limb Module (RULM) scores at months 6 and 10. Type III patients exhibited mean changes (95% CI) of 3.9 (2.5–5.3) and 4.3 (2.6–6.0) in HFMSE, and 2.1 (1.2–3.0) and 1.5 (0.0–3.0) in RULM scores at months 6 and 10. Of the 132 patients, 62.9% experienced adverse events (AEs). Two patients experienced mild AEs (aseptic meningitis and myalgia) considered to be related to nusinersen by the investigator, with no sequelae.

**Conclusions:**

These data underscore the significance of nusinersen in Chinese pediatric patients with SMA regarding motor function improvement or stability, and support recommendations on nusinersen treatment by Chinese SMA guidelines and continuous coverage of nusinersen by basic medical insurance.

**Supplementary Information:**

The online version contains supplementary material available at 10.1007/s00415-024-12442-w.

## Introduction

Spinal muscular atrophy (SMA) is a neuromuscular disorder characterized by a broad spectrum of clinical presentations, including muscle atrophy and weakness, scoliosis, respiratory insufficiency, and early mortality [1, 2]. Subtypes of SMA are classified based on the age at disease onset and the best motor milestone achieved. SMA types I–III most commonly comprise the pediatric forms. Due to its progressive nature and poor prognosis, SMA, especially the pediatric forms, has substantial mortality and a high healthcare burden for patients [3, 4].

As opposed to symptomatic treatment, disease-modifying therapies (DMT) target the underlying causes of SMA and can help stabilize or potentially improve the current status of the patients. Current DMTs for SMA include nusinersen, onasemnogene abeparvovec, and risdiplam [5–7]. Nusinersen is an antisense oligonucleotide targeting the splicing of exon 7 of the survival motor neuron 2 (*SMN2*) gene, aiming at increasing the production of a full-length SMN protein [5]. In China, nusinersen received approval in 2019 as the first DMT for SMA and was subsequently incorporated into the 2021 National Reimbursement Drug List (NRDL). The effectiveness, mainly characterized by improved motor function, and safety of nusinersen in pediatric patients has been confirmed by randomized controlled trials and SMA disease registry studies from many countries [8–11].

However, the effectiveness and safety of nusinersen in Chinese pediatric patients with SMA has not been well reported. Current real-world studies on the effectiveness and safety of nusinersen among Chinese pediatric patients with SMA are limited by small sample size, short follow-up period, and poor geographic representation [12–14]. The objective of the present analysis is to investigate the effectiveness and safety of nusinersen in a large cohort of Chinese pediatric patients with 5q-SMA based on the registry for pediatric patients.

## Methods

### Study design

**Fig. 1:**
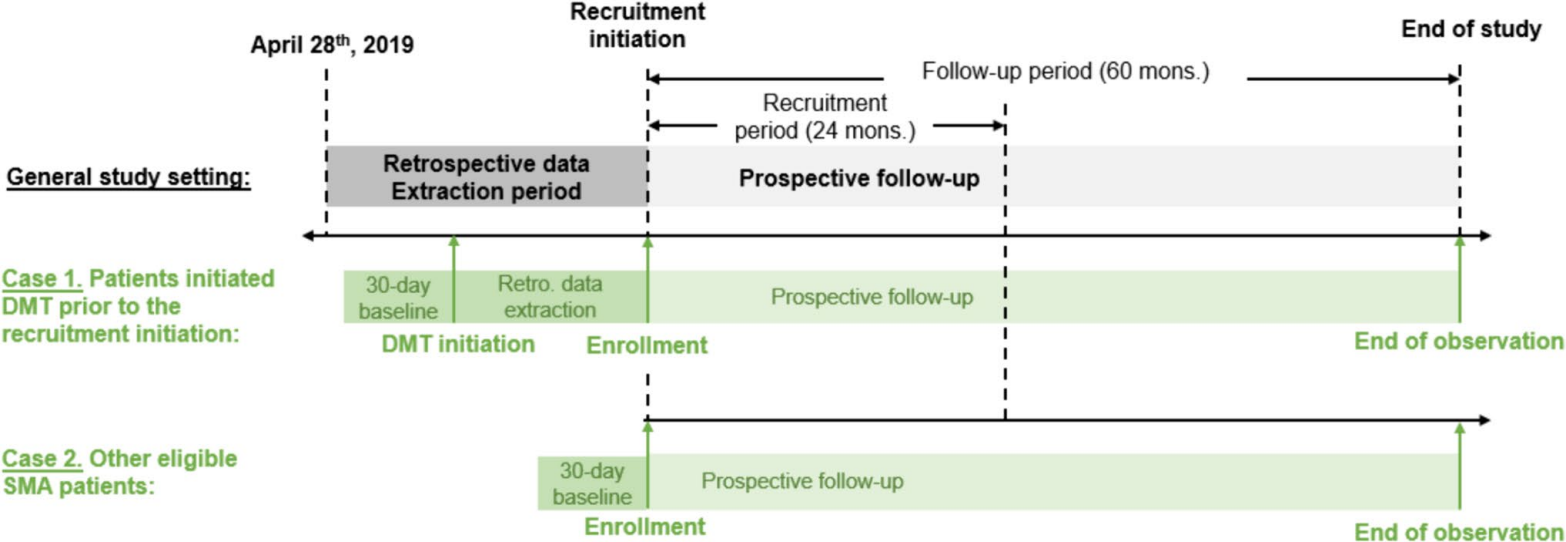
Schematic of study design. *DMT* disease modifying therapy, *SMA* spinal muscular atrophy

A multi-center, longitudinal registry was established in China to both prospectively and retrospectively collect clinical routine data on Chinese pediatric patients with 5q-SMA [15]. The study protocol of the registry is presented in the appendix. Recruitment started in November 2021. The study design is shown in Fig. 1. For patients who initiated DMT prior to recruitment initiation, the registry retrospectively collected available data between DMT initiation and recruitment initiation, and prospectively collected available data since recruitment initiation; otherwise, the registry only prospectively collected available data since recruitment initiation. This analysis used data from the registry on baseline characteristics and effectiveness collected both prospectively and retrospectively, while safety was collected prospectively. The index date was the date of nusinersen initiation. Index date were not earlier than April 28th, 2019, which was the launch date of nusinersen, the first DMT in China. Baseline data were collected within 30 days prior to the index date. Subsequent follow-ups on motor measures were planned on 6, 10, and 14 months post-index date. The study was officially approved by ethical committees of participating sites before starting to collect data in each corresponding site.

### Participants

To be eligible to be included in this registry, patients must meet the following criteria: (1) Ability of the participant and/or his/her legally authorized representative (e.g., parent or legal guardian) to understand the purpose and risks of the study, to provide informed consent, and to authorize the use of confidential health information in accordance with national and local privacy regulations; (2) genetically confirmed 5q-SMA; (3) age < 18 years at registry enrollment. Patients starting nusinersen treatment during the study period regardless of the duration of treatment, dosing frequency, or whether being symptomatic at nusinersen initiation, were included in this analysis.

### Data collection

Data for the registry were obtained within regular, clinically recommended routine visits of SMA patients, depending on their current treatment regimen. All data collected within the registry were entered into the registry-specific e-forms by the responsible investigator or a designated person, as timely as possible. Physicians were asked to include all patients fulfilling the eligibility criteria in a consecutive way, which effectively reduced selection bias. Data obtained within regular patient visits prior to registry inclusion were documented retrospectively. To reduce recall bias on the safety events resulting from retrospective data collection, only patients initiating nusinersen upon or after enrollment by registry were eligible for safety evaluation.

### Outcomes

For motor measures *(Children’s Hospital of Philadelphia Infant Test of Neuromuscular Disorders* (CHOP-INTEND), *Hammersmith Functional Motor Scale-Expanded* (HFMSE), *Hammersmith Infant Neurological Exam–Part 2* (HINE-2), and *Revised Upper Limb Module* (RULM) in this analysis, the score difference between baseline and corresponding visit was calculated for each patient [16–19]. In addition, the response to treatment (categorized as improving, stable, and worsening) at each corresponding visit in terms of the score differences of CHOP-INTEND, HFMSE, and RULM was calculated for each patient. CHOP-INTEND is a 16-item assessment tool developed for evaluating motor function in infants with SMA type I or other neuromuscular disorders. Total possible score ranges from 0 to 64. Improving, stable, and worsening were defined as the score change of ≥ 4, between  – 3 and 3 (inclusive), and ≤  – 4, respectively. HFMSE is a 33-item measure to evaluate the motor function of individuals with later-onset (type II and type III) SMA. Improving, stable, and worsening were defined as the score change of ≥ 3, between -2 and 2 (inclusive), and ≤  – 3, respectively. HINE-2 evaluates motor functions in children with SMA through 8 items: voluntary grasp, ability to kick, head control, rolling, sitting, crawling, standing, and walking. The total HINE-2 score can range from 0 to 26. Thresholds for improving, stable, and worsening were not defined or assessed for HINE-2. RULM is a revised version of the original ULM scale and has been created to measure upper limb function in a wider range of patients. The RULM has 19 items that reflect various functional domains and are graded on a three-point system with a score of 0 (unable), 1 (able, with modification), and 2 (able, no difficulty). Improving, stable, and worsening were defined as the score change of ≥ 3, between -2 and 2 (inclusive), and ≤  – 3, respectively. In this analysis, CHOP-INTEND and HINE-2 were only evaluated for patients with SMA type I, while HFMSE and RULM were evaluated for patients with SMA type II or III.

World Health Organization (WHO) Motor Milestones Assessment is a scale that includes six gross motor milestones (sitting without support, standing with assistance, hands and knees crawling, walking with assistance, standing alone, and walking alone) [20]. In this analysis, the achievement of WHO Motor Milestones Assessment was evaluated among patients under 5 years old. In addition, motor functions (i.e., holding head up without support, rolling onto side, crawling on hands and knees, able to walk 10 m unaided, climbing stairs, useful function of hands, and reaching overhead in a sitting position) were also evaluated in the analysis for all patients with known SMA type.

To evaluate the safety of nusinersen, we reported any adverse events (AEs) occurring during the study period. All AEs were collected and recorded as part of the registry and reported to the study sponsor in a timely manner. An AE was defined as any untoward medical event in a patient or clinical investigation participant administered nusinersen; the event does not necessarily have a causal relationship with nusinersen. Severity of AEs were classified as mild, moderate, or severe by investigators based on patients comfort, performance or functioning, impact on study treatment, and interventions required. An AE was considered “related” to the use of nusinersen if there was a possibility that the event might have been caused by it. Factors that point toward this assessment include, but are not limited to, a positive rechallenge, a reasonable temporal sequence between administration of the product and the event, a known response pattern of the suspected product, improvement following discontinuation or dose reduction, a biologically plausible relationship between the product and the AE, or a lack of an alternative explanation for the AE. The relationship between an AE and nusinersen use was solely at the discretion of investigators.

### Statistical analysis

Descriptive analyses were used to report baseline characteristics, effectiveness, and safety of patients. Continuous variables were summarized by arithmetic mean and standard deviation (SD), or median and interquartile range (IQR) depending on the normality of data. Categorical variables were summarized by the total number of patients and corresponding percentages in each category. For the score difference of motor measures between baseline and corresponding visit, 95% confidence interval (CI) of arithmetic mean was calculated.

In addition, spaghetti plots were produced to describe the trajectory of motor measures (e.g., CHOP-INTEND, HFMSE, HINE-2, and RULM) at baseline and thereafter, regardless of the measuring time post-index date. Patient profile graphs with *x*-axis as age and *y*-axis as each patient were produced to present the achievement of WHO Motor Milestone Assessment and motor function at patient level over time. The incidence rate of AE was calculated from the number of AEs divided by the sum of nusinersen exposure time in patient-year.

All analyses were conducted using SAS® software (SAS Institute Inc., Cary, NC; v9.4 or later). No data imputation was performed for missing data. Pairwise deletion was used to handle the missing data in this analysis.

## Results

### Baseline patient characteristics

As of March 2nd, 2023, a total of 398 patients meeting the eligibility criteria were included in the registry. Of these, 385 patients were treated with nusinersen, and were included in this analysis. The baseline characteristics of patients are shown in Table 1. Out of a total sample of 385 patients, 382 were classified with known SMA types: 41 patients (10.7%) had SMA type I, 214 (56.0%) had type II, and 127 (33.3%) had type III. The analysis comprised 50.9% males (*n* = 196) and 49.1% females (*n* = 189). The median (IQR) age at nusinersen initiation was 42 (7–54) months for patients with type I, 62.5 (32–93) months for type II, and 112 (61–154) months for type III. Eighty percent of patients younger than 60 months had a weight-for-age z-score of greater than -2 at nusinersen initiation. Derived from the WHO Growth Charts, a z-score falling below -2 signifies underweight [21].

**Table 1:** Baseline demographic and clinical characteristics of participants

	Total (*n* = 385)	SMA type I (*n* = 41)	SMA type II (*n* = 214)	SMA type III (*n* = 127)
*Sex*				
Nx	385	41	214	127
Male, *n*(%)	196 (50.9%)	25 (61.0%)	112 (52.3%)	58 (45.7%)
Female, *n*(%)	189 (49.1%)	16 (39.0%)	102 (47.7%)	69 (54.3%)
*Age at nusinersen initiation*				
Nx	385	41	214	127
Median (IQR), month	71 (36–118)	42 (7–54)	62.5 (32–93)	112 (61–154)
< 7 months old, *n*(%)	13 (3.4%)	10 (24.4%)	1 (0.5%)	0 (0%)
≥ 7 months and < 2 years old, *n*(%)	38 (9.9%)	7 (17.1%)	28 (13.1%)	3 (2.4%)
≥ 2 and < 5 years old, *n*(%)	106 (27.5%)	14 (34.2%)	68 (31.8%)	23 (18.1%)
≥ 5 and < 8 years old, *n*(%)	97 (25.2%)	4 (9.8%)	66 (30.8%)	27 (21.3%)
≥ 8 and < 13 years old, *n*(%)	90 (23.4%)	5 (12.2%)	42 (19.6%)	43 (33.9%)
≥ 13 and < 18 years old, *n*(%)	41 (10.7%)	1 (2.4%)	9 (4.2%)	31 (24.4%)
*Height z-score at nusinersen initiation*				
Nx	88	22	55	9
< – 3, *n*(%)	3 (3.4%)	1 (4.5%)	2 (3.6%)	0 (0.0%)
≥ – 3 and ≤ – 2, *n*(%)	13 (14.8%)	4 (18.2%)	8 (14.5%)	1 (11.1%)
> – 2, *n*(%)	72 (81.8%)	17 (77.3%)	45 (81.8%)	8 (88.9%)
*Weight z-score at nusinersen initiation*				
Nx	125	27	80	15
< – 3, *n*(%)	4 (3.2%)	1 (3.7%)	3 (3.8%)	0 (0.0%)
≥ – 3 and ≤ – 2, *n*(%)	21 (16.8%)	4 (14.8%)	17 (21.3%)	0 (0.0%)
> – 2, *n*(%)	100 (80.0%)	22 (81.5%)	60 (75.0%)	15 (100.0%)
*Age at symptom onset*				
Nx	377	40	211	125
Median (IQR), month	12 (7–16)	5 (2.5–6)	10 (7–12)	20 (12–36)
*Age at genetic diagnosis*				
Nx	383	41	214	126
Median (IQR), month	26 (13–69)	7 (5–42)	18 (12–36)	57 (29–119)
*Disease duration before nusinersen initiation*				
Nx	377	40	211	125
Median (IQR), month	56 (23–95)	32 (2.5–55.5)	51 (23–80)	72 (37–113)
≤ 12 weeks, *n*(%)	14 (3.7%)	7 (17.5%)	5 (2.4%)	1 (0.8%)
> 12 weeks–≤ 6 months, *n*(%)	19 (5.0%)	8 (20.0%)	7 (3.3%)	4 (3.0%)
> 6–≤ 24 months, *n*(%)	62 (16.5%)	3 (7.5%)	41 (19.4%)	18 (14.4%)
> 2–≤ 5 years, *n*(%)	101 (26.8%)	12 (30.0%)	64 (30.3%)	25 (20.0%)
> 5–≤ 10 years, *n*(%)	128 (34.0%)	7 (17.5%)	71 (33.7%)	50 (40.0%)
> 10 years, *n*(%)	53 (14.1%)	3 (7.5%)	23 (10.9%)	27 (21.6%)
*Delayed diagnosis*				
Nx	376	40	211	124
Median (IQR), month	10 (3–45)	2 (1–23)	8 (2–24)	20.5 (8–86.5)
≤ 12 weeks, *n*(%)	85 (22.6%)	21 (52.5%)	52 (24.6%)	11 (8.9%)
> 12 weeks–≤ 6 months, *n*(%)	49 (13.0%)	5 (12.5%)	33 (15.6%)	11 (8.9%)
> 6–≤ 24 months, *n*(%)	120 (31.9%)	4 (10.0%)	72 (34.1%)	44 (35.5%)
> 2–≤ 5 years, *n*(%)	39 (10.4%)	4 (10.0%)	22 (10.4%)	13 (10.5%)
> 5–≤ 10 years, *n*(%)	65 (17.3%)	4 (10.0%)	26 (12.3%)	35 (28.2%)
> 10 years, *n*(%)	18 (4.8%)	2 (5.0%)	6 (2.8%)	10 (8.1%)
*SMN2 copy number*				
Nx	292	34	163	93
0 or 1, *n*(%)	0 (0%)	0 (0%)	0 (0%)	0 (0%)
2, *n*(%)	41 (14.0%)	15 (44.1%)	15 (9.2%)	10 (10.8%)
3, *n*(%)	225 (77.1%)	18 (52.9%)	143 (87.7%)	63 (67.7%)
≥ 4, *n*(%)	26 (8.9%)	1 (2.9%)	5 (3.1%)	20 (21.5%)
*SMN1 gene abnormality*				
Nx	381	41	213	125
Deletion, *n*(%)	366 (96.1%)	39 (95.1%)	205 (96.2%)	120 (96.0%)
Mutation, *n*(%)	15 (3.9%)	2 (4.9%)	8 (3.8%)	5 (4.0%)
*Wheelchair usage*				
Nx	264	16	139	108
Yes, *n*(%)	203 (76.9%)	14 (87.5%)	138 (99.3%)	50 (46.3%)
No (able to walk independently), *n*(%)	58 (22.0%)	0 (0%)	0 (0%)	58 (53.7%)
Not Applicable, the subject could not sit independently, *n*(%)	3 (1.1%)	2 (12.5%)	1 (0.7%)	0 (0%)
*CMAP for ulnar nerve left*				
Nx	75	8	33	34
Median (IQR), mV	1.83 (0.87–3.90)	0.51 (0.29–0.94)	1.13 (0.70–2.10)	3.75 (1.83–7.00)
*CMAP for ulnar nerve right*				
Nx	85	7	36	42
Median (IQR), mV	2.60 (1.30–5.20)	0.60 (0.38–2.06)	1.73 (0.91–2.70)	5.17 (3.60–7.56)
*Diagnosis of scoliosis*				
Nx	350	36	192	120
Yes, *n*(%)	171 (48.9%)	18 (50.0%)	93 (48.4%)	60 (50.0%)
No, *n*(%)	179 (51.1%)	18 (50.0%)	99 (51.6%)	60 (50.0%)
*WHO motor milestones reached*^a^				
Sitting without support, *n*/Nx(%)	43/74 (58.1%)	1/14 (7.1%)	29/47 (61.7%)	13/13 (100.0%)
Hands-and-knees crawling, *n*/Nx(%)	10/64 (15.6%)	0/14 (0%)	3/40 (7.5%)	7/10 (70.0%)
Standing with assistance, *n*/Nx(%)	16/66 (24.2%)	0/14 (0%)	5/41 (12.2%)	11/11 (100.0%)
Walking with assistance, *n*/Nx(%)	13/65 (20.0%)	0/13 (0%)	3/41 (7.3%)	10/11 (90.9%)
Standing alone, *n*/Nx(%)	12/67 (17.9%)	0/13 (0%)	0/41 (0%)	12/13 (92.3%)
Walking alone, *n*/Nx(%)	10/67 (14.9%)	0/13 (0%)	0/41 (0%)	10/13 (76.9%)
*CHOP-INTEND score*				
Nx	/	17	/	/
Mean (SD)	/	24.5 (14.1)	/	/
*HFMSE score*				
Nx	/	/	115	91
Mean (SD)	/	/	10.2 (8.9)	36.1 (15.7)
*HINE-2 score*				
Nx	/	17	/	/
Mean (SD)	/	3.1 (2.1)	/	/
*RULM score*				
Nx	/	/	96	77
Mean (SD)	/	/	12.4 (7.8)	27.8 (7.2)

*SMA* Spinal Muscular Atrophy, *IQR* Interquartile Range, *SMN* Survival Motor Neuron, *CMAP* Compound Muscle Action Potential, *WHO* World Health Organization, *SD* Standard Deviation, *CHOP-INTEND* Children’s Hospital of Philadelphia Infant Test of Neuromuscular Disorders, *HFMSE* Hammersmith Functional Motor Scale-Expanded, *HINE-2* Hammersmith Infant Neurological Examination Sect. 2, *RULM* Revised Upper Limb Module

^a^The denominator is the number of patients aged ≤ 5 years with known status of each WHO motor milestone

In terms of age at symptom onset and genetic diagnosis, the median (IQR) age at symptom onset for patients with type I, II, and III was 5 (2.5–6), 10 (7–12), and 20 (12–36) months, respectively, and the median (IQR) age at genetic diagnosis for type I, II, and III was 7 (5–42), 18 (12–36), and 57 (29–119) months, respectively. By calculating the differences between age at symptom onset and nusinersen initiation, the median (IQR) disease duration before nusinersen initiation for patients with type I, II, and III was 32 (2.5–55.5), 51 (23–80), and 72 (37–113) months, respectively. By calculating the differences between age at symptom onset and genetic diagnosis, the median delayed diagnosis time was 2 months in patients with type I, 8 months for type II, and 20.5 months for type III. The percentages of patients with 2, 3, and ≥ 4 *SMN2* copies were 44.1%, 52.9%, and 2.9%, respectively, for type I; 9.2%, 87.7%, and 3.1%, respectively, for type II; and 10.8%, 67.7%, and 21.5%, respectively, for type III. No patients possessed 0 or 1 *SMN2* copies. Of the SMN1 gene abnormality of total patient population, 96.1% presented with SMN1 gene deletion, and the percentages were roughly consistent across all SMA types.

At nusinersen initiation, 87.5% of type I patients reported wheelchair use and almost all type II patients (99.3%) used a wheelchair. Conversely, a lower percentage of type III patients, 46.3%, reported wheelchair usage at nusinersen initiation. The median (IQR) compound muscle action potential (CMAP) amplitude for type I, II, and III patients was 0.51 (0.29–0.94), 1.13 (0.70–2.10), and 3.75 (1.83–7.00) mV for the left ulnar nerve, and 0.60 (0.38–2.06), 1.73 (0.91–2.70), and 5.17 (3.60–7.56) mV for the right ulnar nerve, respectively. About half of the patients were diagnosed with scoliosis across all SMA types. In relation to motor measures, the mean (SD) CHOP-INTEND and HINE-2 scores at nusinersen initiation for patients with SMA type I were 24.5 (14.1) and 3.1 (2.1), respectively. As for HFMSE and RULM, patients with SMA type II had mean (SD) scores of 10.2 (8.9) and 12.4 (7.8), while patients with SMA type III exhibited mean (SD) scores of 36.1 (15.7) and 27.8 (7.2) at the commencement of nusinersen therapy. Regarding WHO motor milestones, the percentages of type III patients achieving the milestone were greater than 70% for all 6 milestones; for type II patients, besides sitting without support (61.7%) and standing with assistance (12.2%), the attainment of the remaining milestones were all below 10%; the sole milestone reached by type I patients was sitting without support (7.1%).

### Motor measures

**Fig. 2:**
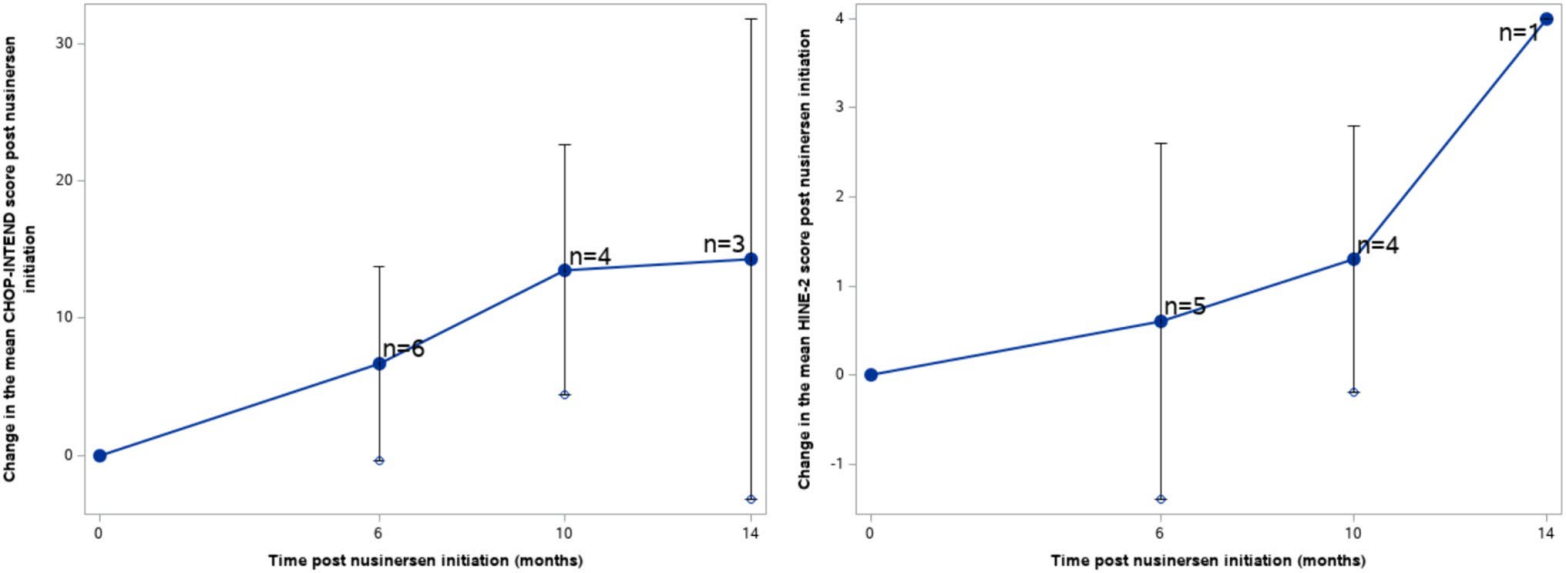
Changes in CHOP-INTEND and HINE-2 scores in patients with SMA type 1. *SMA* Spinal Muscular Atrophy, *CHOP-INTEND* Children’s Hospital of Philadelphia Infant Test of Neuromuscular Disorders, *HINE-2* Hammersmith Infant Neurological Examination Sect. 2

**Fig. 3:**
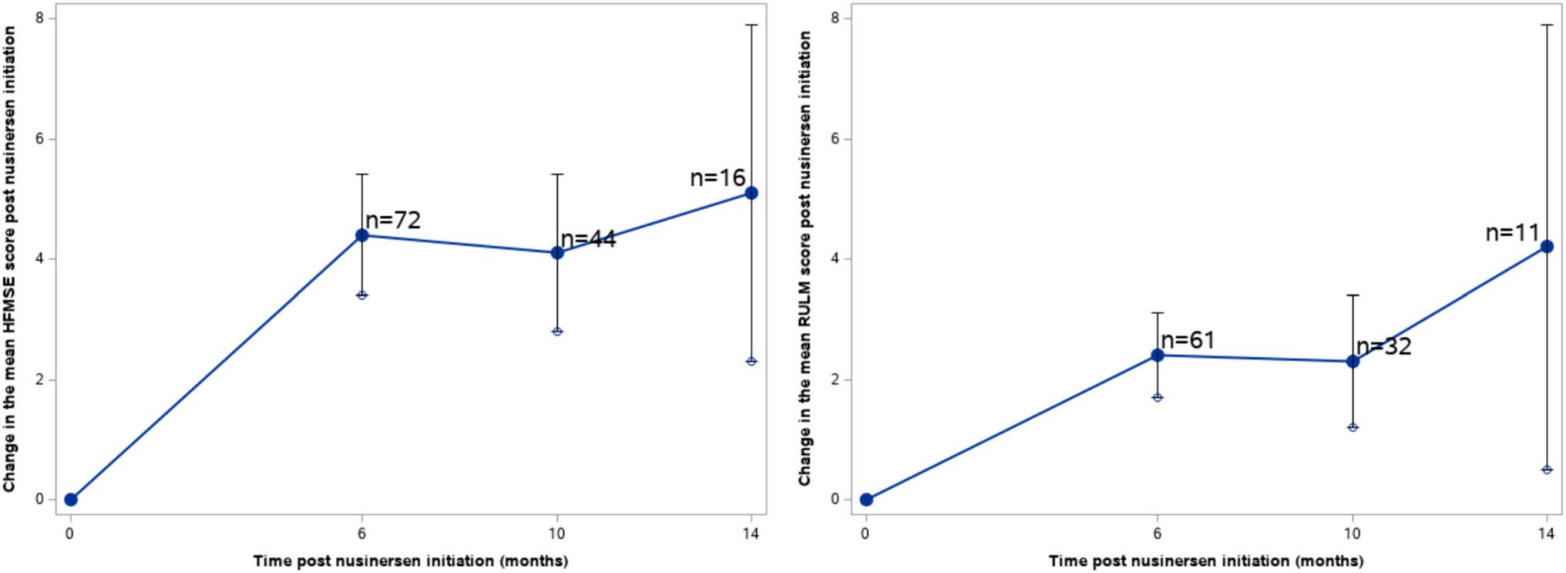
Changes in HFMSE and RULM scores in patients with SMA type 2. *SMA* Spinal Muscular Atrophy, *HFMSE* Hammersmith Functional Motor Scale-Expanded, *RULM* Revised Upper Limb Module

**Fig. 4:**
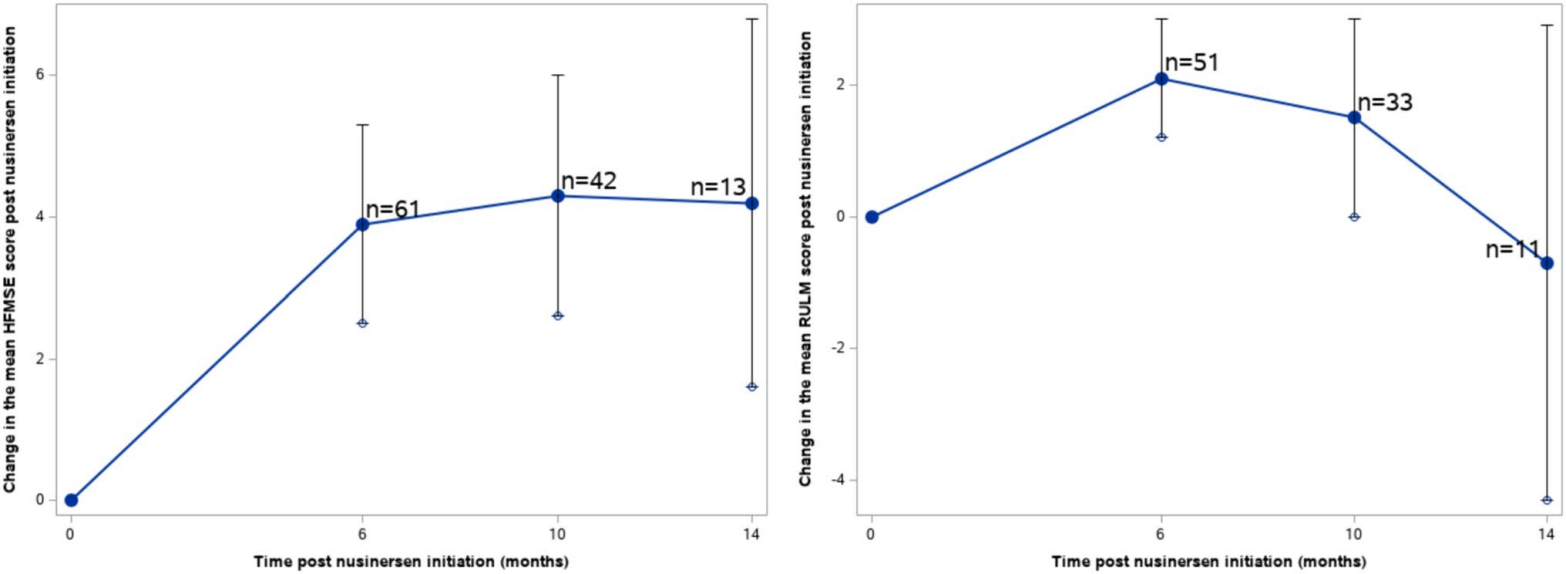
Changes in HFMSE and RULM scores in patients with SMA type 3. *SMA* Spinal Muscular Atrophy, *HFMSE* Hammersmith Functional Motor Scale-Expanded, *RULM* Revised Upper Limb Module

The score changes from baseline over 6, 10, and 14 months after nusinersen initiation in the CHOP-INTEND and HINE-2 scales across type I, and HFMSE and RULM scales across type II and type III patients are presented in Table 2. The majority of motor measure scores increased from the baseline to each subsequent time point. Pertaining to patients with SMA type I, the mean (SD) changes in motor measures from baseline at months 6, 10, and 14 was 6.7 (8.9), 13.5 (9.3), and 14.3 (15.5) for CHOP-INTEND, respectively. The mean (SD) changes in HINE-2 score at months 6, 10, and 14 was 0.6 (2.3), 1.3 (1.5), and 4.0 ( – ), respectively. For patients with SMA type II, the mean (SD) changes from baseline at months 6, 10, and 14 were 4.4 (4.4), 4.1 (4.5), and 5.1 (5.7) for HFMSE, and 2.4 (3.0), 2.3 (3.1), and 4.2 (6.2) for RULM, respectively. Type III patients exhibited mean (SD) changes of 3.9 (5.6), 4.3 (5.6), and 4.2 (4.7) in HFMSE score, and means (SD) changes of 2.1 (3.4), 1.5 (4.4), and  – 0.7 (6.0) in RULM score at months 6, 10, and 14, respectively. These changes over time were also visualized in Figs. 2, 3, 4. The results of the subgroup analysis, which examined the impact of *SMN2* copy number and age at drug initiation on motor measures, are presented in the appendix.

**Table 2:** Changes in motor measures at Month 6, 10, and 14 post-nusinersen initiation by SMA type

	SMA type I (*n* = 41)			SMA type II (*n* = 214)			SMA type III (*n* = 127)		
	M6	M10	M14	M6	M10	M14	M6	M10	M14
*Motor measure*	*CHOP-INTEND*			*HFMSE*					
Nx^a^	6	4	3	72	44	16	61	42	13
Mean (SD)	6.7 (8.9)	13.5 (9.3)	14.3 (15.5)	4.4 (4.4)	4.1 (4.5)	5.1 (5.7)	3.9 (5.6)	4.3 (5.6)	4.2 (4.7)
95% CI	– 0.4, 13.8	4.4, 22.6	– 3.2, 31.8	3.4, 5.4	2.8, 5.4	2.3, 7.9	2.5, 5.3	2.6, 6.0	1.6, 6.8
Improving^b^, *n*(%)	2 (33.3%)	4 (100%)	2 (66.7%)	45 (62.5%)	24 (54.6%)	10 (62.5%)	37 (60.7%)	24 (57.1%)	8 (61.5%)
Stable^c^, *n*(%)	4 (66.7%)	0 (0%)	1 (33.3%)	26 (36.1%)	20 (45.5%)	5 (31.3%)	19 (31.2%)	15 (35.7%)	5 (38.5%)
Worsening^d^, *n*(%)	0 (0%)	0 (0%)	0 (0%)	1 (1.4%)	0 (0%)	1 (6.3%)	5 (8.2%)	3 (7.1%)	0 (0%)
*Motor measure*	*HINE-2*			*RULM*					
Nx^a^	5	4	1	61	32	11	51	33	11
Mean (SD)	0.6 (2.3)	1.3 (1.5)	4.0 (– )	2.4 (3.0)	2.3 (3.1)	4.2 (6.2)	2.1 (3.4)	1.5 (4.4)	– 0.7 (6.0)
95% CI	– 1.4, 2.6	– 0.2, 2.8	–	1.7, 3.1	1.2, 3.4	0.5, 7.9	1.2, 3.0	0.0, 3.0	– 4.3, 2.9
Improving^b^, *n*(%)	/	/	/	32 (52.5%)	15 (46.9%)	6 (54.6%)	17 (33.3%)	11 (33.3%)	5 (45.5%)
Stable^c^, *n*(%)	/	/	/	26 (42.6%)	16 (50.0%)	5 (45.5%)	33 (64.7%)	20 (60.6%)	3 (27.3%)
Worsening^d^, *n*(%)	/	/	/	3 (4.9%)	1 (3.1%)	0 (0%)	1 (2.0%)	2 (6.1%)	3 (27.3%)

*SMA* spinal muscular atrophy, *SD* standard deviation, *CHOP-INTEND* Children’s Hospital of Philadelphia infant test of neuromuscular disorders, *HFMSE* hammersmith functional motor scale-expanded, *HINE-2* Hammersmith Infant neurological examination Sect. 2, *RULM* revised upper limb module, *CI* confidence interval

^a^Nx denotes the number of patients who underwent the respective motor measure assessments at both nusinersen initiation and the specified timepoint

^b^Improving is defined by a score change of ≥ 4 for CHOP-INTEND, ≥ 3 for HFMSE, and ≥ 3 for RULM, when comparing the baseline to the corresponding visit

^c^Stable is defined by a score change between -3 to 3 (inclusive) for CHOP-INTEND,  – 2 to 2 (inclusive) for HFMSE, and  – 2 to 2 (inclusive) for RULM, when comparing the baseline to the corresponding visit

^d^Worsening is defined by a score change of ≤  – 4 for CHOP-INTEND, ≤   – 3 for HFMSE, and ≤  – 3 for RULM, when comparing the baseline to the corresponding visit

The patients’ response to treatment categorized by improvements, stability, or worsening of the CHOP-INTEND, HFMSE, and RULM scales is shown in Table 2. The percentages of patients whose motor function improved or remained stable were mostly above 90% across SMA type, motor measures, and time points. Pertaining to SMA type I patients, improvement rate as indicated by CHOP-INTEND scores was 33.3%, 100.0%, and 66.7% at months 6, 10, and 14, respectively. Among patients with SMA type II, the percentages of patients showing improvement regarding HFMSE were 62.5% at month 6, 54.6% at month 10, and 62.5% at month 14. As for RULM scores, the percentages of patients indicating improvement were 52.5%, 46.9%, and 54.6% at the same time point. Similar patterns were exhibited among type III patients. HFMSE improvement percentage was 60.7%, 57.1%, and 61.5% at months 6, 10, and 14, respectively. Assessed through the lens of RULM, the percentages of patients demonstrating improvement were 33.3% at both months 6 and 10, escalating to 45.5% at month 14. Subgroup analyses on the response to treatment were also conducted, the results of which are provided in the appendix.

**Fig. 5:**
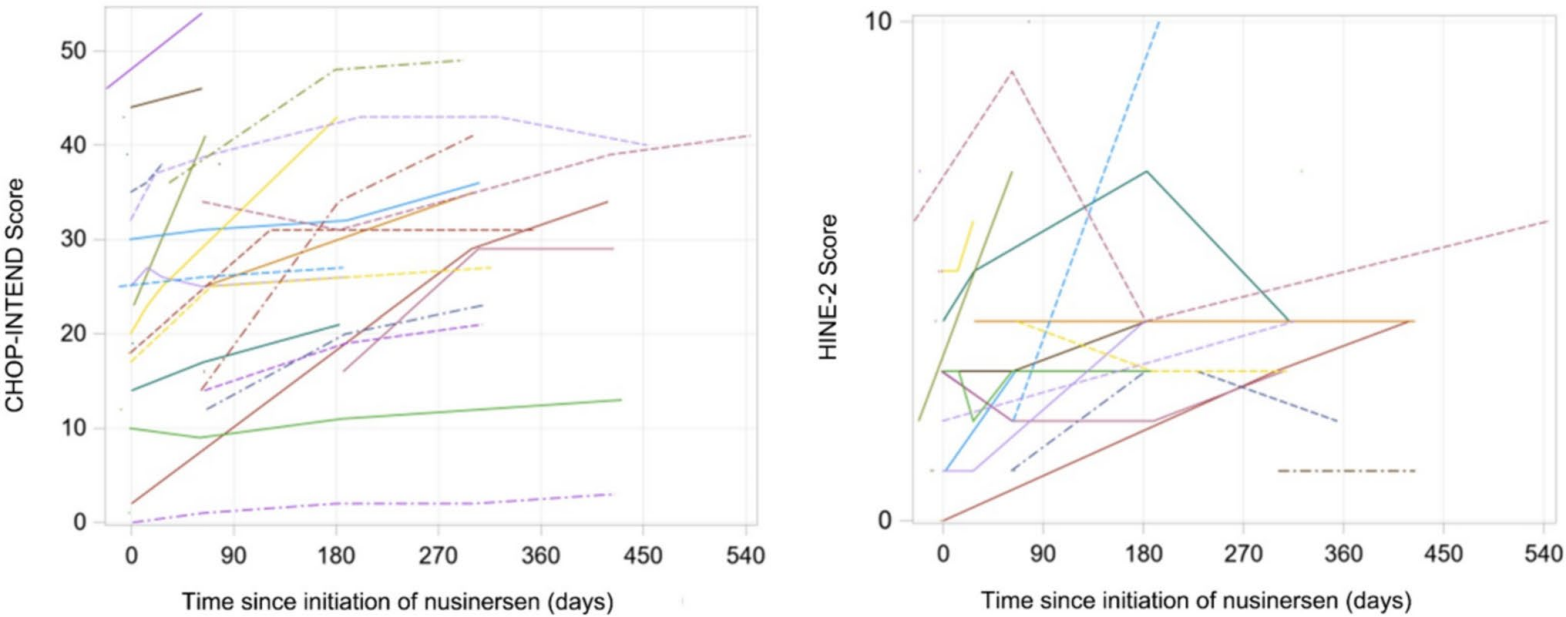
Spaghetti plots of CHOP-INTEND and HINE-2 in patients with SMA Type I. *SMA* Spinal Muscular Atrophy, *CHOP-INTEND* Children’s Hospital of Philadelphia Infant Test of Neuromuscular Disorders, *HINE-2* Hammersmith Infant Neurological Examination Sect. 2

**Fig. 6:**
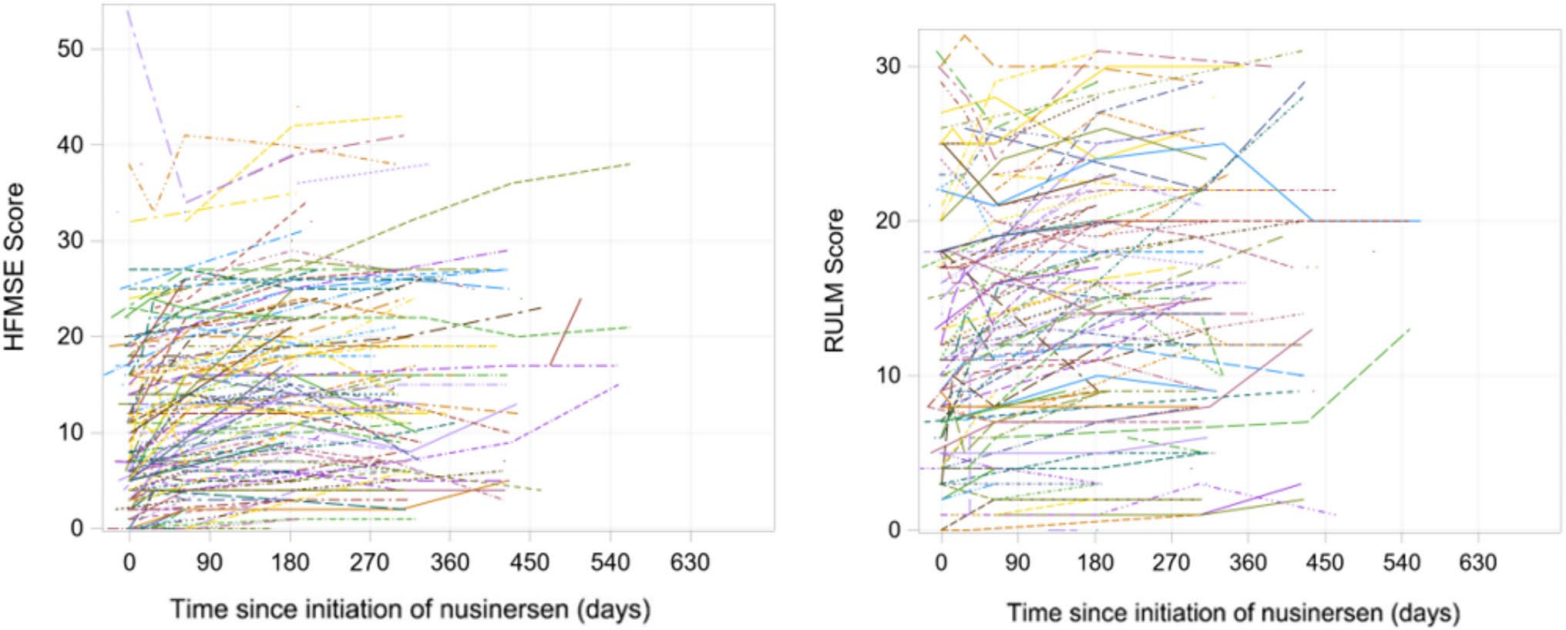
Spaghetti plots of HFMSE and RULM in patients with SMA Type II^a^. **a** The spaghetti plot displays HFMSE and RULM scores for patients with SMA Type II up to 630 days post-nusinersen initiation. Data points after this period have been omitted for clarity. *SMA* Spinal Muscular Atrophy, *HFMSE* Hammersmith Functional Motor Scale-Expanded, *RULM* Revised Upper Limb Module

**Fig. 7:**
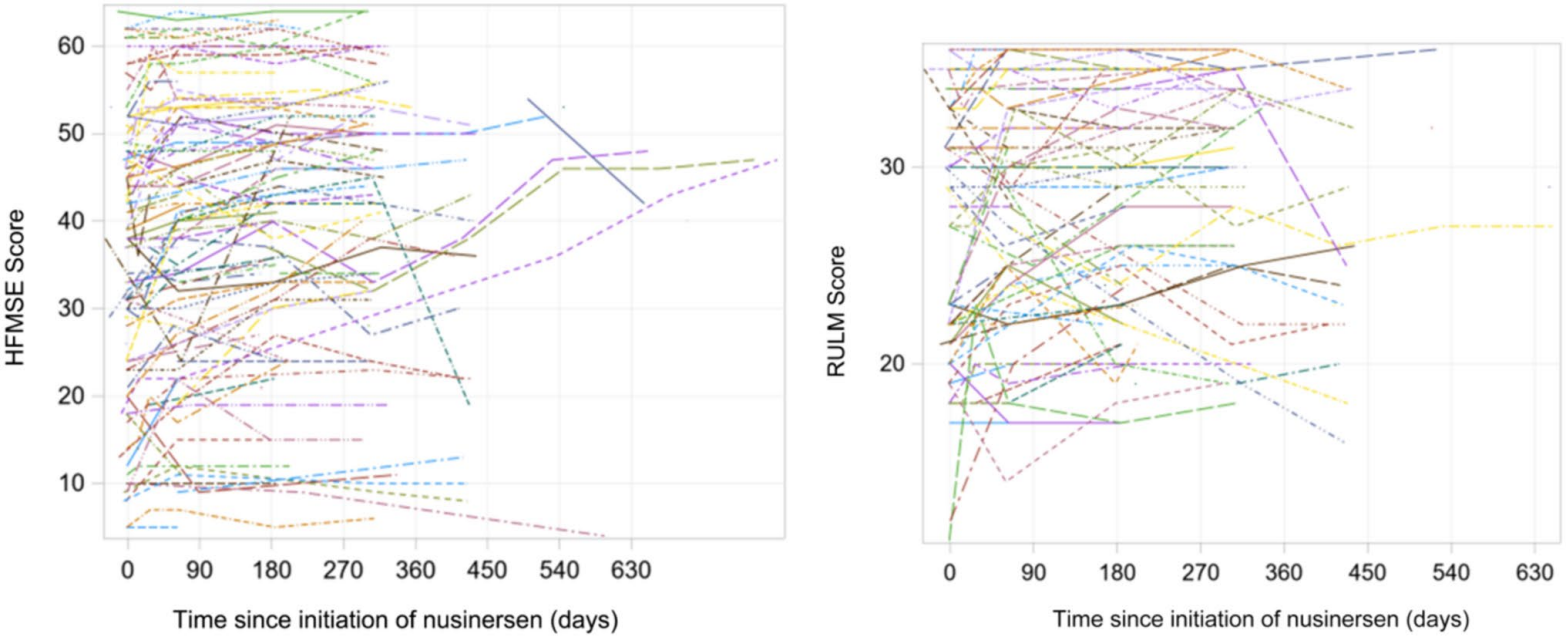
Spaghetti plots of HFMSE and RULM in patients with SMA Type III. *SMA* Spinal Muscular Atrophy, *HFMSE* Hammersmith Functional Motor Scale-Expanded, *RULM* Revised Upper Limb Module

In addition, Figs. 5, 6, 7 depict individual motor measure trajectories for type I, II, and III patients, respectively, according to time since initiation of nusinersen.

### WHO motor milestone/motor function

The WHO motor milestones and motor function achievement at or after nusinersen initiation at patient-level is shown in the appendix. Specifically, within the SMA type I cohort, 4 out of 27 patients exhibited the milestone of ‘sitting without support’ after nusinersen initiation. One of them had this ability at nusinersen initiation, while the initial abilities of the other three patients were unknown. Also, of the 80 type II patients evaluated post-nusinersen for ‘walking alone’, one patient aged at 2 years achieved this milestone, while the other 79 patients were never able to walk alone over time.

### Safety results

Safety results were reported in the analysis among 132 patients initiating nusinersen upon or after enrollment by registry. Of the 132 patients, 62.9% (*n* = 83) experienced at least one AE with a total of 203 AEs recorded. The incidence rate of AEs per patient-year was 2.23, with mild incidents being most common at 1.45, followed by moderate at 0.52, and severe incidents at 0.26. When analyzing according to the System Organ Class (SOC), the most frequent AEs were infections and infestations (37.1%, *n* = 49), with upper respiratory tract infection (15.2%, *n* = 20), pneumonia (9.9%, *n* = 13), and COVID-19 (6.1%, *n* = 8) being the most common. Injury, poisoning, and procedural complications were experienced by 21.2% (*n* = 28) of patients, with procedural pain (13.6%, *n* = 18) being the most reported. Metabolism and nutrition disorders were reported by 9.1% (*n* = 12) of patients, with Vitamin D deficiency being the most common (6.1%, *n* = 8). Respiratory, thoracic, and mediastinal disorders were reported by 6.8% (*n* = 9) of patients. Specifically, post-lumbar puncture syndrome was reported by 21.2% (*n* = 28) of patients.

Of all AEs, only two were deemed related to nusinersen by the investigator. These events included a case of aseptic meningitis in one patient and myalgia in another. The myalgia occurred 2 days post-initial nusinersen administration, lasted for 4 days, and did not necessitate hospitalization. The aseptic meningitis occurred 2 months after the initial dose, lasted for 4 months, and resulted in hospitalization. Neither case required additional medication for management, and both events were classified as mild, resulting in no sequelae. In addition, one patient with SMA type I died from asphyxia during the study, with no established relationship to nusinersen use.

## Discussion

This multi-center investigation, based on the first 5q-SMA registry in China, examined the effects of nusinersen on pediatric patients diagnosed with 5q-SMA and spanning various geographical regions of the country. Regarding effectiveness, the analysis observed a notable trend of improvement in motor measures over most time points. A majority of patients exhibited either stabilized or improved scores following treatment, a deviation from the natural history of SMA, which is generally characterized by a steady, progressive decline across SMA types [22]. Assessment of the nusinersen safety profile supports previously reported findings, showing no new concerns, with only two minor AEs related to nusinersen reported.

Nusinersen was approved in China in 2019, and formally integrated into the NRDL in 2021; however, currently, there is little Chinese-specific research on this topic. Even though SMA is considered a rare disease, the carrier rate of SMA among the Chinese population is approximately 2%, signifying a compelling need for comprehensive studies in this area [23]. Compared to other international studies like registry studies from Japan and Germany, each of which consist of slightly over a hundred patients, our study reports on a large cohort of type II and type III patients, which exceeds what is commonly observed in similar research endeavors [24, 25]. While most studies are limited to measure of short-term outcomes after the initiation of nusinersen treatment, our study offers insights into both short-term and potentially longer term data. Investigation of the response to nusinersen across different SMA types (type I, II, III) offers insights into the drug’s impact on patients with various disease subtypes.

The responses to treatment among patients were generally positive, with the majority of patients showing an improvement or stability in motor function. In patients with SMA type II and type III, the cumulative ‘improved or stable’ rates were generally above 90%, as measured by RULM and HFMSE. This is particularly compelling when considering that patients with SMA type II and type III, as well as their caregivers, have indicated that a stabilization in their current state is a meaningful response meeting their therapeutic expectations [26, 27]. At the 6-month and 10-month marks post-nusinersen treatment, the data show a consistent pattern of improvement or stability, while by the 14-month mark, the patterns became less discernable, possibly due to the limited sample size. The observed improvements in motor measures underscore the beneficial impact of treatment on patient daily functioning. For instance, increased HFMSE scores suggest enhanced motor abilities, likely translating to improved performance in daily activities [17]. Notably, the improvement in RULM scores reflects better upper limb function, potentially enhancing activities such as grasping, lifting, and other hand-related functions post-nusinersen treatment [19]. The magnitude of improvement in motor measure scores in this analysis exceeds those reported in other studies [28, 29]. These variations might be attributed to a range of factors, including differing disease durations at nusinersen initiation, variations in the number of *SMN2* copies, different ages at initiation of treatment, and varying baseline motor status. These results support the potential for improved or at least stable motor function in patients with SMA type II and III.

In the case of patients with SMA type I, none acquired the milestone of ‘sitting without support’ following the initiation of nusinersen treatment. Of note, in contrast with RCTs and other registry studies that typically report a mean baseline age of around 1 year and predominantly feature patients with two *SMN2* copy numbers, our type I patient cohort presents a substantial deviation from these demographics [8, 30, 31]. Specifically, our sample contained a higher proportion of patients aged above 24 months and more than half of the patients with *SMN2* copy numbers of 3 or greater. This disparity primarily arises from our study’s retrospective design, which likely introduces a survivor bias when including type I patients who lived long enough to be entered into the registry. Generally, SMA type I patients who surpass their expected lifespan often experience difficulty in achieving new milestones and demonstrate a diminished response to treatment. This may partially explain why none of our SMA type I patients were able to achieve the milestone of sitting without support. In addition, limited follow-up times and a high rate of missing data may also contribute to the current milestone achievement result. Despite these challenges, it is important to note that these patients still exhibited noticeable benefits from nusinersen treatment. Regarding CHOP-INTEND, a consistent pattern of improvement or stability was discernible in all type I patients, complemented by a positive mean change from the baseline in their HINE-2 scores. Moving forward, these patients will continue to be monitored over a longer term, and additional sensitivity analysis excluding patients initiating nusinersen before enrollment may be conducted to gain deeper insights into the factors that underpin treatment effectiveness.

The safety profile of nusinersen in the analysis cohort exhibited a particularly low incidence of AEs assessed as related to nusinersen by the investigator, which was lower than previously reported in other studies, supporting the safety and tolerability of this therapy [8, 31]. During the study period, two AEs were identified as related to nusinersen, namely aseptic meningitis and myalgia; severity of both events was classified as mild. It is worth noting that assessing relatedness of AEs often relies on the investigator’s judgment, and the lack of uniform and consistent criteria across real-world studies can result in variability. The incidence of post-lumbar puncture syndrome was observed in 21.2% of the patients, consistent with rates reported in previous research [32, 33]. The incidence of post-lumbar puncture syndrome highlights an area for potential improvement in treatment administration or patient management. Despite these considerations, nusinersen continues to show a favorable safety profile in the treatment of SMA, as evidenced by our results and the broader literature [30, 32].

This analysis comes with certain limitations. First, inherent to the study design is the potential for selection bias due to the nature of a disease registry. For example, patients with SMA type I included retrospectively in our registry might represent a group with naturally slower disease progression and longer survival potential than patients who succumbed to comparatively more rapid disease progression [22]. As a result, the direction and extent of motor function improvement observed post-treatment in our study should be interpreted with great caution, since we have predominantly captured data from individuals with potentially longer disease duration since symptom onset, who may experience difficulty in new milestone achievement and response to treatment. Future research efforts addressing clinical queries should aim to prospectively analyze patients newly starting nusinersen to address this bias. Second, incomplete data posed a challenge, especially regarding patients with missing motor measure records. Lack of complete records is common in real-world data collection and reflects our non-interference approach to clinical practices and likely reflects the limited use of outcomes tools by clinicians in China. Consequently, the precision of the analysis might be subtly affected due to incomplete data, especially for patients with SMA type I. Based on our experience, we recommend initiatives to raise clinician awareness of the utility of outcomes measures. Third, the data herein stem from an interim analysis, which is intrinsically accompanied by certain restraints. Considering the relatively recent commencement of the study, the follow-up duration is comparatively short, limiting the depth of insight the analysis can potentially provide regarding long-term effectiveness. This limitation should be organically mitigated as the study proceeds and accrues more extensive long-term data.

In terms of implications for clinical practice and patient care in China, the findings of this analysis could potentially reinforce its role in clinical guidelines and supporting decision-making for inclusion in the NRDL by basic medical insurance in China. Furthermore, the data pinpoint the importance of long-term treatment with nusinersen, highlighting its effectiveness and benefits even in patients with a longer disease course before nusinersen was accessible in China. This underscores a shift in clinical perspectives, bolstering the confidence in the long-term management of SMA of healthcare professionals as well as patients, and emphasizing the necessity of sustained treatment efforts.

The evidence and insights gleaned from this analysis serve to provide direction for future research, underscoring the need for comprehensive exploration in several key areas. These areas encompass: (1) long-term follow-up studies to support the sustained use of nusinersen among patients with SMA; (2) comparative effectiveness research to understand the benefits of different DMTs in various real-world settings; (3) additional subgroup analyses potentially segmented by baseline motor function and concomitant rehabilitation; (4) multivariable analysis pivotal for identifying predictive factors that contribute to better treatment outcomes; (5) effectiveness studies focusing on a variety of outcomes, including compound muscle action potential (CMAP), quality-of-life measures, such as patient-reported outcomes (PROs), and pulmonary function, to offer a more holistic understanding of the impact of nusinersen treatment.

In conclusion, this analysis sheds new light on the use of nusinersen for the treatment of pediatric 5q-SMA in China, demonstrating the drug’s impact on improving or stabilizing motor function, coupled with a positive benefit/risk safety profile. These findings underscore nusinersen’s effectiveness in Chinese pediatric SMA patients and its consistent safety profile, paving the way for optimized treatment strategies and enhancing patient care.

### Supplementary Information

Below is the link to the electronic supplementary material.

Supplementary file1 (PDF 324 KB)

Supplementary file2 (PDF 5091 KB)

Supplementary file3 (PDF 306 KB)

## Data Availability

Requests from qualified investigators for anonymized data not reported in this article should be submitted to https://vivli.org.

## References

[1] Zerres K, Rudnik-Schöneborn S (1995) Natural history in proximal spinal muscular atrophy. Clinical analysis of 445 patients and suggestions for a modification of existing classifications. Arch Neurol 52(5):518–523. 10.1001/archneur.1995.00540290108025 10.1001/archneur.1995.00540290108025 7733848

[2] Darras BT, De Vivo DC (2018) Precious SMA natural history data: a benchmark to measure future treatment successes. Neurology 91(8):337–339. 10.1212/WNL.0000000000006026 30045956 10.1212/WNL.0000000000006026

[3] Darras BT, Finkel RS (2017) Chapter 25 - Natural history of spinal muscular atrophy. In: Sumner CJ, Paushkin S, Ko CP (ed), Spinal muscular atrophy, Academic Press, pp. 399–421. 10.1016/B978-0-12-803685-3.00025-2

[4] Chan SHS, Wong CKH, Wu T et al (2022) Significant healthcare burden and life cost of spinal muscular atrophy: real-world data. Eur J Health Econ 24(8):1373–1382. 10.1007/s10198-022-01548-5 36403177 10.1007/s10198-022-01548-5 PMC10533630

[5] Neil EE, Bisaccia EK (2019) Nusinersen: a novel antisense oligonucleotide for the treatment of spinal muscular atrophy. J Pediatr Pharmacol Ther 24(3):194–203. 10.5863/1551-6776-24.3.194 31093018 10.5863/1551-6776-24.3.194 PMC6510522

[6] Mahajan R (2019) Onasemnogene Abeparvovec for spinal muscular atrophy: the costlier drug ever. Int J Appl Basic Med Res 9(3):127–128. 10.4103/ijabmr.IJABMR_190_19 31392173 10.4103/ijabmr.IJABMR_190_19 PMC6652281

[7] Ratni H, Scalco RS, Stephan AH (2021) Risdiplam, the first approved small molecule splicing modifier drug as a blueprint for future transformative medicines. ACS Med Chem Lett 12(6):874–877. 10.1021/acsmedchemlett.0c00659 34141064 10.1021/acsmedchemlett.0c00659 PMC8201486

[8] Hagenacker T, Wurster CD, Günther R et al (2020) Nusinersen in adults with 5q spinal muscular atrophy: a non-interventional, multicentre, observational cohort study. Lancet Neurol 19(4):317–325. 10.1016/S1474-4422(20)30037-5 32199097 10.1016/S1474-4422(20)30037-5

[9] Pechmann A, Langer T, Schorling D et al (2018) Evaluation of children with SMA type 1 under treatment with nusinersen within the expanded access program in Germany. J Neuromuscul Dis 5(2):135–143. 10.3233/JND-180315 29689734 10.3233/JND-180315 PMC6004898

[10] Pechmann A, Behrens M, Dörnbrack K et al (2022) Improved upper limb function in non-ambulant children with SMA type 2 and 3 during nusinersen treatment: a prospective 3-years SMArtCARE registry study. Orphanet J Rare Dis 17(1):384. 10.1186/s13023-022-02547-8 36274155 10.1186/s13023-022-02547-8 PMC9589836

[11] Pechmann A, Behrens M, Dörnbrack K et al (2023) Effect of nusinersen on motor, respiratory and bulbar function in early-onset spinal muscular atrophy. Brain 146(2):668–677. 10.1093/brain/awac252 35857854 10.1093/brain/awac252

[12] Yang H, Tao Q, Li D et al (2023) Assessment of motor function and nutritional status in children with spinal muscular atrophy treated with nusinersen after loading period in Western China: a retrospective study. BMC Neurol 23(1):35. 10.1186/s12883-023-03063-3 36690929 10.1186/s12883-023-03063-3 PMC9869561

[13] Chen L, Liu F, Fang D, Li J (2023) Study on the efficacy, safety, and biomarkers of nusinersen in type II and III spinal muscular atrophy in children. Front Pediatr 11:1294405. 10.3389/fped.2023.1294405 38111627 10.3389/fped.2023.1294405 PMC10725990

[14] Mao SS, Feng YJ, Xu L et al (2022) Clinical follow-up analysis of nusinersen in the disease-modifying treatment of pediatric spinal muscular atrophy. Chin J Pediatr 60(7):688–693. 10.3760/cma.j.cn112140-20211223-01075 10.3760/cma.j.cn112140-20211223-01075 35768357

[15] Wang Y, Wu L, Hong S et al (2022) A national registry for pediatric patients with spinal muscular atrophy in China: Design, progress and patient characteristics. 25th National Conference of Neurology, China

[16] Glanzman AM, Mazzone E, Main M et al (2010) The Children’s Hospital of Philadelphia Infant Test of Neuromuscular Disorders (CHOP-INTEND): test development and reliability. Neuromuscul Disord 20(3):155–161. 10.1016/j.nmd.2009.11.014 20074952 10.1016/j.nmd.2009.11.014 PMC3260046

[17] Main M, Kairon H, Mercuri E, Muntoni F (2003) The Hammersmith functional motor scale for children with spinal muscular atrophy: a scale to test ability and monitor progress in children with limited ambulation. Eur J Paediatr Neurol 7(4):155–159. 10.1016/s1090-3798(03)00060-6 12865054 10.1016/s1090-3798(03)00060-6

[18] De Sanctis R, Coratti G, Pasternak A et al (2016) Developmental milestones in type I spinal muscular atrophy. Neuromuscul Disord 26(11):754–759. 10.1016/j.nmd.2016.10.002 27769560 10.1016/j.nmd.2016.10.002 PMC5091285

[19] Mazzone ES, Mayhew A, Montes J et al (2017) Revised upper limb module for spinal muscular atrophy: development of a new module. Muscle Nerve 55(6):869–874. 10.1002/mus.25430 27701745 10.1002/mus.25430

[20] WHO Multicentre Growth Reference Study Group (2006) WHO Motor Development Study: Windows of achievement for six gross motor development milestones. Acta Paediatr Suppl 450:86–95. 10.1111/j.1651-2227.2006.tb02379.x 16817682 10.1111/j.1651-2227.2006.tb02379.x

[21] World Health Organization (2023) The WHO Child Growth Standards (2023) World Health Organization. https://www.who.int/tools/child-growth-standards/standards

[22] Ou S, Ho C, Lee W, Lin K, Jones CC, Jong Y, SMA Study Group (2021) Natural history in spinal muscular atrophy type I in Taiwanese population: a longitudinal study. Brain Dev 43(1):127–134. 10.1016/j.braindev.2020.07.012 10.1016/j.braindev.2020.07.012 32878721

[23] Li C, Geng Y, Zhu X et al (2020) The prevalence of spinal muscular atrophy carrier in China. Medicine (Baltimore) 99(5):e18975. 10.1097/MD.0000000000018975 32000428 10.1097/MD.0000000000018975 PMC7004774

[24] Sahashi K, Hashizume A, Kuwatsuka Y et al (2022) The Japan registry for adult subjects of spinal muscular atrophy (jREACT-SMA): protocol for a longitudinal observational study. JMIR Res Protoc 11(12):e38878. 10.2196/38878 36520510 10.2196/38878 PMC9801261

[25] Leibrock B, Landfeldt E, Hussong J et al (2023) Areas of improvement in the medical care of SMA: evidence from a nationwide patient registry in Germany. Orphanet J Rare Dis 18(1):32. 10.1186/s13023-023-02639-z 36810103 10.1186/s13023-023-02639-z PMC9945617

[26] Pera MC, Coratti G, Forcina N et al (2017) Content validity and clinical meaningfulness of the HFMSE in spinal muscular atrophy. BMC Neurol 17(1):39. 10.1186/s12883-017-0790-9 28231823 10.1186/s12883-017-0790-9 PMC5324197

[27] Rouault F, Christie-Brown V, Broekgaarden R et al (2017) Disease impact on general well-being and therapeutic expectations of European type II and type III spinal muscular atrophy patients. Neuromuscul Disord 27(5):428–438. 10.1016/j.nmd.2017.01.018 28237437 10.1016/j.nmd.2017.01.018

[28] Scheijmans FEV, Cuppen I, van Eijk RPA et al (2022) Population-based assessment of nusinersen efficacy in children with spinal muscular atrophy: a 3-year follow-up study. Brain Commun 4(6):fcac269. 10.1093/braincomms/fcac269 10.1093/braincomms/fcac269 PMC9651026 36382221

[29] Pera MC, Coratti G, Bovis F et al (2021) Nusinersen in pediatric and adult patients with type III spinal muscular atrophy. Ann Clin Transl Neurol 8(8):1622–1634. 10.1002/acn3.51411 34165911 10.1002/acn3.51411 PMC8351459

[30] Finkel RS, Mercuri E, Darras BT et al (2017) Nusinersen versus sham control in infantile-onset spinal muscular atrophy. N Engl J Med 377(18):1723–1732. 10.1056/NEJMoa1702752 29091570 10.1056/NEJMoa1702752

[31] Belančić A, Strbad T, Kučan Štiglić M, Vitezić D (2023) Effectiveness of nusinersen in type 1, 2 and 3 spinal muscular atrophy: croatian real-world data. J Clin Med 12(8):2839. 10.3390/jcm12082839 37109175 10.3390/jcm12082839 PMC10142582

[32] Mercuri E, Darras BT, Chiriboga CA et al (2018) Nusinersen versus sham control in later-onset spinal muscular atrophy. N Engl J Med 378(7):625–635. 10.1056/NEJMoa1710504 29443664 10.1056/NEJMoa1710504

[33] Ellenby MS, Tegtmeyer K, Lai S, Braner DAV (2006) Lumbar puncture. N Engl J Med 355(13):e12. 10.1056/NEJMvcm054952 17005943 10.1056/NEJMvcm054952

