# Supplementary material for: Nusinersen effectiveness and safety in pediatric patients with 5q-spinal muscular atrophy: a multi-center disease registry in China
Source: J Neurol. 2024 Jul 2;271(8):5378–91. doi: 10.1007/s00415-024-12442-w (PMC11319379; doi:10.1007/s00415-024-12442-w)
Supplement: Supplementary file 1 — Supplementary file1 (PDF 324 KB) [file 415_2024_12442_MOESM1_ESM.pdf]

**REGULATORY SPONSOR:**	Biogen Biotechnology (Shanghai) Co., Limited<br>Room 2106-11, 21F<br>No.288 South Shaanxi Road<br>Xuhui District, Shanghai<br>China
**TRACKING NUMBER:**	**CN-SMG-11774**
**PHASE OF DEVELOPMENT:**	**N/A**

TITLE: Pediatric Patients with Spinal Muscular Atrophy in China: A Nationwide Registry

DATE: 04 July 2022
Version 2.0

FINAL

CONFIDENTIAL
The information contained herein may not be used, disclosed, or published without the written consent of Biogen

SPONSOR INFORMATION

Biogen Biotechnology (Shanghai) Co., Limited
Room 2106-11, 21F
No.288 South Shaanxi Road
Xuhui District, Shanghai
China

Primary contact for urgent medical issues:

CRO name	IQVIA
CRO phone number:	+86 (21) 3325 2288

Biogen may transfer any or all of its study-related responsibilities to contract research organization (CRO) and other third parties, however, Biogen retains overall accountability for these activities

CONFIDENTIAL
The information contained herein may not be used, disclosed, or published without the written consent of Biogen

MEDICAL DIRECTOR SIGNATURE PAGE

Protocol CN-SMG-11774 was approved by:

Zhenfei Huang 2022.7.11

Zhenfei Huang
Head of Medical Affairs in China
Biogen Biotechnology (Shanghai) Co., Limited

Date

TABLE OF CONTENTS

SPONSOR INFORMATION.....2
MEDICAL DIRECTOR SIGNATURE PAGE.....3
1. KEY STUDY ELEMENTS.....7
1.1. Synopsis.....7
1.2. Study Design Schematic.....9
1.3. Recommended Schedule of Activities.....9
2. LIST OF ABBREVIATIONS AND DEFINITIONS.....12
3. INTRODUCTION.....13
3.1. Study Rationale.....13
3.2. Background.....13
3.2.1. Overview of Disease.....13
3.2.2. Available Therapies for Disease.....14
3.3. Benefit-Risk Assessment.....14
4. STUDY OBJECTIVES AND ENDPOINTS.....15
5. STUDY DESIGN.....16
5.1. Study Overview.....16
5.2. Study Duration for Participants.....16
5.3. Study Stopping Rules.....16
5.4. End of Study.....16
6. STUDY POPULATION.....17
6.1. Inclusion Criteria.....17
6.2. Exclusion Criteria.....17
6.3. Screening.....17
7. STUDY TREATMENT.....18
7.1. Study Treatment Management and Administration.....18
7.2. Concomitant Therapy.....18
8. WITHDRAWAL OF PATIENTS FROM THE STUDY.....19
8.1. Lost to Follow-Up.....19
9. DATA COLLECTION.....20
10. SAFETY DEFINITIONS, RECORDING, REPORTING, AND RESPONSIBILITIES.....22
10.1. Definitions.....22
10.1.1. Adverse Event.....22

CONFIDENTIAL
The information contained herein may not be used, disclosed, or published without the written consent of Biogen

10.1.2. Serious Adverse Event 22
10.2. Safety Classifications 23
10.2.1. Investigator Assessment of Events 23
10.2.2. Relationship of Events to DMTs 23
10.2.3. Severity of Events 24
10.2.4. Expectedness of Events 24
10.3. Recording of Events 24
10.3.1. Adverse Events 24
10.3.2. Serious Adverse Events 24
10.3.2.1. Deaths 25
10.3.3. Reporting Events 25
10.4. Procedures for Handling Special Situations 25
10.4.1. Overdose 25
10.4.2. Reporting Pregnancy 25
10.4.3. Coordination With the Pregnancy Registry 26
10.5. Contraception 26
10.6. Medical Emergency 26
10.7. Investigator Responsibilities 26
10.7.1. Investigator 26
10.7.2. Biogen’s Responsibilities 26
11. STATISTICAL CONSIDERATIONS 28
11.1. General Considerations 28
11.2. Analysis Sets 28
11.3. Methods of Analysis 28
11.4. Interim Analyses 28
11.5. Sample Size Justification 28
12. ETHICAL REQUIREMENTS 29
12.1. Declaration of Helsinki 29
12.2. Ethics Committee 29
12.3. Changes to Final Study Protocol 30
12.4. Participant Information and Consent 30
12.5. Participant Data Protection 30
12.6. Compensation for Injury 31
12.7. Conflict of Interest 31
12.8. Study Report Signatory 31

12.9. Registration of Study and Disclosure of Study Results 31
12.10. Retention of Study Data 31
13. KEY ROLES AND STUDY GOVERNANCE COMMITTEES 32
13.1. Vendors 32
13.1.1. Contract Research Organization 32
13.1.2. Electronic or Remote Data Capture 32
14. ADMINISTRATIVE PROCEDURES 33
14.1. Study Site Initiation 33
14.2. Quality Control and Assurance 33
14.3. Monitoring of the Study 33
14.4. Study Funding 34
14.5. Publications 34
15. REFERENCES 35
16. SIGNED AGREEMENT OF THE STUDY PROTOCOL 36

1. KEY STUDY ELEMENTS

1.1. Synopsis

Tracking number:	CN-SMG-11774
Protocol Title:	Pediatric Patients with Spinal Muscular Atrophy in China: A Nationwide Registry
Version Number:	2.0
Phase of Development:	N/A
Study Indication:	Spinal Muscular Atrophy (SMA)
Study Rationale:	The planned registry project will provide a platform to collect longitudinal clinical routine data on Chinese SMA patients. Items for data collection will be aligned with TREAT-NMD. SMA has a broad spectrum of severity and clinical trials only cover a subgroup of these. Therefore there is a strong need to monitor the clinical status of all treated and untreated SMA in China. The registry will facilitate the understanding of disease progression and treatment effectiveness for Chinese pediatric patients diagnosed with SMA.
Study Objectives and Endpoints:	The primary objective of the study is to describe the natural history and utilization of DMT among pediatric Chinese patients with 5q-SMA.<br>The primary endpoints that relate to this objective are not applicable.<br>Additional objectives and endpoints are as follows:<br>• To describe the effectiveness of disease modifying therapy (DMT)<br>o Time to mortality, motor function, motor measures, pulmonary function, scoliosis, hospitalizations and comorbidities, clinical observations, patient reported outcomes, wheelchair use, nutrition and electrophysiology and biomarkers<br>• To describe the safety of DMT<br>o Adverse events and serious adverse events
Study Design:	This is a multicenter registry study that will examine SMA natural history and DMT outcomes in a real-world setting

CONFIDENTIAL
The information contained herein may not be used, disclosed, or published without the written consent of Biogen

	both prospectively and retrospectively. Patients will be observed based on regular prescription and visit schedules.
Study Location:	Approximately 20 to 25 sites in China are planned.
Study Population:	Pediatric SMA patient in China.<br>Detailed criteria are described in Section 6.1 and 6.2.
Number of Planned Patients:	Approximately 600 patients will be enrolled.
Treatment Supply	Nusinersen will not be supplied for this study. The study will observe patients who have been prescribed DMT treatments by their physician (Investigator) according to the local Prescribing Information.
Visit Schedule:	The data collected at enrollment will coincide with a routine clinical visit to the Investigator. Subsequent data will also be collected during routine clinical visits for up to 60 months from enrollment. For patients who received nusinersen, visits of the first four loading doses are anticipated to occur on day 0, day 14, on day 28, and on day 63 and the following maintenance doses are anticipated to occur at approximately every 4 months. Among untreated patients or those treated with other DMTs, first three visits are anticipated to occur on day 0, day 90 and on day 180, and then occurring at approximately every 6 months. Visits may vary according to local standards of care.
Risk-Benefit Analysis:	No additional risks will be imposed on participants other than the existing risks present with the treatment or the disease, because the study will only observe regular clinical practice.

1.2. Study Design Schematic

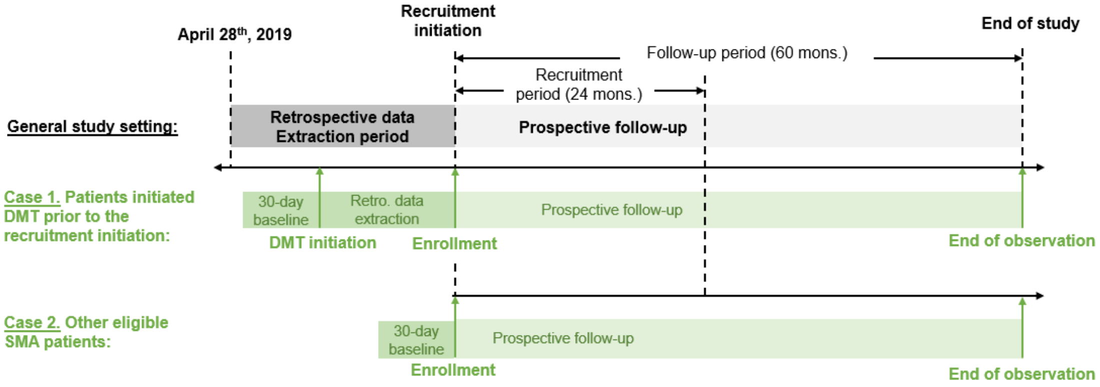

Figure 1. Diagram of the registry design for data collection

Note:

1. The study will retrospectively collect available data for patients who initiated DMT prior to recruitment initiation starting from index date. Index date will not be earlier than April 28th, 2019, which was the launch date of nusinersen, the first DMT in China. Baseline data will be collected within 30 days prior to the index date.
2. The index date for patients treated with any DMTs will be the date of DMT initiation, and the index date for those untreated patients will be the date of study enrollment.

1.3. Recommended Schedule of Activities

Visit schedules for the group of SMA patients treated with DMT (including nusinersen and other DMTs) and the group of untreated patients will be described in Table 1. Data for the registry will only be collected within regular SMA patient visits.

CONFIDENTIAL
The information contained herein may not be used, disclosed, or published without the written consent of Biogen

Table 1. Expected Tests and Assessments

**Tests and Assessments**	Enrollment data collection points	Follow-up data collection points<br>*For nusinersen-treated patients, it is expected they will be observed on days 14, 28, and 63 and every 4 months thereafter.*<br>*For untreated patients or those on other DMTs, it is expected they will be observed on days 90* [a] *and 180 and every 6 months thereafter.*
	V0	V1-V17
Informed Consent	X	
Review of eligibility criteria	X	
Demographics	X	
Living status	X	X
Genetic diagnosis	X	
Clinical observations	X	X
Scoliosis	X	X
Motor function	X	X
Wheelchair use[b]	X	X
Nutrition	X	X
Pulmonary function[c]	X	X
Therapies and medications (including DMTs other than nusinersen)	X	X
Hospitalizations and comorbidities	X	X
Clinical research	X	X
Motor measures	X	X
Patient reported outcomes	X	X
Electrophysiology and biomarkers	X	X
Nusinersen/other DMT treatment utilization[d]	X	X
Adverse events / Serious adverse events	X	X
End of data collection		X

X: based on the patient's routine clinical visit, time points the study visits will most likely happen. No extra study visits, examinations, laboratory tests or procedures will be mandated

a. In children with SMA type 1 < 2 years of age

b. For patients ≥ 2 years old

c. If the patient is sufficiently cooperative due to age

d. For patients treated with nusinersen or other DMTs

2. LIST OF ABBREVIATIONS AND DEFINITIONS

6-MWT	6-Minute Walk Test
AE	adverse event
CGI-S	Clinical Global Impression of Severity
CHOP-Intend	Children's Hospital of Philadelphia Infant Test of Neuromuscular Disorders
CRF	case report form
CRO	contract research organization
DMT	disease modifying therapy
ECG	electrocardiogram
EDC	electronic data capture
EOS	end of study
FVC	Forced Vital Capacity
FPFV	fist patient first visit
FU	follow up
GPP	Good Pharmacoepidemiology Practice
HINE	Hammersmith Infant Neurological Examination
HFMSE	Hammersmith Functional Motor Scale Expanded
ICF	informed consent form
ICH	International Council for Harmonisation
IDMC	independent data monitoring committee
IEC	independent ethic committee
IPPV	intermittent positive-pressure ventilation
NIV	non-invasive ventilation
NMD	neuromuscular disease
NMPA	National Medical Products Administration
NSAE	Nonserious adverse event
PRO	patient-reported outcomes
QC	quality control
RULM	Revised Upper Limb Module
SAE	serious adverse event
SAP	statistical analysis plan
SAS	statistical analysis system
SMA	spinal muscular atrophy
SMN	survival motor neuron
TGI	Total Global Impression
WHO	World Health Organization

3. INTRODUCTION

3.1. Study Rationale

Due to the improvements in treatment and technological advances, the natural history of SMA patients has changed. Especially in rare diseases a comprehensive and systematic collection of data regarding the natural history of the disease and the influence of different drug treatments is crucial to improve the care of these patients. So far, current clinical trials regarding new therapeutic approaches for SMA patients only cover a subgroup of the broad spectrum of severity of SMA. Thus, there are strong unmet needs to monitor all treated and untreated SMA patients and particularly in China, given the relatively large number of underserved Chinese patients. The registry will facilitate the understanding of disease progression and treatment effectiveness for Chinese pediatric patients diagnosed with SMA.

The aim of this registry is to both retrospectively collect and prospectively monitor pediatric SMA patients in the long term to achieve a better understanding of the natural history and outcomes of patients. The planned SMA registry will provide an online platform to collect longitudinal data on pediatric SMA patients. As a disease-specific system, the database will collect data from all available pediatric SMA patients independent of their actual treatment regimen. Items for data collection are aligned with TREAT-NMD [6,7].

3.2. Background

3.2.1. Overview of Disease

Spinal muscular atrophy (SMA) is a neuromuscular disorder that is primarily characterized by degeneration of the anterior horn cells of the spinal cord resulting in muscle atrophy and muscle weakness. It is an autosomal-recessive disorder caused by a homozygous deletion or mutation in the survival motor neuron 1 (SMN1) gene on chromosome 5q13. With an incidence of 1:6,000 to 1:10,000, SMA is among the rare diseases[1]. The severity of the disease is highly variable and correlates with the age of onset. SMA is classified into four different subtypes (Table 2).

Table 2: Classification of different phenotypes of SMA

Type	Age of onset (% of SMA patients)	Best motor function	Life expectancy with a natural course of disease	Clinical features
1	1-6 months (50-60%)	Never sits	< 2 years	Muscle weakness, floppy infant, weak cough, difficulties in swallowing, areflexia, tongue fasciculations, paradoxical breathing
2	7-18 months (30%)	Sits but never walks	> 2 years	Delay in motor development, muscle weakness, fine tremor in upper extremities, weak cough, scoliosis, joint contractions

3	> 18 months (10%)	Walks	Adulthood	Variable muscle weakness, scoliosis, joint contractions, loss of the ability to walk
4	10-30 years (1%)	Walks	Adulthood	Variable but mild muscle weakness

3.2.2. Available Therapies for Disease

Disease management is mainly based on a multidisciplinary management to improve motor, respiratory, gastrointestinal, and orthopedic symptoms. With technical advancements and thus the possibility to provide non-invasive ventilator support and enteral feeding to the affected patients, survival has increased[2,3]. In 2017, a consensus statement for standards of care in SMA was published[4], nonetheless, there is high variability in the standards of care for patients with SMA.

Current therapeutic strategies include mainly respiratory, feeding and orthopedic management and supportive pharmacologic therapy. These therapies improve muscular function, quality of life and longevity, but do not directly target the disease mechanism. Presently in China, there is one registered and approved therapy to treat 5q-SMA patients (nusinersen). This compound is an antisense oligonucleotide targeting the splicing of exon 7 of the SMN2 gene, aiming at increasing the production of a full-length SMN protein. This compound is administered through intrathecal administration, with 4 loading doses over 63 days, followed by maintenance doses every 4 months. New therapeutic compounds, gene therapy (AVXS-101, Avexis) was approved by FDA and EMA recently. Small molecule targeting the splicing of SMN2 was also approved by FDA and EMA.

Based upon a better understanding of the molecular genetics of SMA, there has been a promising approach for the development of novel drugs intervening with the pathophysiology of SMA. Nusinersen is the first drug that has been approved for the treatment of 5q-SMA. It was approved in the United States in December 2016, in Europe in June 2017, and in China in February 2019.

3.3. Benefit-Risk Assessment

The study results on natural history and treatment patterns will provide better understanding of SMA prognosis and effective treatment interventions. The study will only observe regular clinical practice, and therefore, no additional health risks will be imposed on participants other than the existing risks present with the treatment or the disease.

The potential privacy risks to health data related to participation in this study are justified by the anticipated benefit to advance medical knowledge.

For patients on DMTs, detailed information about the known and expected benefits and risks and reasonably expected adverse events (AEs) of those DMTs is available in their respective Investigator's Brochure(s) or Prescribing Information.

CONFIDENTIAL
The information contained herein may not be used, disclosed, or published without the written consent of Biogen

4. STUDY OBJECTIVES AND ENDPOINTS

The objective of this registry is to longitudinally follow up pediatric patients (aged <18) with 5q-SMA to obtain information on both the natural history of the disease (from patients who received supportive care only) and the effectiveness and safety of disease modifying therapies (DMT), nusinersen in particular. Disease characteristics, treatment patterns, and outcomes (please refer to the section of outcome measurements) will be collected by extracting relevant information from medical records. Since this is not an experimental study, treatment plans will not be altered by participation, and all clinical managements including supportive care, DMTs, and concomitant medications will be administered based on healthcare providers' clinical judgements without stakeholders' interventions.

Currently, three DMT drugs are available including nusinersen, onasemnogene abeparvovec, and risdiplam in the US, but only nusinersen is approved by the National Medical Products Administration (NMPA) in China. In line with the main objective of this registry, and in case there will be more NMPA-approval DMTs in the future, in addition to nusinersen, this registry will also collect information on other DMTs once they become available in China.

**Primary Objective**	**Primary Endpoints**
To describe the natural history and utilization of DMT among pediatric Chinese patients with 5q-SMA.	Not applicable.
**Additional Objectives**	**Additional Endpoints**
To describe the effectiveness of DMT.	Time to mortality, motor function, motor measures, pulmonary function, scoliosis, hospitalizations and comorbidities, clinical observations, patient reported outcomes, wheelchair use, nutrition and electrophysiology and biomarkers.
To evaluate the safety of DMT.	Adverse events and serious adverse events.

5. STUDY DESIGN

5.1. Study Overview

This is a prospective, multicenter, non-randomized registry for treated and untreated pediatric patients with SMA in a real-world setting. Data obtained within regular patient visits prior to registry inclusion may be documented retrospectively. The study plans to recruit approximately 600 pediatric patients from 20-25 sites in China.

The current aim of this registry is to include centers in China meeting the structural and personnel requirements for performing the planned regular registry-related investigations. These reference centers in China will be selected from COEs which 1) have already been well trained on motor function evaluation, 2) have the potential to enroll and follow reasonable patient numbers, 3) have experience in treating SMA with nusinersen, and 4) have experience in conducting clinical trials. The participation of these centers in the data collection for this study will improve cooperation and harmonization between pediatric care centers for patients with spinal muscular atrophy.

See Figure 1 for a schematic of the study design.

5.2. Study Duration for Participants

The study period will consist of identification and enrollment of participants meeting the eligibility criteria, observation of their treatment, and their follow-up. The total duration of study participation for each participant will be up to 60 months.

5.3. Study Stopping Rules

The Sponsor may terminate this study at any time, after informing the Investigators. The Sponsor will notify the Investigators and its partner (if appropriate) when the study is to be placed on hold, completed, or terminated.

5.4. End of Study

The end of study is last participant, last visit, for final collection of data.

CONFIDENTIAL
The information contained herein may not be used, disclosed, or published without the written consent of Biogen

6. STUDY POPULATION

Subjects will be included in the registry, following diagnosis, as long as all inclusion criteria and no exclusion criteria are fulfilled.

6.1. Inclusion Criteria

To be eligible to participate in this study, candidates must meet the following eligibility criteria at Day 0, or at the time point specified in the individual eligibility criterion listed:

1. Ability of the participant and/or his/her legally authorized representative (e.g., parent or legal guardian), as appropriate and applicable, to understand the purpose and risks of the study, to provide informed consent, and to authorize the use of confidential health information in accordance with national and local privacy regulations.
2. Genetically confirmed 5q-SMA.
3. Age < 18 years at enrollment.

6.2. Exclusion Criteria

Candidates will be excluded from study entry if any of the following exclusion criteria exist at Day 0, or at the time point specified in the individual criterion listed:

1. Unable or unwilling to provide informed consent.
2. Other types of SMA (non 5q-SMA).

6.3. Screening

Once informed consent is obtained, a unique identification number is assigned that will be used on study-related documents pertaining to the participant. Any identification numbers that are assigned will not be reused even if the participant does not continue in the study. Study sites are required to document all screened participants initially considered for inclusion in the study.

7. STUDY TREATMENT

7.1. Study Treatment Management and Administration

As a disease registry, there is no study treatment. No DMT will be supplied by The Sponsor for this study. Any DMT must be administered according to standard local prescribing procedures or the instructions in the approved Prescribing Information.

7.2. Concomitant Therapy

Prescribed concomitant medications and therapies including physiotherapy will be recorded from the index date. Concomitant medications and therapies used together with any reported AEs or SAEs during the same period also need to be documented. The required details of documentation include drug or therapy name and dosage (amount, frequency, duration).

Concomitant medications and therapies will be listed in the eCRF.

8. WITHDRAWAL OF PATIENTS FROM THE STUDY

The registry patient can have his/her participation in the registry terminated prematurely at any time, without having to give reasons. Patients must be withdrawn from the study for any one of the following reasons:

- The participant withdraws consent.
- The participant is unwilling or unable to comply with the protocol.
- The physician withdraws the participant from the study for medical reasons.

The treating physician has the right to terminate registry participation of a patient according to the following conditions:

- Loss of contact.
- Logistical reasons (patient changes his/her doctor or hospital or moves to another location).
- Extreme circumstances arise which make any registry-relevant follow-up impossible.

The reason for the participant's withdrawal from the study must be recorded in the participant's case report form (CRF).

The investigator has the right to terminate the registry at one of the centers. The registry can be terminated prematurely at his center by the investigator if, for instance, unforeseeable circumstances have arisen at the trial center which preclude the continuation of the registry or the investigator considers that the resources for continuation are no longer available. The sponsor can initiate the exclusion of a center from further participation if, serious problems arise with regard to the quality of the collected data which cannot be resolved. Premature termination at one of the trial centers does not automatically mean a termination of the registry for already enrolled registry patients. The documentation of already enrolled registry patients will be reviewed for completeness and plausibility. Queries may be raised for further clarification before the center is closed. These queries must be answered properly by the center.

The sponsor/coordinating investigator is under obligation to monitor the progress of the registry about intended developments and, if necessary, initiate the termination of the entire registry. The sponsor/coordinating investigator will be supported in this responsibility by the contract research organization, if necessary. Further, the coordinating investigator can prematurely terminate the registry if sufficient financial funding is not available anymore. All published data will be stored and archived for 10 years.

8.1. Lost to Follow-Up

Patients will be considered lost to follow-up if they repeatedly fail to return for routine clinic visits and are unable to be contacted by the study site. Contact attempts should be documented in the participant's medical record. Should the participant continue to be unreachable, that participant will be considered to have withdrawn from the study with a primary reason of lost to follow-up.

Reasons for loss to follow-up should be documented by the physician in charge in the respective section of the CRF. Specific recommendations for the respective follow-up visits of patients to evaluate other treatments or interventions will be added to the schedule of activities.

9. DATA COLLECTION

To avoid selection bias, physicians are asked to include all patients fulfilling the eligibility criteria in a consecutive way. The contract research organization will be informed about newly included patients via a registration form. Each patient will be identified in the registry by a unique patient identification code (patient number). A system to prevent duplicated patient entries will be implemented. A subject identification log with the names of all registry patients with the corresponding identification code assigned to each patient is kept by each center.

Data for the registry will be obtained within regular, clinically recommended routine visits of SMA patients, depending on their current treatment regimen. Data obtained within regular patient visits prior to registry inclusion will be documented retrospectively. Recommended time points for these visits are listed in the Flow Chart.

All Information on the registry, procedures and documents are provided to the participating sites by the contract research organization (CRO). The participating physician or designated person will

- inform the patient or his/her legal representative about the registry and ask for written consent. The original consent form will be filed with the patient's source documents; a copy stays with the patient.
- fill in the standardized clinical eCRF.
- inform the CRO in case that the patient withdraws his/her consent.

This registry study will include both retrospective and prospective data collection. Eligible SMA patients will be recruited during the 24-month recruitment period and will be prospectively followed up to the end of study or deaths or willing to drop out, whichever comes first. If patients received DMT (including nusinersen and other DMTs) prior to the recruitment initiation and post April 28th, 2019 (as the date the first DMT, nusinersen, was launched in Chinese market), data will be retrospectively collected via medical chart review. Retrospective data collection will be considered complete for a participating patient if data of last available medical chart or latest survival status before recruitment initiation have been recorded in the electronic case report form (eCRF). Otherwise, patients will be prospectively followed up to the end of study or deaths or willing to drop-out, whichever comes first, after signing the ICF. For the DMTs treated group, the date of patients initiating DMT will be defined as the index date. For patients not receiving DMTs, the date of enrollment into the registry will be the index date. Baseline data will be collected within 30 days prior to the index date. Diagram of data collection is provided in Figure 1.

Since this is a non-interventional study and does not impose a visit schedule, patients will be treated according to physician's decisions. Only data that are available during routine visits will be documented. Recommended data collection schedules are set up to reflect the most likely patterns of routine clinical care of patients with and without DMT. To ensure the excellent level of compliance on data collection, schedule points are set to follow clinical routine visits, most likely happen on day 0, 14, 28, 63 then every 4 months for patients treated with nusinersen, and at day 0, 90 (in children with SMA type 1 < 2 years of age), 180 then every 6 months for untreated patients or those on other DMT. Retrospective data will be extracted according to the schedule as well.

CONFIDENTIAL
The information contained herein may not be used, disclosed, or published without the written consent of Biogen

All data collected within the registry will be entered to the registry-specific e-forms by the responsible investigator, or a designated person, as timely as possible. Data entry and data corrections on e-forms are automatically tracked in the audit trail created by the EDC system. The EDC system and related IT infrastructure of this project will be based inside China.

Each patient will be assigned a unique but deidentified patient number when entering the registry, and the patient number will be used as the primary identifier throughout the entire participation in the study. All patients will be informed about the purpose of the patient number before enrollment.

The technical specifications of the database and the e-forms (variable names, attributes, and data entry checks) will be described in a corresponding database description plan. Prior to any data entry, the trial database and online edit checks of the e-forms will be tested and validated. Site data entry personnel will not have the access to the trial database until they have been trained and sign a data access agreement form. An audit trail records any changes of data (e.g. who and when makes the changes) will be developed.

The following data will be collected to evaluate the effectiveness of DMTs and the natural history of patients with SMA:

- Living status
- Clinical observations
- Scoliosis
- Motor function
- Wheelchair use
- Nutrition
- Pulmonary function
- Hospitalizations and comorbidities
- Motor measures
- Patient reported outcomes
- Electrophysiology and biomarkers

The following data will be collected to evaluate the safety profile of DMTs among patients on treatment:

- Adverse events (AEs) and serious adverse events (SAEs)

10. SAFETY DEFINITIONS, RECORDING, REPORTING, AND RESPONSIBILITIES

Throughout the course of the study, every effort must be made to remain alert to possible AEs. If an AE occurs, the first concern should be for the safety of the participant. If necessary, appropriate medical intervention should be provided.

Each participant or his/her legally authorized representative and/or main caregiver must be given the names and telephone numbers of site staff for reporting AEs, pregnancies, overdoses, and medical emergencies.

10.1. Definitions

10.1.1. Adverse Event

An AE is any untoward medical occurrence in a patient or clinical investigation participant administered a pharmaceutical product and that does not necessarily have a causal relationship with this treatment. An AE can therefore be any unfavorable and unintended sign (including an abnormal laboratory finding), symptom, or disease temporally associated with the use of a medicinal (investigational) product, whether or not related to the medicinal (investigational) product.

Determination of whether an abnormal assessment (e.g., laboratory value, vital sign result, and/or ECG) result meets the definition of an AE will be made by the Investigator. Abnormal results are not considered AEs unless one or more of the following criteria are met:

- The result meets the criteria for an SAE
- The result requires the participant to receive specific corrective therapy
- The result is considered by the Investigator to be clinically significant

10.1.2. Serious Adverse Event

An SAE is any untoward medical occurrence in a patient administered a pharmaceutical product at any dose:

- results in death
- in the view of the Investigator, places the participant at immediate risk of death (a life-threatening event); however, this does not include an event that, had it occurred in a more severe form, might have caused death
- requires inpatient hospitalization or prolongation of existing hospitalization
- results in persistent or significant disability/incapacity
- results in a congenital anomaly/birth defect.
- Is a medically important event

A medically important event is an AE that, in the opinion of the Investigator, may jeopardize the participant or may require intervention to prevent one of the other outcomes listed in the

definition above. (Examples of such medical events include allergic bronchospasm requiring intensive treatment in an emergency room or convulsions occurring at home that do not require an inpatient hospitalization.)

10.2. Safety Classifications

10.2.1. Investigator Assessment of Events

All events must be assessed to determine the following:

- If the event meets the criteria for an SAE as defined in Section 10.1.2.
- The relationship of the event to study treatment as defined in Section 10.2.2 .
- The severity of the event as defined in Section 10.2.3.

10.2.2. Relationship of Events to DMTs

The following definitions should be considered when evaluating the relationship of AEs and SAEs to DMTs:

**Relationship of Event to Commercial Drug**	
Not related	An AE will be considered “not related” to the use of DMTs if there is not a reasonable possibility that the event has been caused by it. Factors pointing toward this assessment include but are not limited to the lack of reasonable temporal relationship between administration of the product and the event, the presence of a biologically implausible relationship between the product and the AE (e.g., the event occurred before administration of the product), or the presence of a more likely alternative explanation for the AE.
Related	An AE will be considered “related” to the use of DMTs if there is a possibility that the event may have been caused by it. Factors that point toward this assessment include but are not limited to a positive rechallenge, a reasonable temporal sequence between administration of the product and the event, a known response pattern of the suspected product, improvement following discontinuation or dose reduction, a biologically plausible relationship between the product and the AE, or a lack of an alternative explanation for the AE.

10.2.3. Severity of Events

The following definitions should be considered when evaluating the severity of AEs and SAEs:

**Severity of Event**	
Mild	Symptom(s) barely noticeable to participant or does not make participant uncomfortable; does not influence performance or functioning; prescription drug not ordinarily needed for relief of symptom(s) but may be given because of personality of participant.
Moderate	Symptom(s) of a sufficient severity to make participant uncomfortable; performance of daily activity is influenced; participant is able to continue in study; treatment for symptom(s) may be needed.
Severe	Symptom(s) cause severe discomfort; symptoms cause incapacitation or significant impact on participant's daily life; severity may cause cessation of treatment with study treatment; treatment for symptom(s) may be given and/or participant hospitalized.

10.2.4. Expectedness of Events

The expectedness of all AEs for patients on nusinersen will be determined by The Sponsor according to the approved Prescribing Information.

10.3. Recording of Events

10.3.1. Adverse Events

All nonserious AEs (NSAEs) will be collected and recorded as part of this study.

Any NSAE that is to be collected as part of the study and that occurs between the time of first dose on the study and study completion or premature study withdrawal must be recorded on the CRF. Any of these AEs collected prospectively from participants who use nusinersen also should be recorded on a NSAE Form, which must be reported to the Sponsor within 30 days of the study site becoming aware of the event. Other AEs from participants who use other DMTs should notify the Investigator. AEs that are ongoing when the participant completes or discontinues the study will be followed by the Investigator until the event has resolved, stabilized, or returned to baseline status. Thereafter, the AE should be reported to the Sponsor only if the Investigator considers the AE to be related to nusinersen.

10.3.2. Serious Adverse Events

It is the Investigator's responsibility to ensure that the SAE reporting information and procedures are used and followed appropriately.

Any SAE experienced by a participant after the participant signs the ICF and before study completion or premature study withdrawal is to be recorded on the CRF, regardless of the event relationship to DMTs. The site must formally notify the Sponsor to record in PV database within 24 hours of becoming aware of the SAEs from participants who use nusinersen by submitting the SAE Form. Thereafter, the event should be reported to the Sponsor on an SAE Form only if the Investigator considers the SAE to be related to nusinersen.

CONFIDENTIAL
The information contained herein may not be used, disclosed, or published without the written consent of Biogen

Any SAE experienced by a participant that is discovered during secondary use of data should not be recorded on an SAE Form. This information should be provided only on the CRF.

Any SAE that is ongoing when the participant completes or discontinues the study will be followed by the Investigator until the event has resolved, stabilized, or returned to baseline status.

10.3.2.1. Deaths

Death is an outcome of an event. The event that resulted in death should be recorded and reported as an SAE within 24 hours of the site becoming aware of the event. The Investigator should make every effort to obtain and send death certificates and autopsy reports to the Sponsor. The term death should be reported as an SAE only if the cause of death is not known and cannot be determined.

10.3.3. Reporting Events

Reporting Information for SAEs and NSAEs involving Nusinersen Collected Prospectively
To report initial or follow-up information on an NSAE or SAE forms, e-mail a completed NSAE or SAE form to PVChina@Biogen.com; refer to the Study Reference Guide's Official Study Contact List for complete contact information.

10.4. Procedures for Handling Special Situations

10.4.1. Overdose

An overdose is any dose of DMTs given to a participant or taken by a participant that exceeds the dose described in the local label. Overdoses are not considered AEs; however, all overdoses should be recorded on an Overdose Form from participants who use nusinersen and e-mailed to the Sponsor within 24 hours. An overdose should be reported even if it does not result in an AE. If an overdose results in an SAE, both the SAE and Overdose forms must be completed and e-mailed to the Sponsor.

10.4.2. Reporting Pregnancy

The Investigator should refer to the approved local label for guidance if female participants become pregnant or are considering becoming pregnant during the study.

At each routine visit, female participants of childbearing potential will be asked about their pregnancy status and possible pregnancies/spontaneous abortions since the last visit or contact. Spontaneous abortions are considered to be SAEs and must be reported as such.

Congenital abnormalities/birth defects in the offspring of male or female participants should be reported if conception occurred after administration of DMTs.

10.4.3. Coordination With the Pregnancy Registry

Not applicable.

10.5. Contraception

For participants on DMTs, refer to the approved Prescribing Information for information related to contraception.

10.6. Medical Emergency

In a medical emergency requiring immediate attention, site staff will apply appropriate medical intervention, according to current standards of care

10.7. Investigator Responsibilities

10.7.1. Investigator

The Investigator's responsibilities include the following:

- Review all AEs to determine seriousness and fulfillment of collection criteria defined in Section 10.2.
- Monitor and record all SAEs (and AEs defined in Section 10.3), regardless of the relationship to DMTs.
- Determine the relationship of each SAE and AE defined in Section 10.3 to DMTs.
- Determine the onset and resolution dates of each SAE and AE defined in Section 10.3.
- Record all pregnancies
- Complete the appropriate form for each SAE, AE, overdose, and pregnancy for patients on nusinersen and email it to the Sponsor within 24 hours/30 days of the site staff becoming aware of the event.
- Pursue SAE follow-up information actively and persistently. Follow-up information must be reported to the Sponsor within 24 hours of the site staff becoming aware of new information for patients on nusinersen.
- Ensure all SAE reports are supported by documentation in the participants' medical records.
- Report SAEs (and AEs defined in Section 10.3) to local ethics committees, as required by local law.

10.7.2. Biogen's Responsibilities

Biogen's responsibilities include the following:

- Before site activation and participant enrollment, Biogen or designee is responsible for reviewing with site staff the definition of an SAE, as well as the instructions for monitoring, recording, and reporting SAEs (and AEs defined in Section 10.3).

- Determine the expectedness of all SAEs/AEs for participants on nusinersen.
- Notify all appropriate regulatory authorities, central ethics committees, and Investigators of safety reports as required by local law, within required time frames.

CONFIDENTIAL
The information contained herein may not be used, disclosed, or published without the written consent of Biogen

11. STATISTICAL CONSIDERATIONS

The objectives of the study and the endpoints to be analyzed are listed in Section 4.

11.1. General Considerations

Analysis details will be pre-specified in Statistical Analysis Plans (SAP). All statistical programming for analysis will be performed with the Statistical Analysis System (SAS)/R software.

11.2. Analysis Sets

Analyses will be performed for all patients and can be repeated for subgroups of patients according to specific research questions, if appropriate. Analyses referring to special research questions can be conducted, if requested and will be described in an SAP.

11.3. Methods of Analysis

The statistical analysis to assess the primary endpoints will be mostly descriptive. Data quality will be assessed by describing recruitment figures and data completeness (percentages of missing values). Patient characteristics will be displayed in terms of demographic data and disease characteristics at first diagnosis. Time–to-event endpoints will be estimated and displayed using the Kaplan-Meier method. Continuous data will be summarized by arithmetic mean, standard deviation, minimum, 25% quantile, median, 75% quantile, maximum, and the number of complete and missing observations. If appropriate, continuous variables can also be presented in categories. Categorical data will be summarized by the total number of patients in each category and the number of missing values. Relative frequencies are displayed as valid % (number of patients divided by the number of patients with non-missing values). Details on statistical methods and potential subgroup analyses will be included in an SAP.

11.4. Interim Analyses

The enrollment of first patient (FPFV) is scheduled at Q3 2021. There will be four interim analyses in total, which will be conducted yearly per the statistical analysis plan after the first patient enrollment. The recruitment will target as many patients as possible, but the actual number of patients enrolled is subject to operational speed at each site.

11.5. Sample Size Justification

No formal sample size calculation is performed, as this registry aims at collecting SMA patient's data as completely as possible by enrolling all eligible SMA patients. It is expected that overall approximately 600 patients will be included in the registry.

CONFIDENTIAL
The information contained herein may not be used, disclosed, or published without the written consent of Biogen

12. ETHICAL REQUIREMENTS

The Sponsor, any contracted third party, and the Investigators must comply with all instructions, regulations, and agreements in this protocol and applicable International Council for Harmonisation (ICH) and Good Pharmacoepidemiology Practice (GPP) guidelines and conduct the study according to local regulations.

The Investigator may delegate responsibilities for study-related tasks where appropriate to individuals sufficiently qualified by education, training, and experience, in accordance with applicable ICH and GPP guidelines. The Investigator should maintain a list of the appropriately qualified persons to whom significant study-related duties have been delegated. The Investigator is responsible f or supervising those individuals and for implementing procedures to ensure the integrity of the tasks performed and any data generated.

The Investigators are responsible for demonstrating timely oversight of all clinical trial data from their site, including data external to the electronic data capture system, such as laboratory, imaging, and electronic clinical outcomes assessment data. Investigators must approve all their data on completed CRFs by signing electronically, at the participant, visit, or casebook level, at any time prior to an interim lock or database lock, as well as before any subsequent re-lock. The electronic data capture system does not prohibit Investigator approval or signing in any way.

12.1. Declaration of Helsinki

This study will be performed in alignment with the ethical principles outlined in the Declaration of Helsinki.

12.2. Ethics Committee

Investigators must obtain ethics committee approval of the protocol, ICF, and other required study documents prior to starting the study. The Sponsor will submit documents on behalf of the study sites in countries other than the United States.

If the Investigator makes any changes to the ICF, the Sponsor must approve the changes before the ICF is submitted to the ethics committee. A copy of the approved ICF must be provided to the Sponsor. After approval, the ICF must not be altered without the agreement of the relevant ethics committee and the Sponsor.

It is the responsibility of the Investigators to ensure that all aspects of institutional review are conducted in accordance with current applicable regulations.

The Sponsor must receive a letter documenting ethics committee approval, which specifically identifies the protocol, protocol number, and ICF, prior to the initiation of the study. Protocol amendments will be participant to the same requirements as the original protocol.

A progress report must be submitted to the ethics committee at required intervals and not less than annually.

At the completion or termination of the study, where required, the study site must submit a close-out letter to the ethics committee and the Sponsor.

12.3. Changes to Final Study Protocol

All protocol amendments must be submitted to the ethics committee and regulatory authorities if required by local law. Protocol modifications that affect participant safety, the scope of the investigation, or the scientific quality of the study must be approved by the ethics committee before implementation of such modifications to the conduct of the study. If required by local law, such modifications must also be approved by the appropriate regulatory agency prior to implementation.

However, the Sponsor may, at any time, amend this protocol to eliminate an apparent immediate hazard to a participant. In this case, the appropriate regulatory authorities will be notified subsequent to the modification.

In the event of a protocol modification, the ICF may require similar modifications (see Section 12.4).

12.4. Participant Information and Consent

Prior to any data collection under this protocol, informed consent with the approved ICF must be obtained in accordance with local practice and regulations.

The background of the proposed study, the procedures, the benefits and risks of the study, and that study participation is voluntary for the participant must be explained to the participant (or the participant's legally authorized representative). The participant must be given sufficient time to consider whether to participate in the study.

In addition, participants who have the capacity should provide their assent to participate in the study. The level of information provided to participants should match their level of understanding as determined by the Investigator and in accordance with applicable regulations and guidelines.

A copy of the signed and dated ICF and assent must be given to the participant and/or the participant's legally authorized representative. The original signed and dated ICF will be retained with the study records. Local regulations must be complied with in respect to the final disposition of the original and copies of the signed and dated ICFs.

Confirmation of informed consent and assent must also be documented in the participant's medical record.

12.5. Participant Data Protection

Prior to any data collection under this protocol, candidates must also provide all authorizations required by local law (e.g., Protected Health Information authorization in North America).

During the study, participants' full date of birth for pediatric studies will be collected (unless the collection is not permitted by applicable law or not approved by the governing ethics committee). These data will be used in the analysis of the safety and/or pharmacokinetic profile of the study treatment. It is unknown whether the effects of the study treatment are influenced by race or ethnicity.

Study reports will be used for research purposes only. The participant will not be identified by name in CRFs, study-related forms, study reports, or any related publications. The Sponsor, its

partners and designees, ethics committees, and various government health agencies may inspect the records of this study. Every effort will be made to keep the participant's personal medical data confidential.

12.6. Compensation for Injury

The Sponsor maintains appropriate insurance coverage for clinical trials and will follow applicable local compensation laws.

12.7. Conflict of Interest

The Investigators should address any potential conflicts of interest (e.g., financial interest in the Sponsor) or its partnering company with the participant before the participant makes a decision to participate in the study.

12.8. Study Report Signatory

The Sponsor will designate one or more of the participating Investigators as a signatory for the study report. This determination will be made by several factors, including but not limited to, the Investigator's experience and reputation in the studied indication; the Investigator's contribution to the study in terms of design, management, and/or enrollment; or by other factors determined to be relevant by the Sponsor.

The Sponsor will follow all applicable local regulations pertaining to study report signatories.

12.9. Registration of Study and Disclosure of Study Results

The Sponsor will register the study and post study results regardless of the outcome on a publicly accessible website in accordance with the applicable laws and regulations.

The Sponsor also will notify, when required, the regulatory authorities and ethics committees about the completion or termination of this study and send a copy of the study synopsis in accordance with necessary timelines.

12.10. Retention of Study Data

The minimum retention time for study records will meet the strictest standard applicable to that site, as dictated by any institutional requirements or local, national, or regional laws or regulations. Prior to proceeding with destruction of records, the Investigator must notify the Sponsor in writing and receive written authorization from the Sponsor to destroy study records. In addition, the Investigator must notify the Sponsor of any changes in the archival arrangements including, but not limited to archival at an offsite facility or transfer of ownership if the Investigator leaves the site.

CONFIDENTIAL
The information contained herein may not be used, disclosed, or published without the written consent of Biogen

13. KEY ROLES AND STUDY GOVERNANCE COMMITTEES

13.1. Vendors

The Sponsor will ensure oversight of any study-related duties and functions carried out on its behalf and will specify in writing all duties and functions that are transferred.

13.1.1. Contract Research Organization

A CRO will be responsible for administrative aspects of the study including, but not limited to, study initiation, monitoring, and management of SAE reports and data management. Before patients are screened at each study site, the CRO will review study responsibilities with the Investigators and other study site staff, as appropriate.

13.1.2. Electronic or Remote Data Capture

Participant information will be captured and managed by study sites on electronic CRFs by a Web-based electronic data capture tool configured by the CRO and hosted by IQVIA.

Electronic Clinical Outcome Assessment will be entered by participant, caregiver, rater, site staff on a handheld device, Web-based tool. Site staff will monitor data via a secure Web portal.

14. ADMINISTRATIVE PROCEDURES

14.1. Study Site Initiation

The Investigator must not enroll any participants in this study prior to the Sponsor or designee completing a study initiation visit. This initiation visit with the Investigator and other site staff, as appropriate, will include a detailed review of the protocol, study procedures, and study responsibilities.

14.2. Quality Control and Assurance

Quality control procedures will be implemented at each stage of data handling to ensure that all data are reliable and have been processed correctly. Data anomalies will be communicated to the sites for clarification and resolution, as appropriate. The Investigator is responsible for endorsing all CRF data prior to any interim or final database lock.

During and/or after completion of the study, quality assurance officers named by the Sponsor or the regulatory authorities may wish to perform onsite audits or inspections. The Investigator will be expected to cooperate with any audit or inspections and to provide assistance and documentation (including source data) as requested.

Designated personnel will provide the data to sponsor, encoded in the designed CRF and confirm on the data quality (completeness and accuracy) and integrity. All data analysis programs will be written by designated CRO with its respective quality control (QC) program. To ensure proper knowledge of the study background and objectives in order to provide input in study design and data analysis, all programmers will be involved in all discussions regarding the analyses.

Designated CRO is responsible for implementing and maintaining quality assurance and quality control systems with written SOPs to ensure that data are generated, documented (recorded), and reported in compliance with the protocol and the applicable regulatory requirement(s).

Designated CRO will assure database quality including review the data entered into the CRFs by investigational staff for completeness, consistency, and plausibility, and in accordance with a pre-specified data validation plan. After running the check programs, the resulting query lists will be sent to the investigator for correction or verification of the documented data. Data corrections will be entered directly into the electronic data collection platform by the responsible investigator, or designated person.

All programs which can be used to influence data or data quality will be validated (e.g. data validation programs, programs for CRF/query tracking, programs for import of data into SAS or for import of external data, etc.).

In all scenarios, the physician must maintain source documents for each patient in the study, consisting of case and visit notes (hospital or clinic medical records) containing demographic and medical information, and the results of any other tests or assessments. All information entered in the CRF must be traceable to these source documents in the patient's file.

14.3. Monitoring of the Study

The Sponsor or its designee may conduct onsite visits at the study facilities for the purpose of monitoring various aspects of the study. The Investigator must agree to Sponsor-authorized

personnel having direct access to participant (or associated) files for the purpose of verifying entries made in the CRF, and assist with their activities, if requested. Adequate space and time for monitoring visits should be made available by the Investigator or site staff. The site must complete the CRFs in a timely manner and on an ongoing basis to allow regular review by the study team.

A clinical site monitoring plan will detail who performs the monitoring, how often, and the extent of review. It also will provide the monitoring strategy, with emphasis on participant safety, data integrity, and critical data and processes.

14.4. Study Funding

Biogen is the Sponsor of the study and is funding the study. All financial details are provided in the separate contract(s) between the institution, Investigator, and Biogen.

14.5. Publications

Details are included in the Clinical Trial Agreement for this study.

CONFIDENTIAL
The information contained herein may not be used, disclosed, or published without the written consent of Biogen

15. REFERENCES

1. Pearn J. Incidence, prevalence, and gene frequency studies of chronic childhood spinal muscular atrophy. Journal of medical genetics. 1978;15(6):409-413
2. Prior TW, Snyder PJ, Rink BD, et al. Newborn and carrier screening for spinal muscular atrophy. American journal of medical genetics. Part A. 2010;152A(7):1608-1616
3. Oskoui M, Levy G, Garland CJ, et al. The changing natural history of spinal muscular atrophy type 1. Neurology. 2007;69(20):1931-1936
4. RS Finkel, et al, ENMC SMA Workshop Study Group. 218th ENMC International Workshop:: Revisiting the consensus on standards of care in SMA Naarden, The Netherlands, 19-21 February 2016/ Neuromuscul Disord. 2017 Jun;27(6):596-605Pechmann A, Kirschner J. Diagnosis and New Treatment Avenues in Spinal Muscular Atrophy. Neuropediatrics. 2017;48(4):273-281
5. Verhaart, I.E.C., Robertson, A., Leary, R. et al. A multi-source approach to determine SMA incidence and research ready population. Journal of Neurology. 2017;264(7):1465–1473
6. Bladen CL, Thompson R, Jackson JM, et al. Mapping the differences in care for 5,000 spinal muscular atrophy patients, a survey of 24 national registries in North America, Australasia and Europe. Journal of neurology. 2014;261(1):152-163

16. SIGNED AGREEMENT OF THE STUDY PROTOCOL

I have read the foregoing protocol, "Pediatric Patients with Spinal Muscular Atrophy in China: A Nationwide Registry," and agree to conduct the study according to the protocol and the applicable ICH guidelines and GPP and local regulations, and to inform all who assist me in the conduct of this study of their responsibilities and obligations.

____

Investigator's Signature Date

____

Investigator's Name (Print)

____

Study Site (Print)

CONFIDENTIAL
The information contained herein may not be used, disclosed, or published without the written consent of Biogen
