# Supplementary material for: Nusinersen effectiveness and safety in pediatric patients with 5q-spinal muscular atrophy: a multi-center disease registry in China
Source: J Neurol. 2024 Jul 2;271(8):5378–91. doi: 10.1007/s00415-024-12442-w (PMC11319379; doi:10.1007/s00415-024-12442-w)

'Patient profile of the change of patients gaining or losing WHO motor milestone assessed by Sitting without support over time for SMA Type 1'

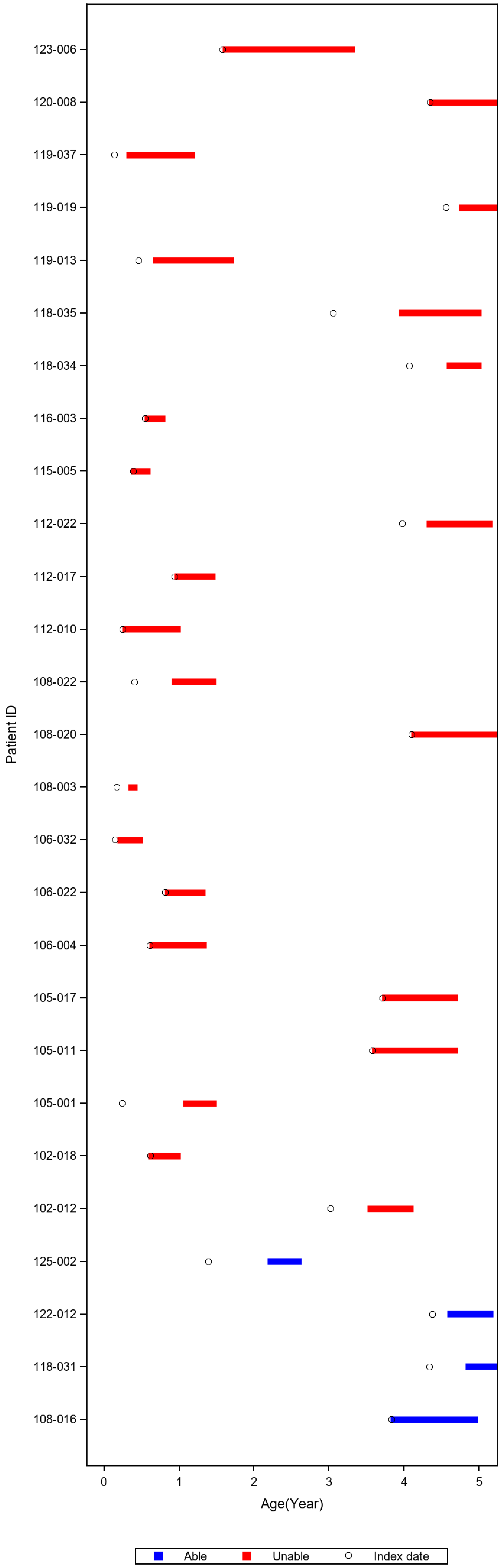

'Patient profile of the change of patients gaining or losing WHO motor milestone assessed by Hands-and-knees crawling over time for SMA Type 1'

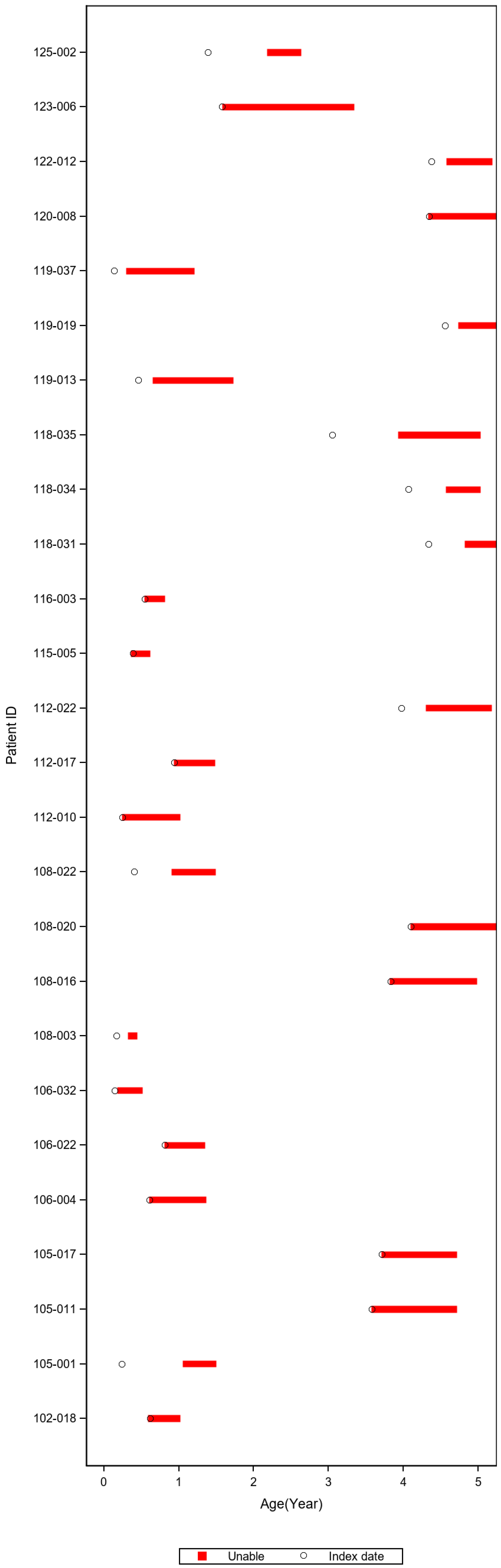

'Patient profile of the change of patients gaining or losing WHO motor milestone assessed by Standing with assistance over time for SMA Type 1'

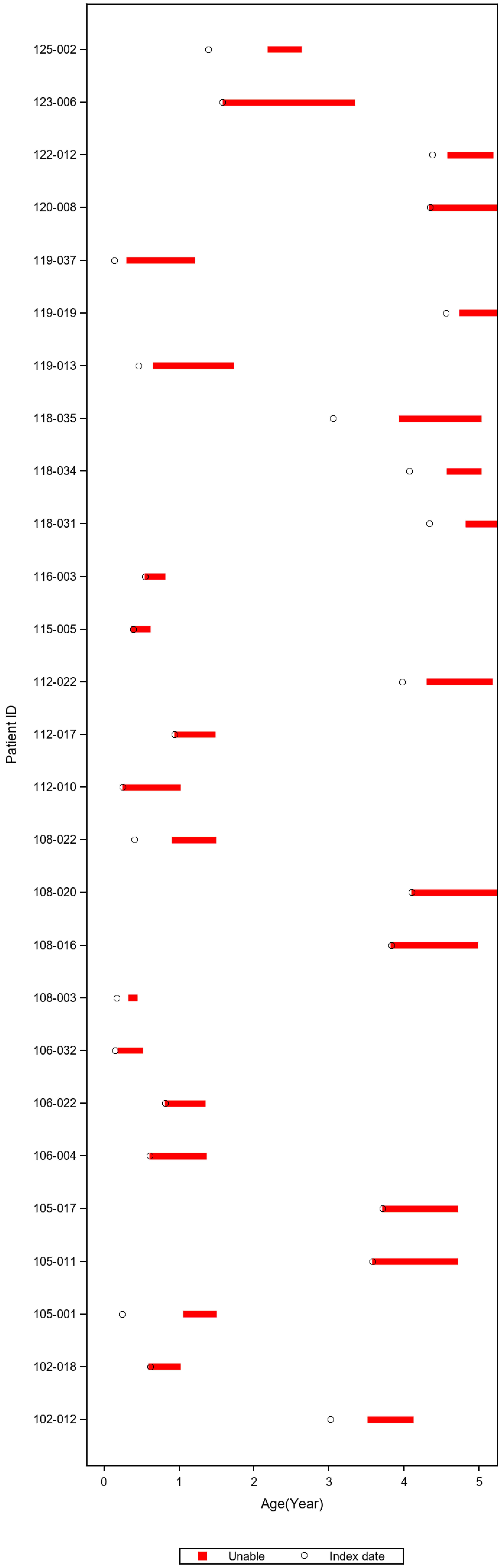

'Patient profile of the change of patients gaining or losing WHO motor milestone assessed by Walking with assistance over time for SMA Type 1'

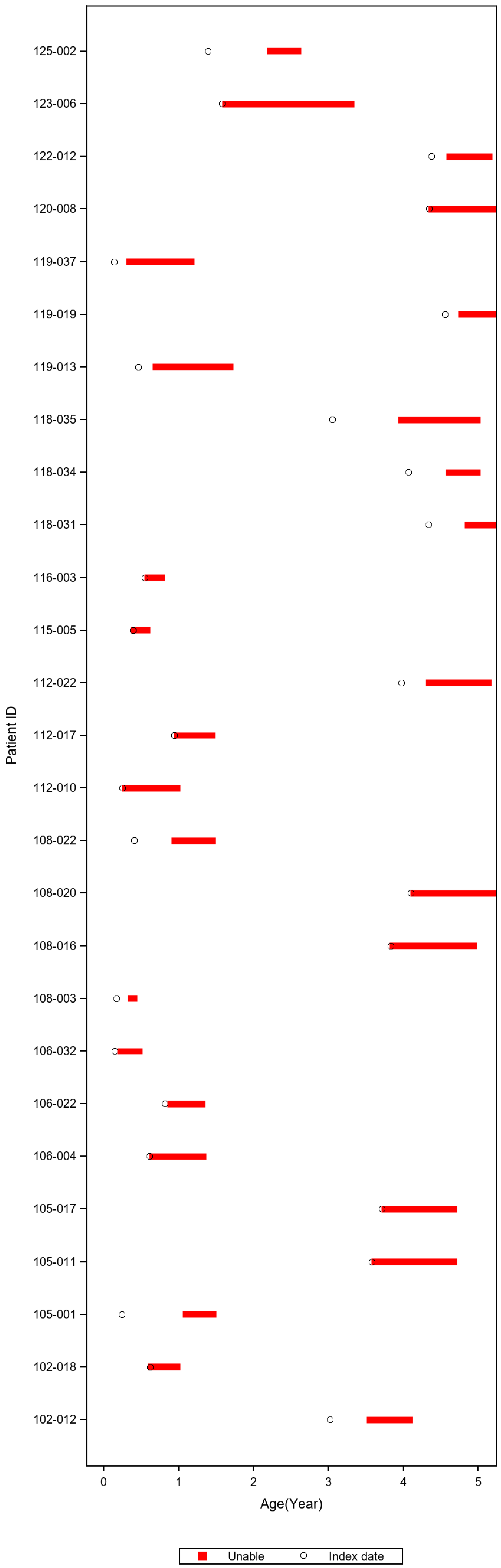

'Patient profile of the change of patients gaining or losing WHO motor milestone assessed by Standing alone over time for SMA Type 1'

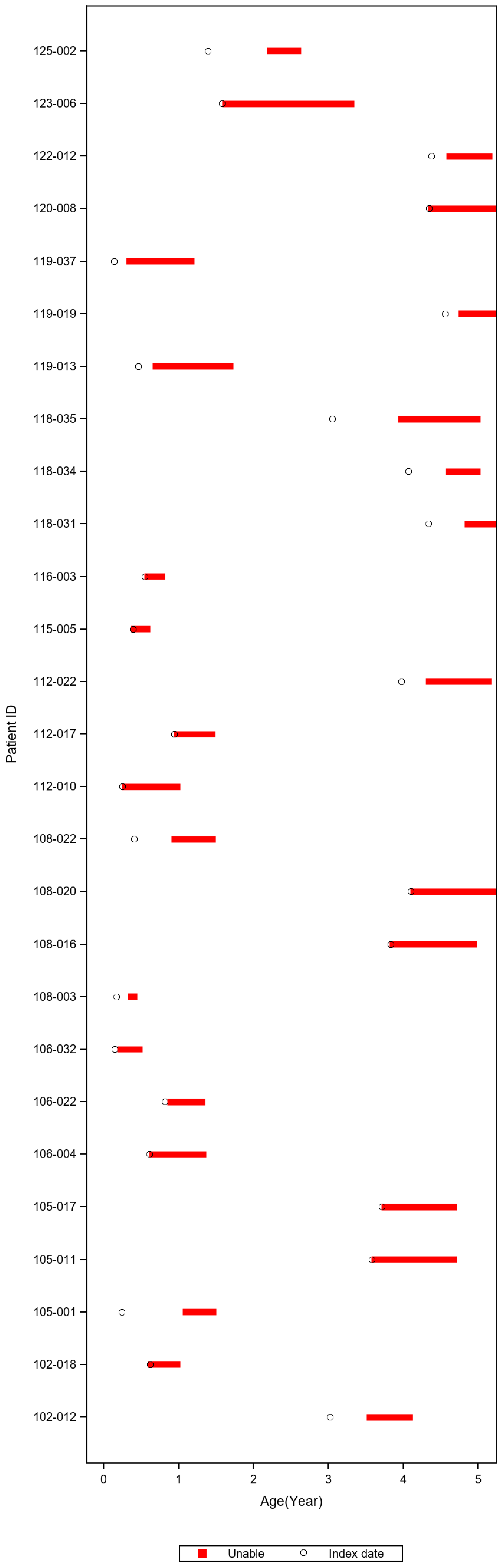

'Patient profile of the change of patients gaining or losing WHO motor milestone assessed by Walking alone over time for SMA Type 1'

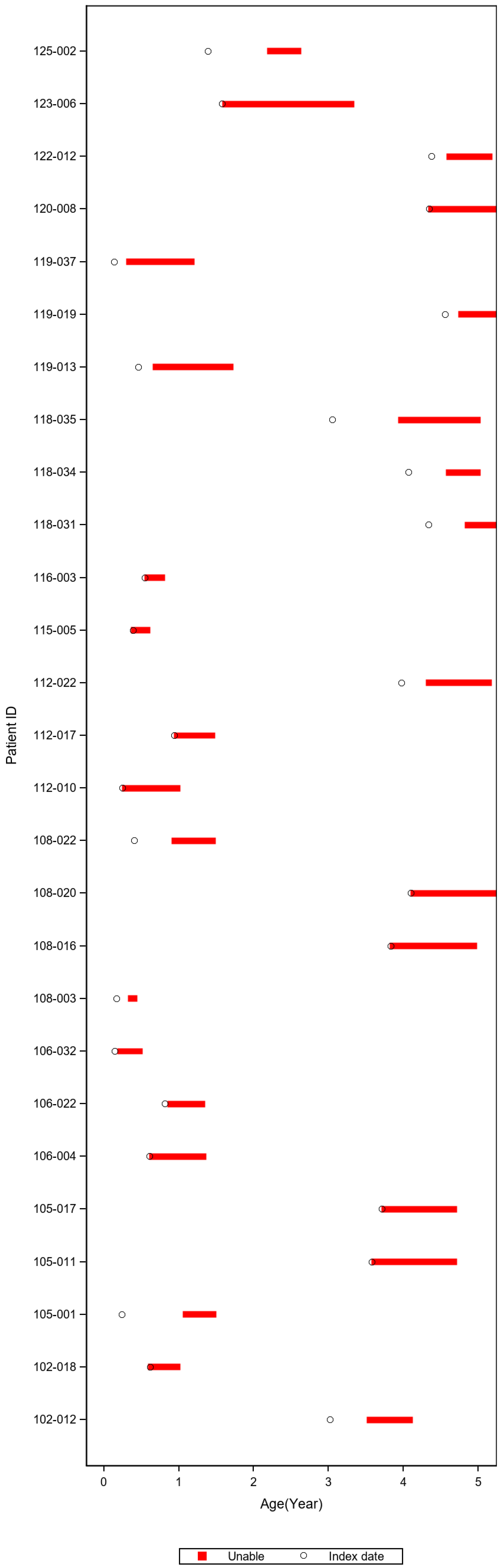

'Patient profile of the change of patients gaining or losing WHO motor milestone assessed by Sitting without support over time for SMA Type 2'

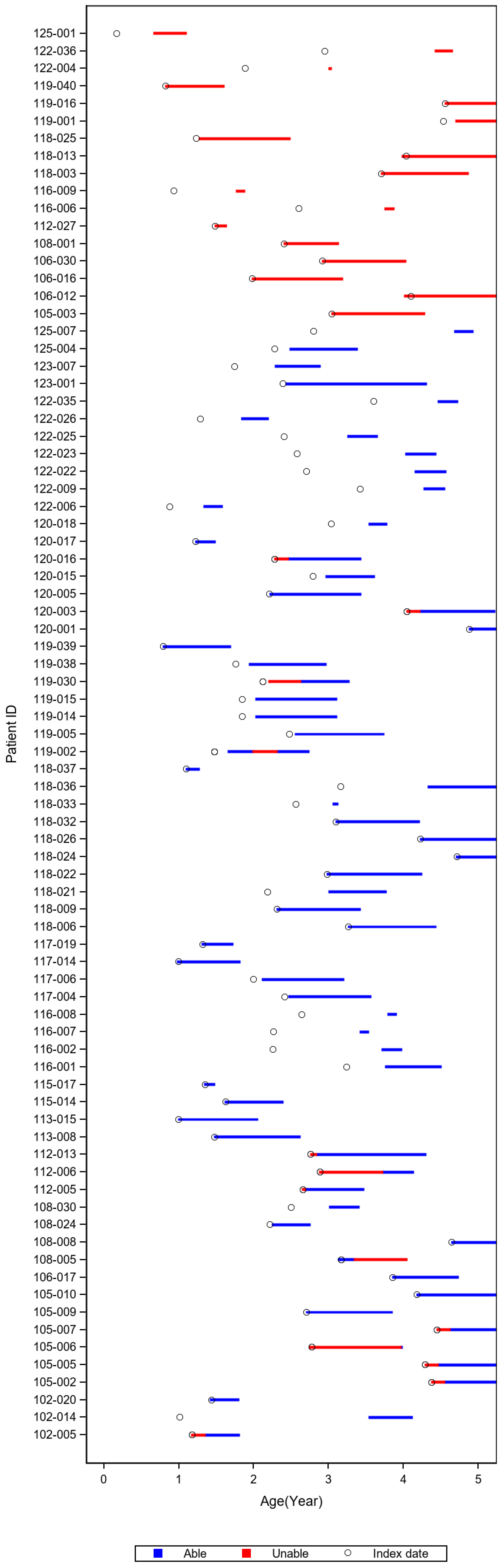

'Patient profile of the change of patients gaining or losing WHO motor milestone assessed by Hands-and-knees crawling over time for SMA Type 2'

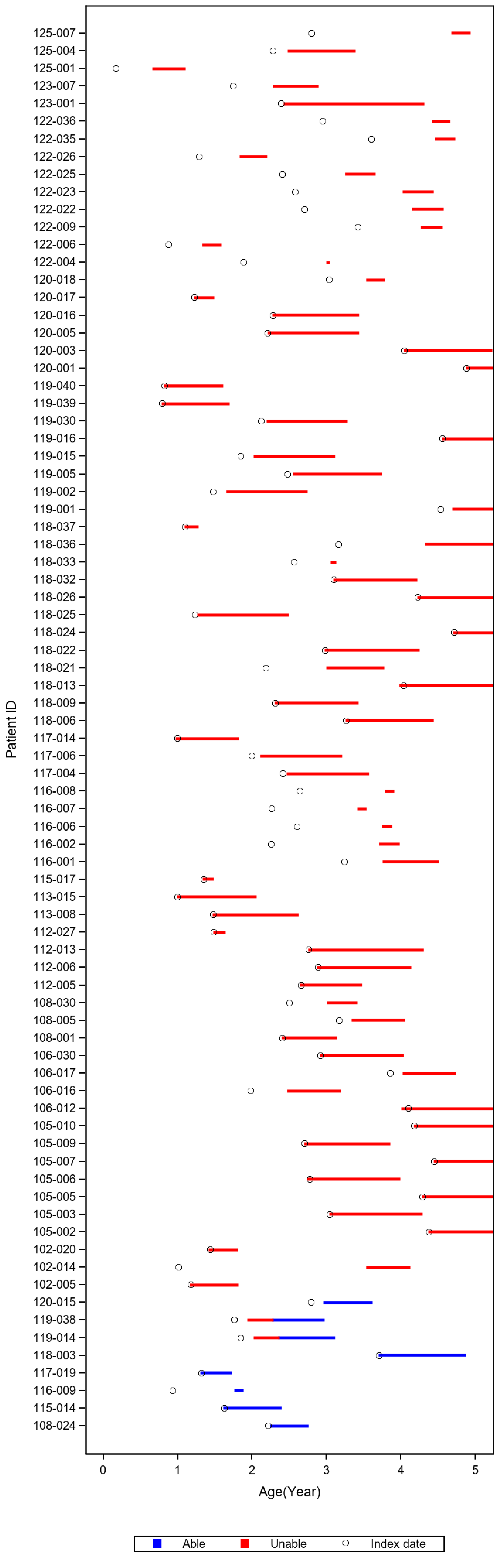

'Patient profile of the change of patients gaining or losing WHO motor milestone assessed by Standing with assistance over time for SMA Type 2'

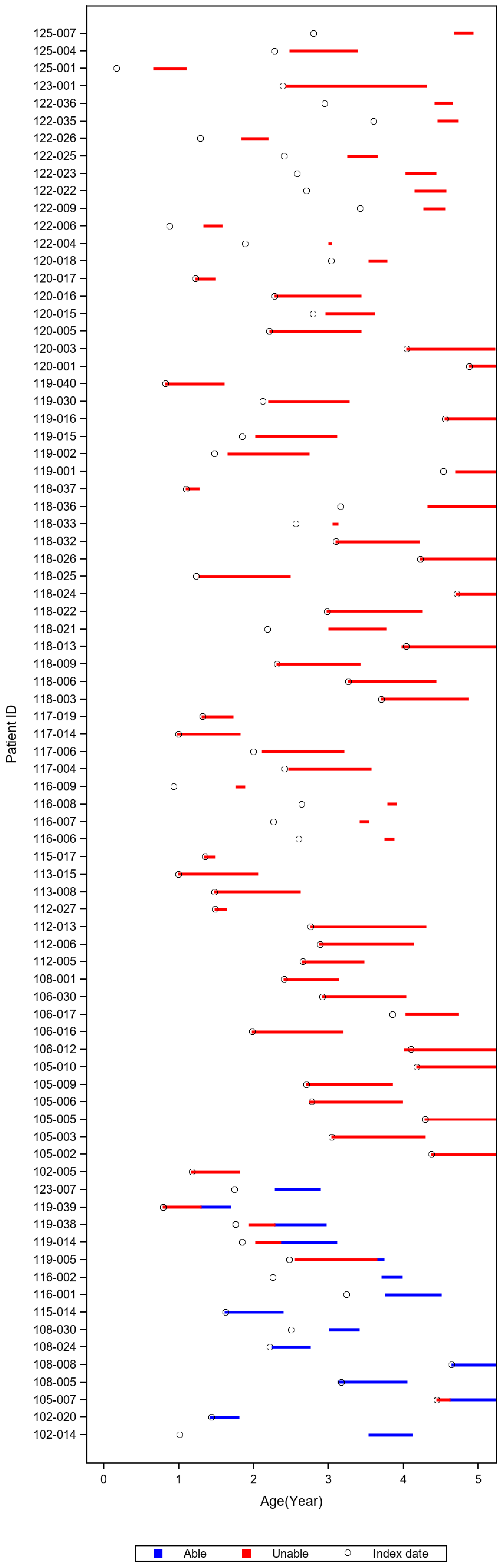

'Patient profile of the change of patients gaining or losing WHO motor milestone assessed by Walking with assistance over time for SMA Type 2'

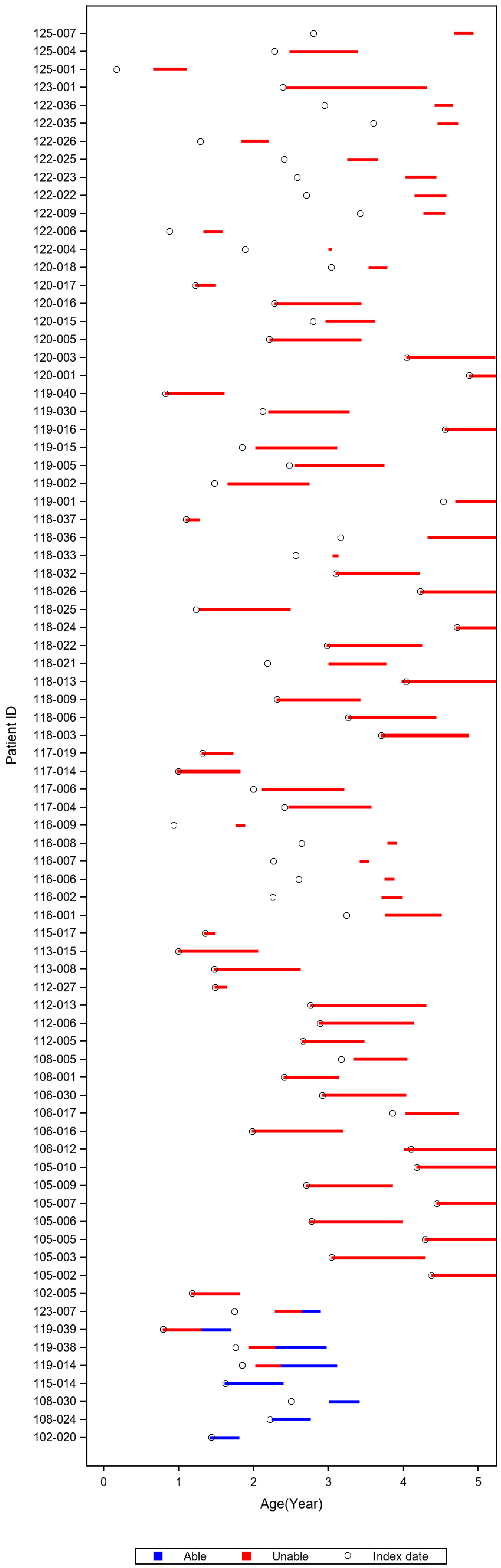

'Patient profile of the change of patients gaining or losing WHO motor milestone assessed by Standing alone over time for SMA Type 2'

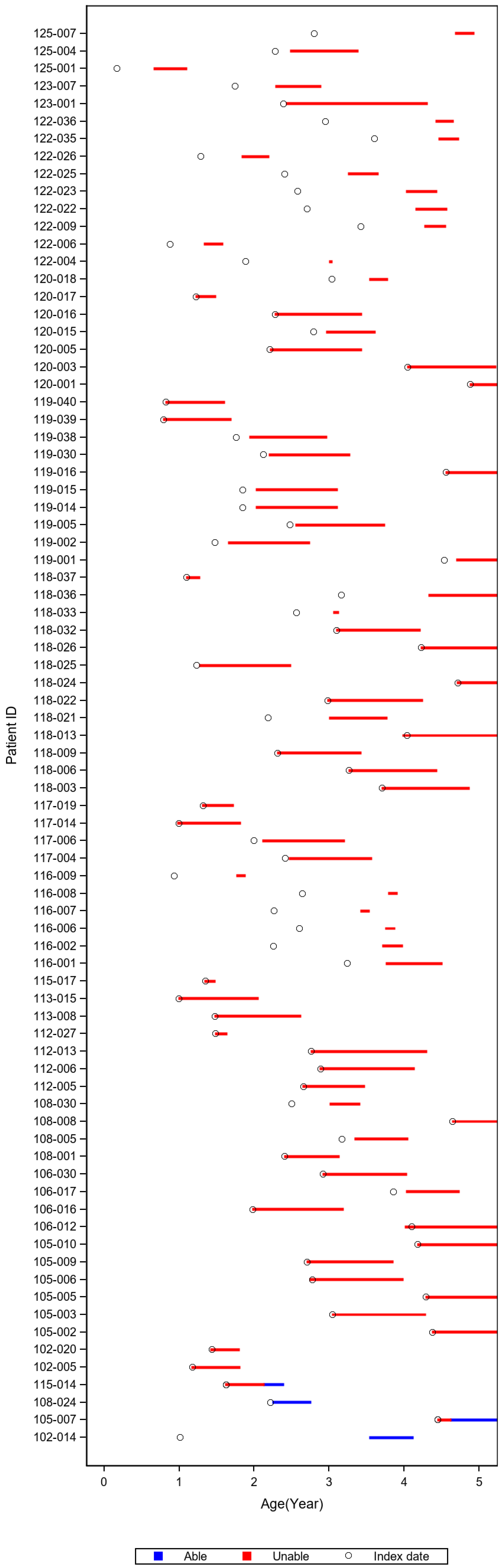

'Patient profile of the change of patients gaining or losing WHO motor milestone assessed by Walking alone over time for SMA Type 2'

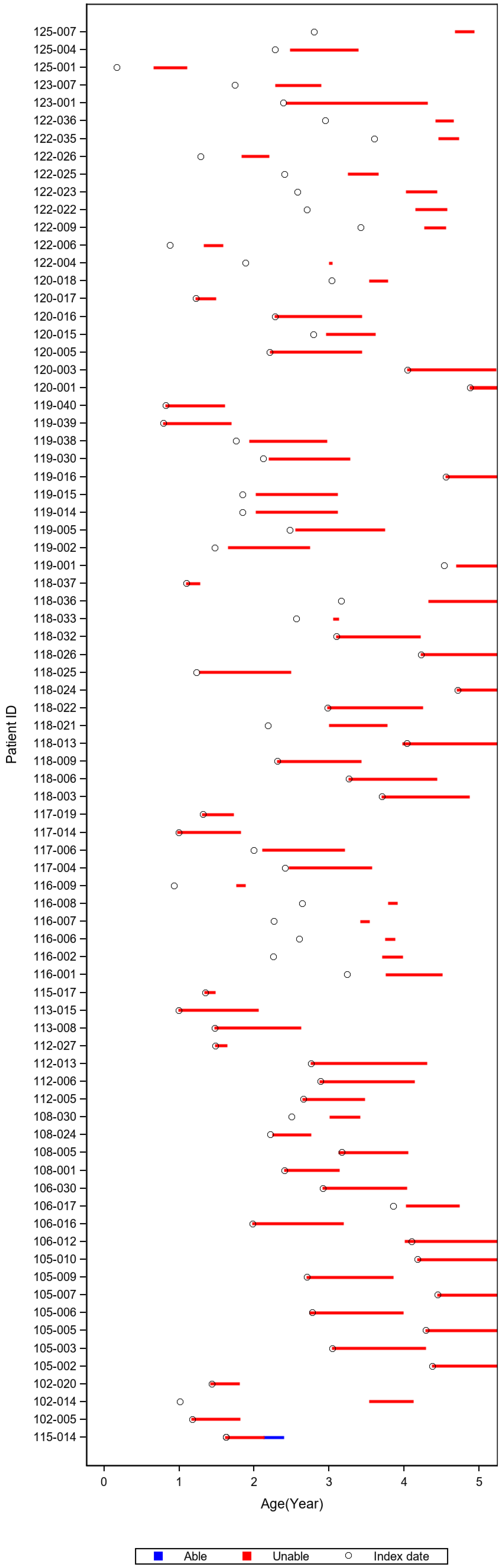

'Patient profile of the change of patients gaining or losing WHO motor milestone assessed by Sitting without support over time for SMA Type 3'

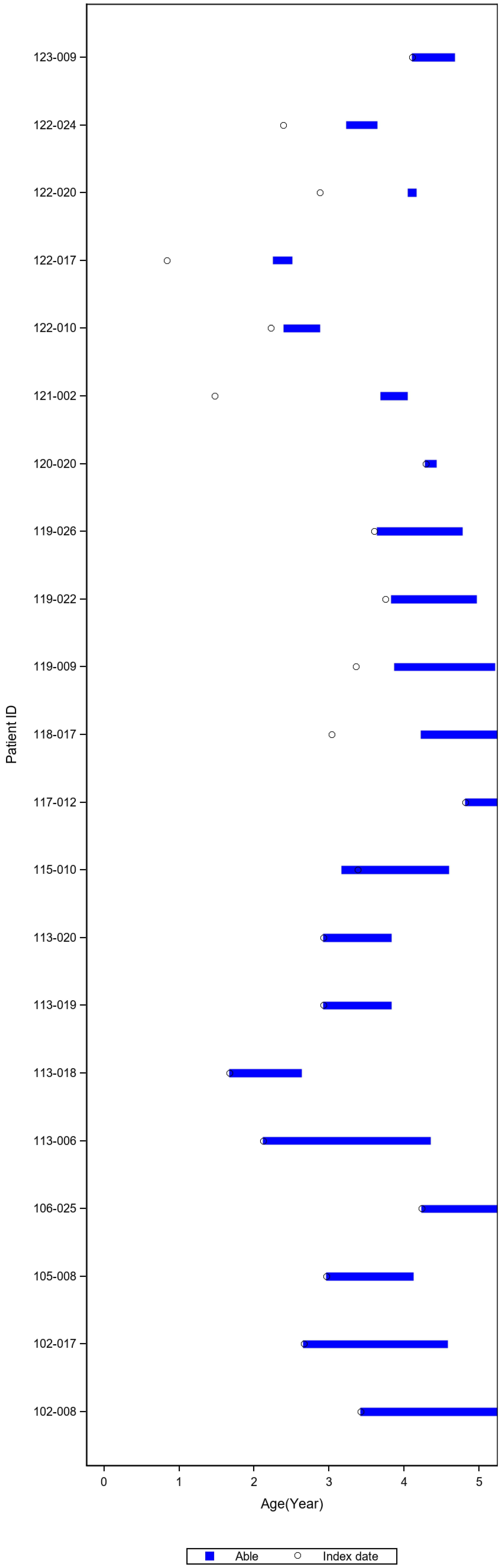

'Patient profile of the change of patients gaining or losing WHO motor milestone assessed by Hands-and-knees crawling over time for SMA Type 3'

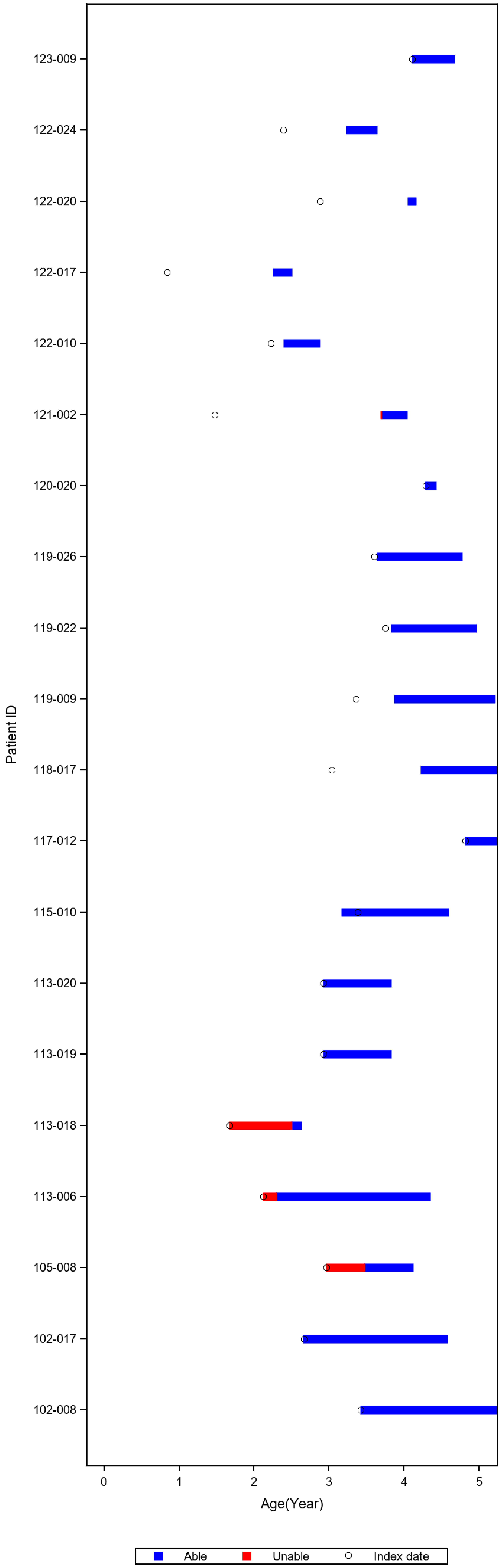

'Patient profile of the change of patients gaining or losing WHO motor milestone assessed by Standing with assistance over time for SMA Type 3'

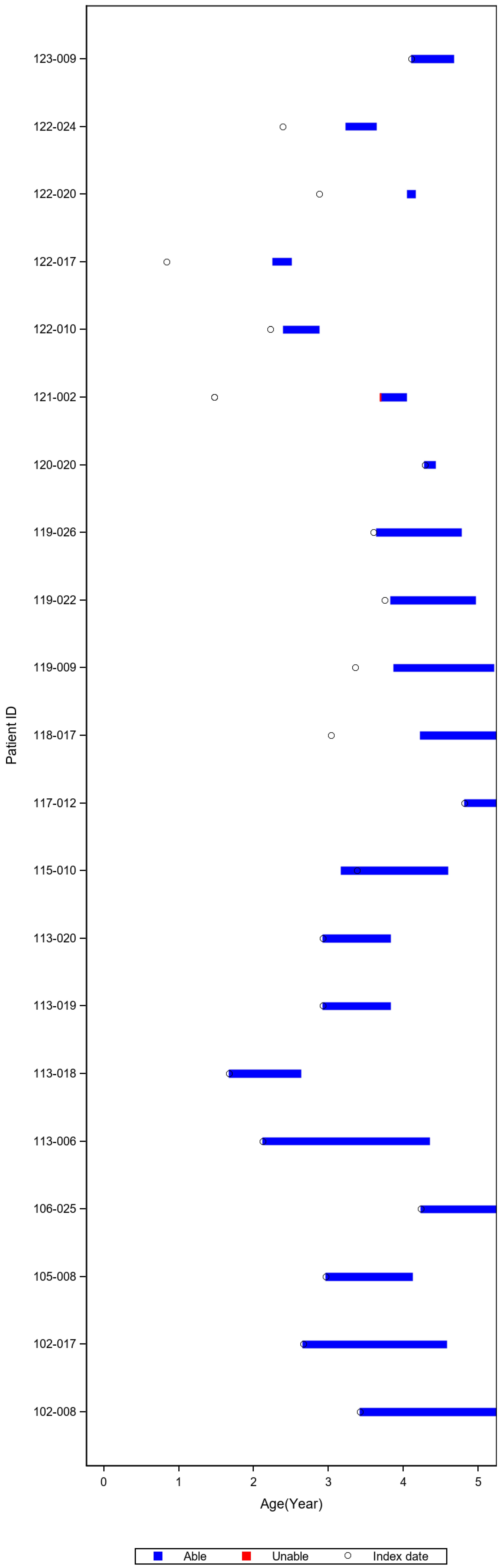

'Patient profile of the change of patients gaining or losing WHO motor milestone assessed by Walking with assistance over time for SMA Type 3'

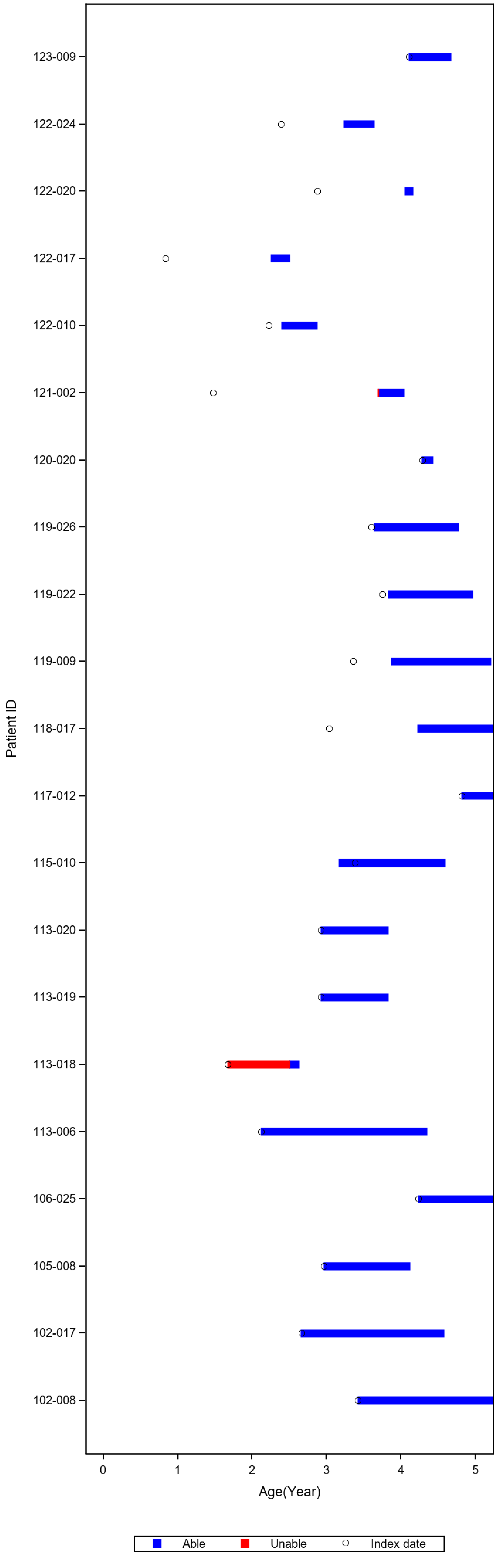

'Patient profile of the change of patients gaining or losing WHO motor milestone assessed by Standing alone over time for SMA Type 3'

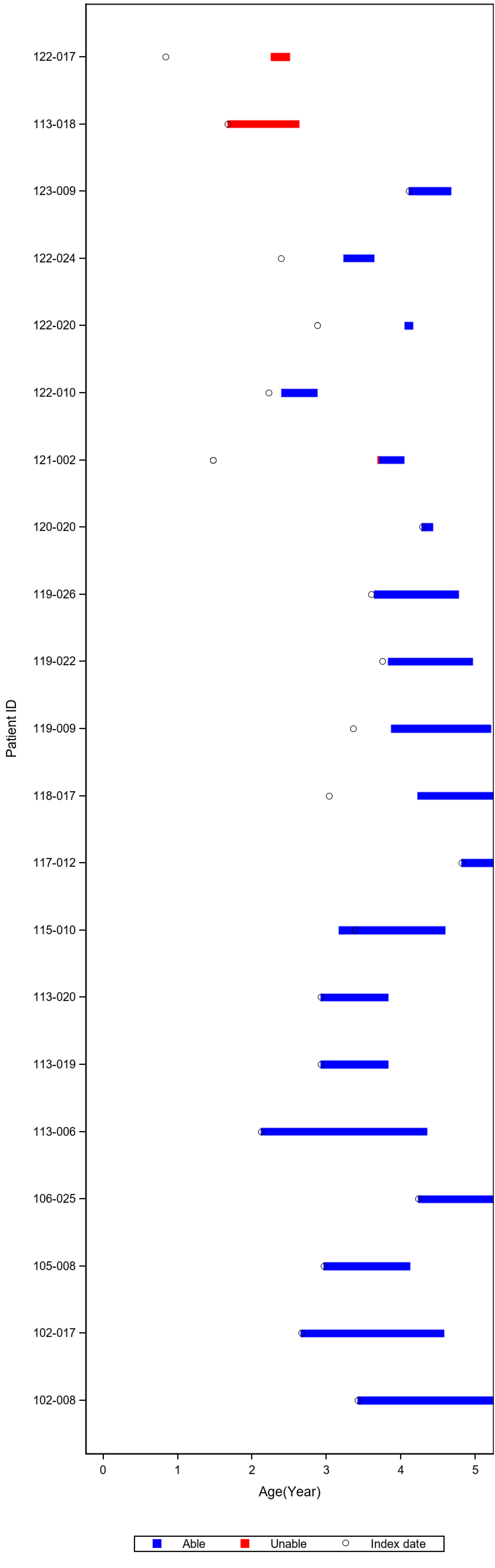

'Patient profile of the change of patients gaining or losing WHO motor milestone assessed by Walking alone over time for SMA Type 3'

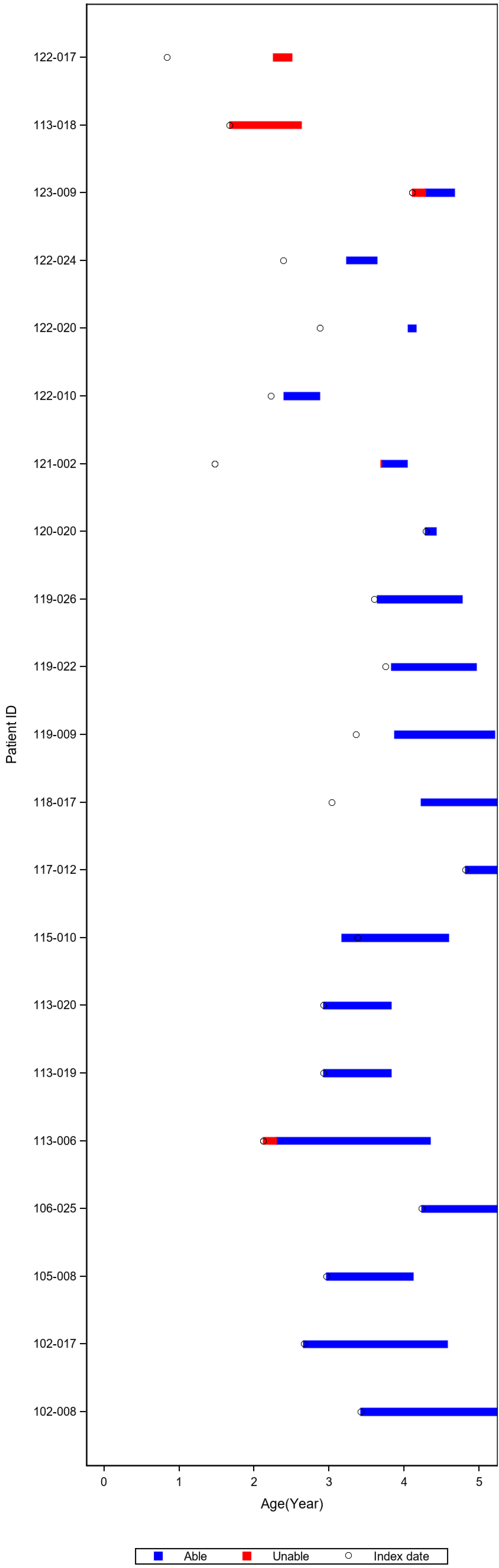

'Patient profile of the change of proportion of patients gaining Motor Function assessed by Holding Head Up without Support over time for SMA Type 1'

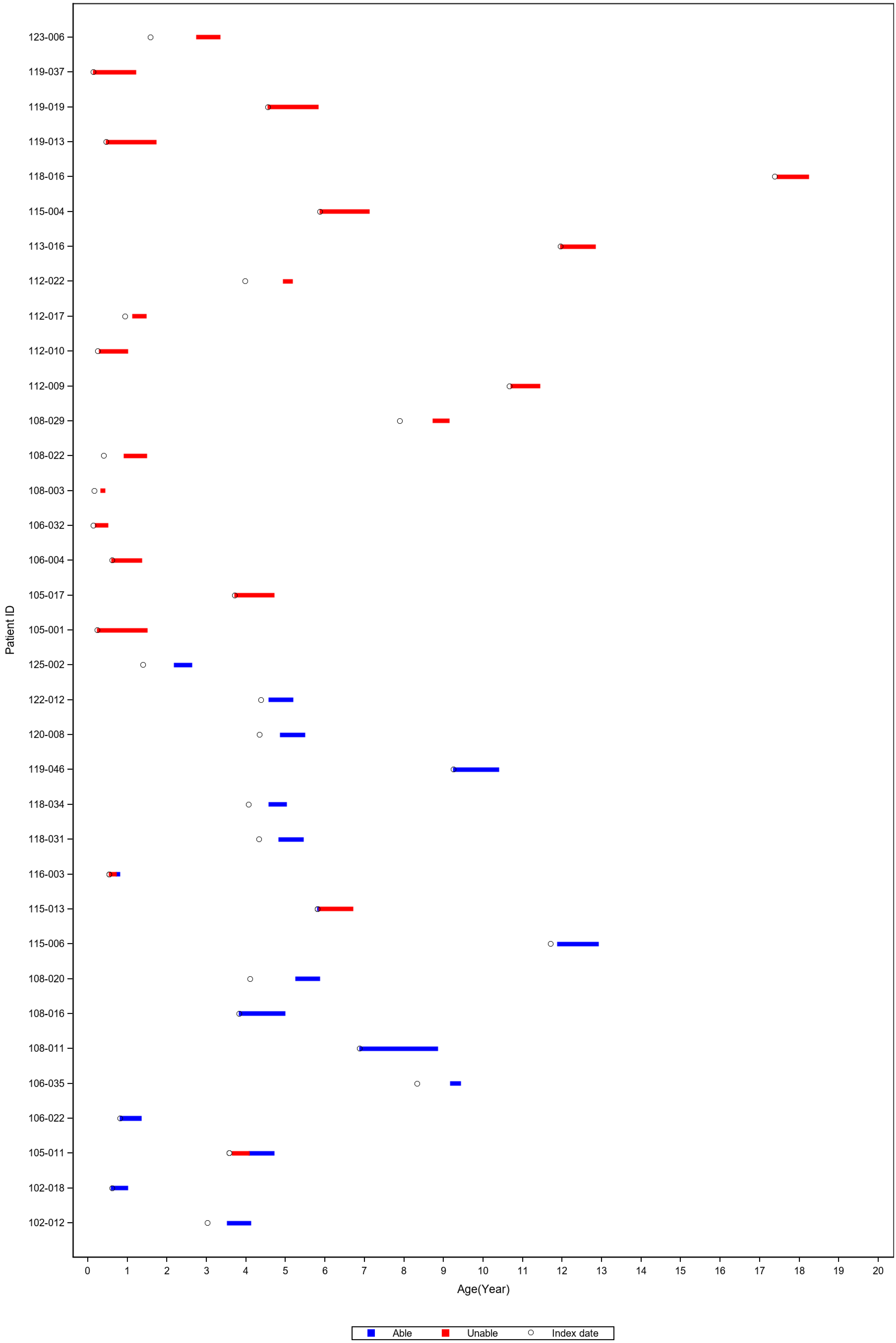

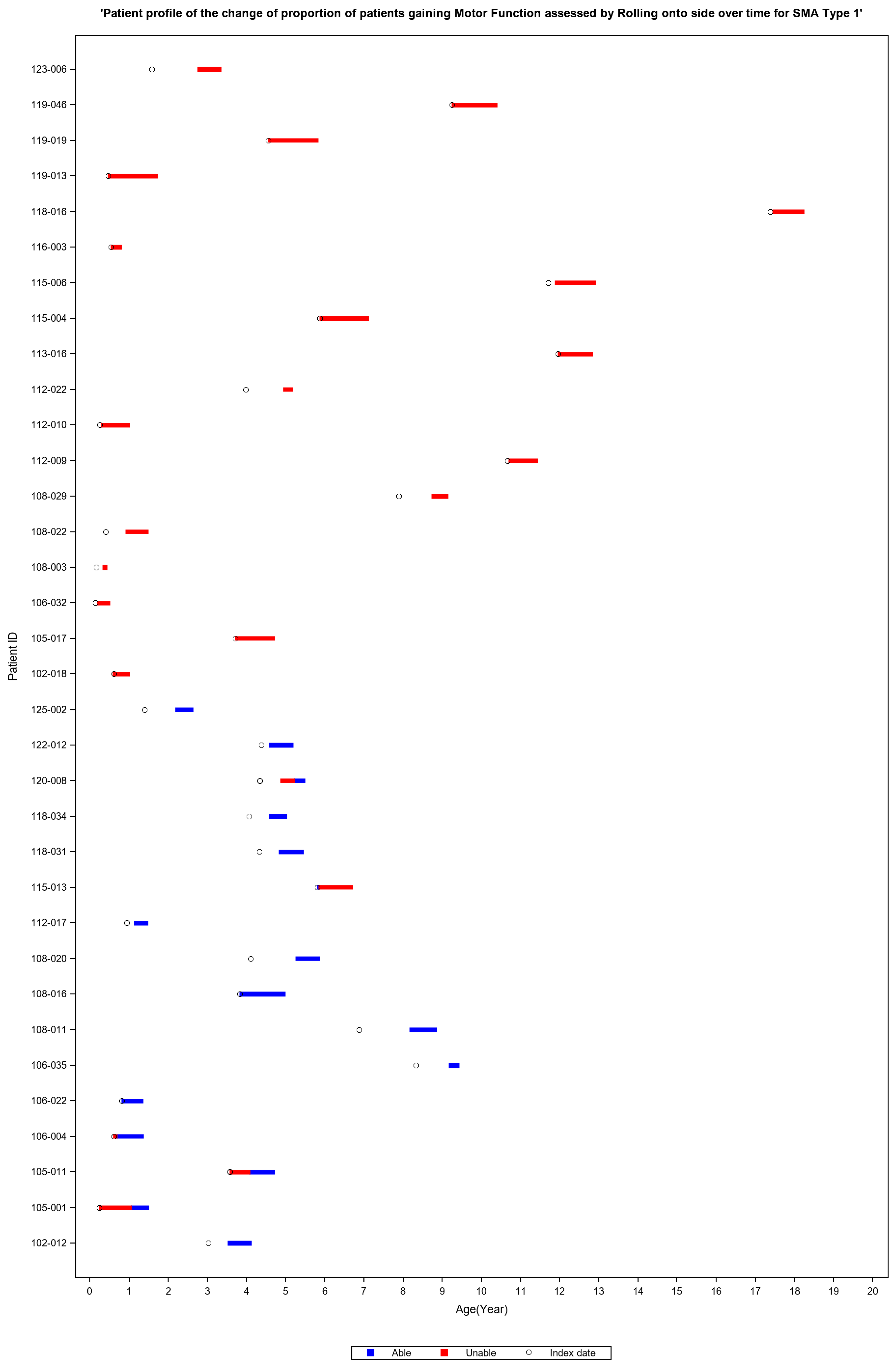
'Patient profile of the change of proportion of patients gaining Motor Function assessed by Rolling onto side over time for SMA Type 1'
Patient ID
123-006
119-046
119-019
119-013
118-016
116-003
115-006
115-004
113-016
112-022
112-010
112-009
108-029
108-022
108-003
106-032
105-017
102-018
125-002
122-012
120-008
118-034
118-031
115-013
112-017
108-020
108-016
108-011
106-035
106-022
106-004
105-011
105-001
102-012
0 1 2 3 4 5 6 7 8 9 10 11 12 13 14 15 16 17 18 19 20
Age(Year)
Able
Unable
Index date

'Patient profile of the change of proportion of patients gaining Motor Function assessed by Able to walk 10 meters unaided over time for SMA Type 1'

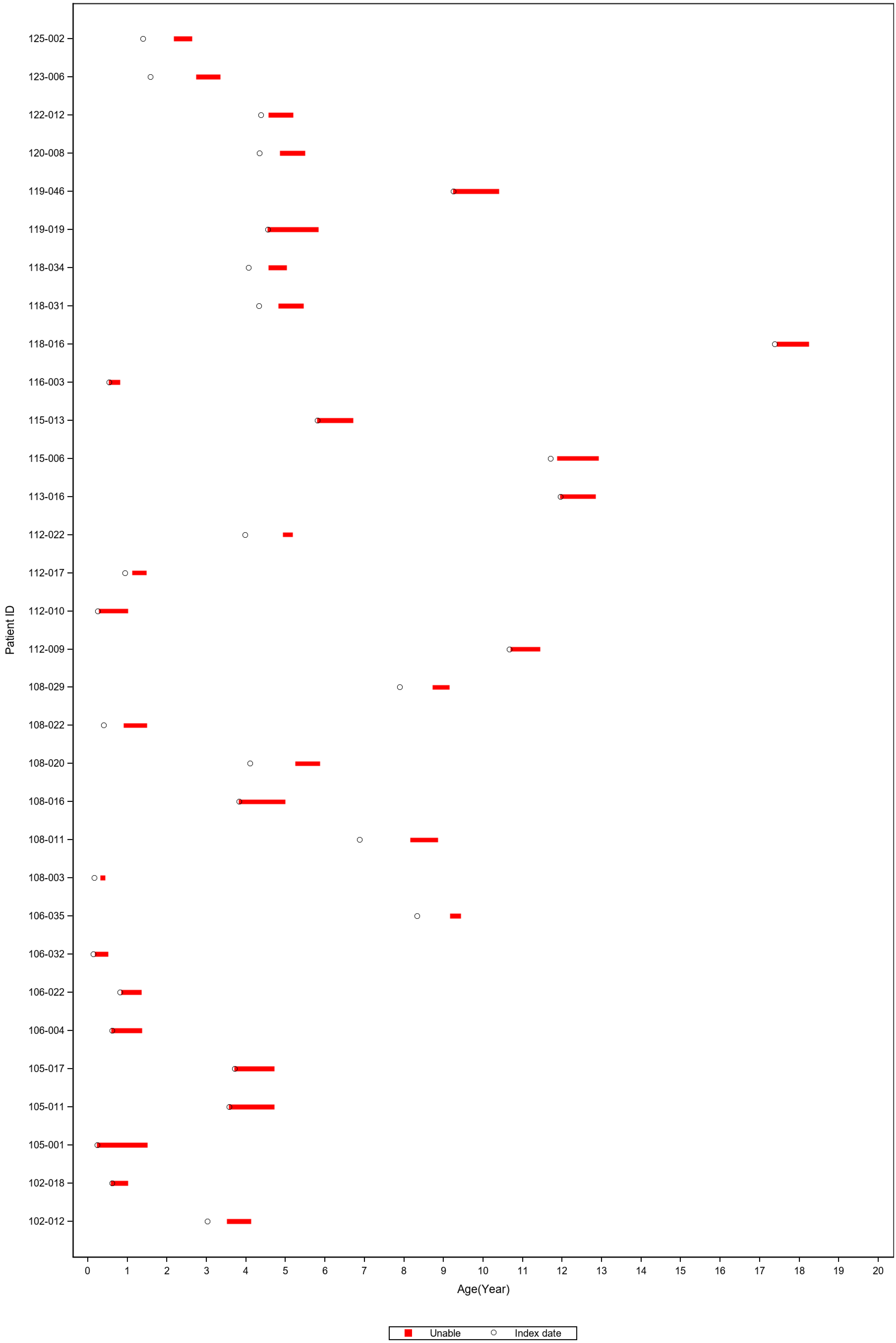

'Patient profile of the change of proportion of patients gaining Motor Function assessed by Climbing stairs over time for SMA Type 1'

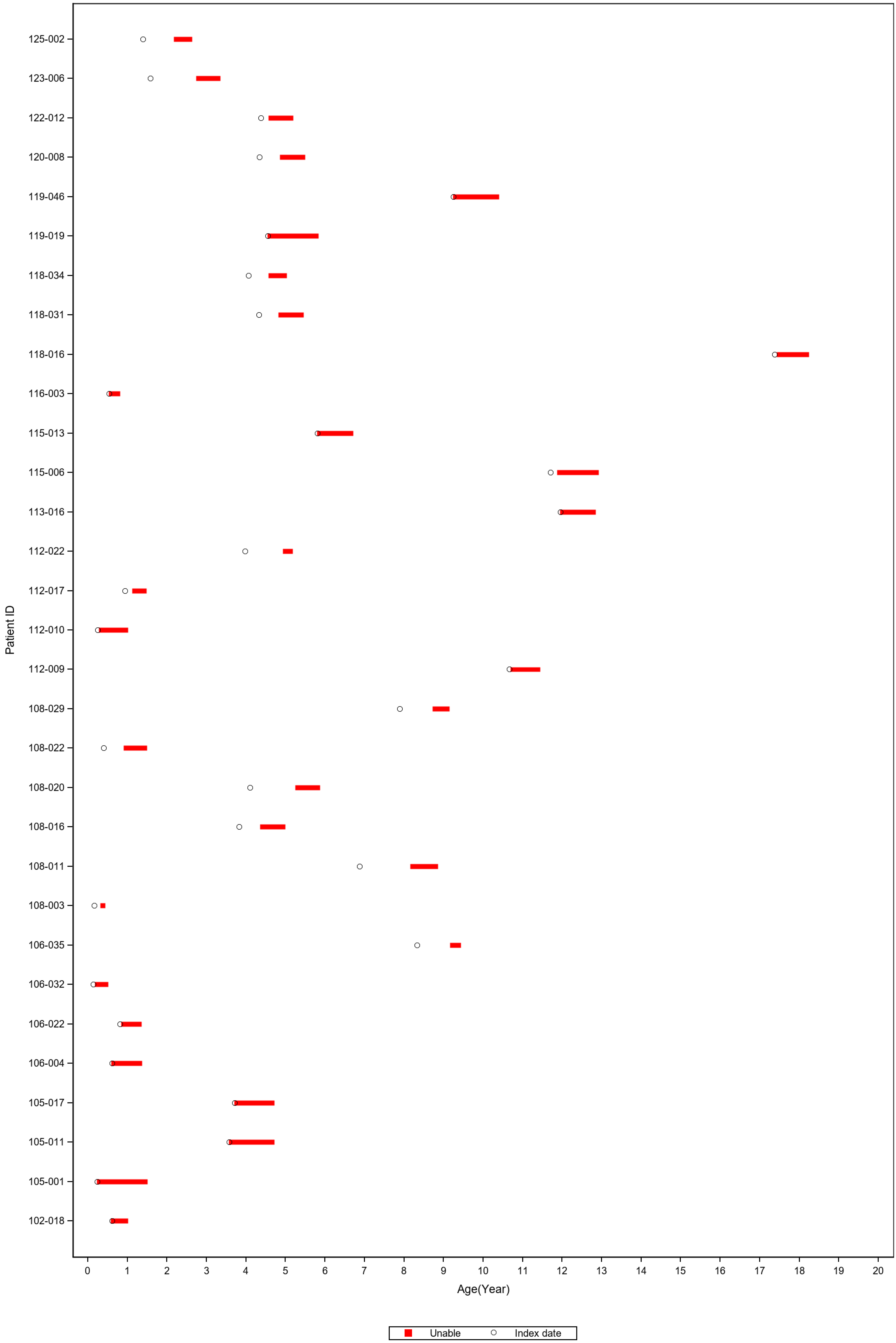

'Patient profile of the change of proportion of patients gaining Motor Function assessed by Useful function of hands over time for SMA Type 1'

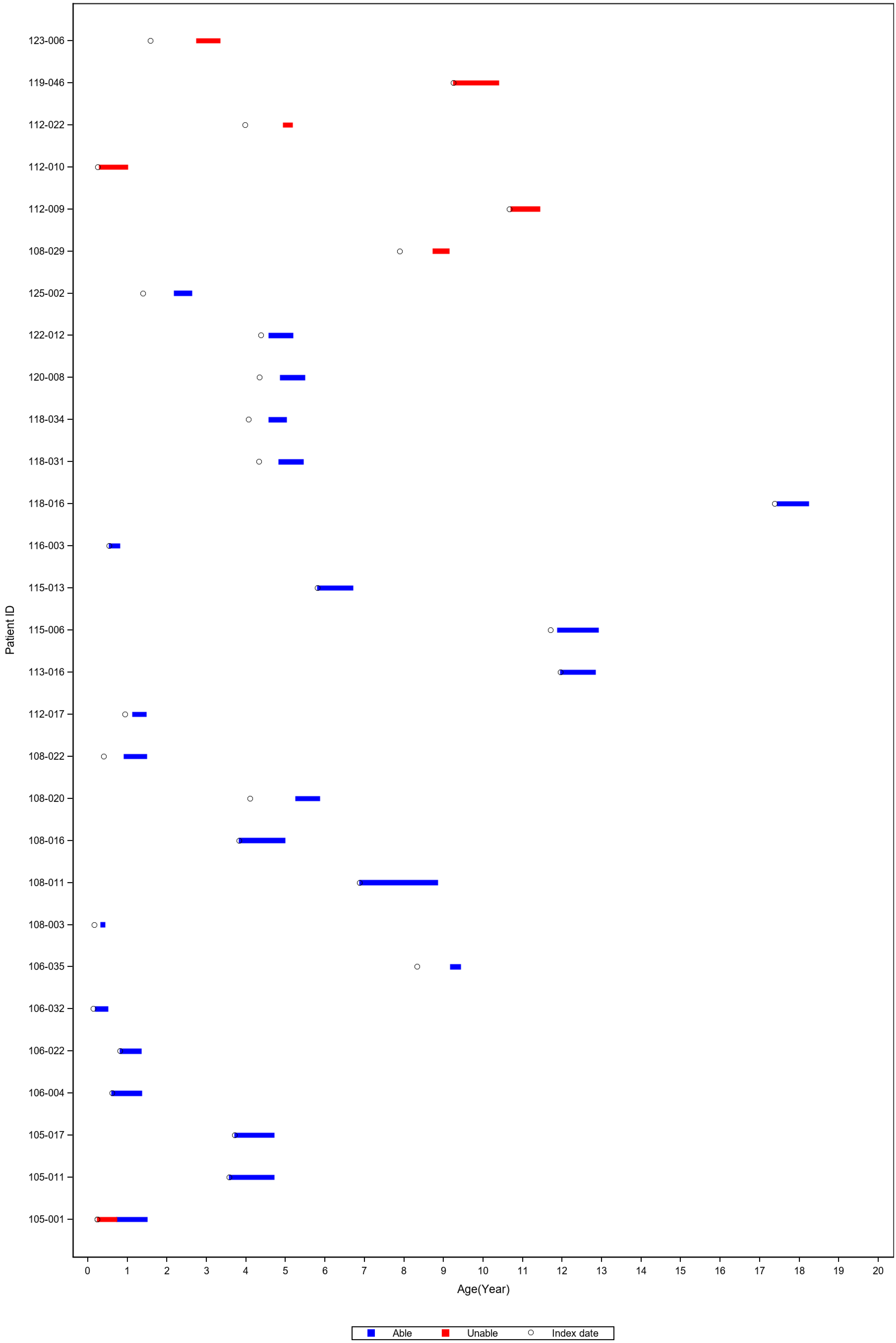

'Patient profile of the change of proportion of patients gaining Motor Function assessed by Reaching overhead in a sitting position over time for SMA Type 1'

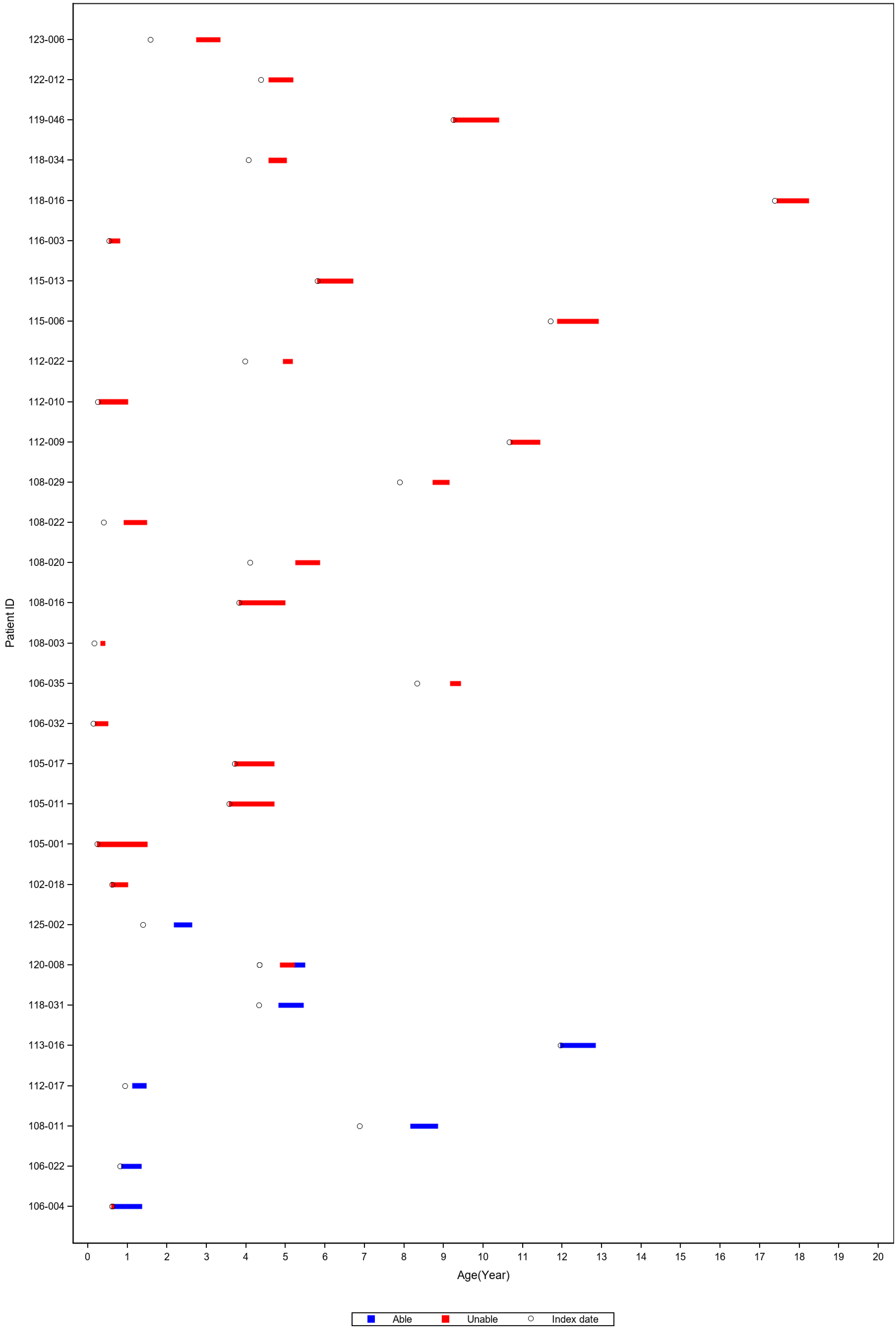

'Patient profile of the change of proportion of patients gaining Motor Function assessed by Raising hands to mouth in a sitting position over time for SMA Type 1'

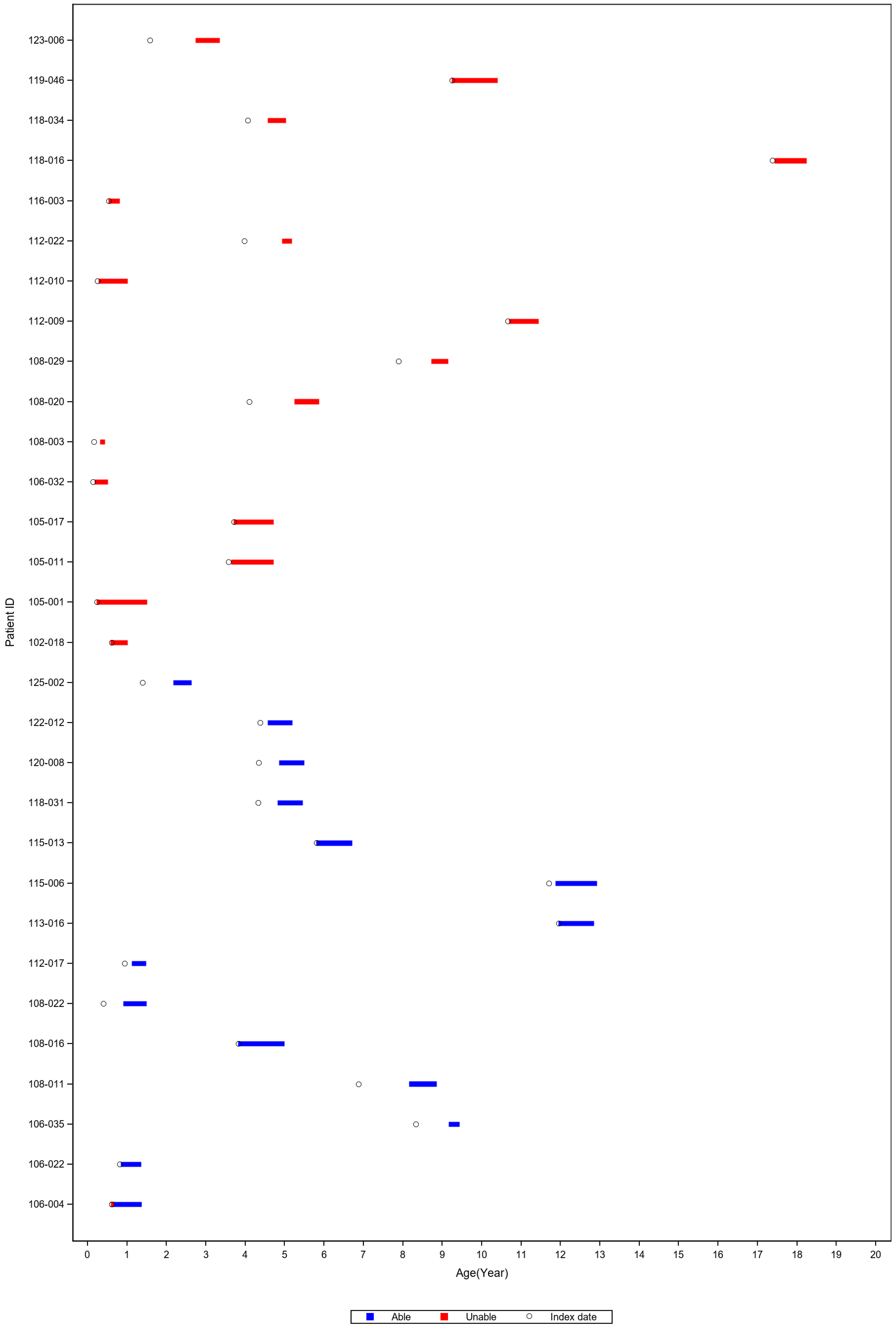

'Patient profile of the change of proportion of patients gaining Motor Function assessed by Holding Head Up without Support over time for SMA Type 2'

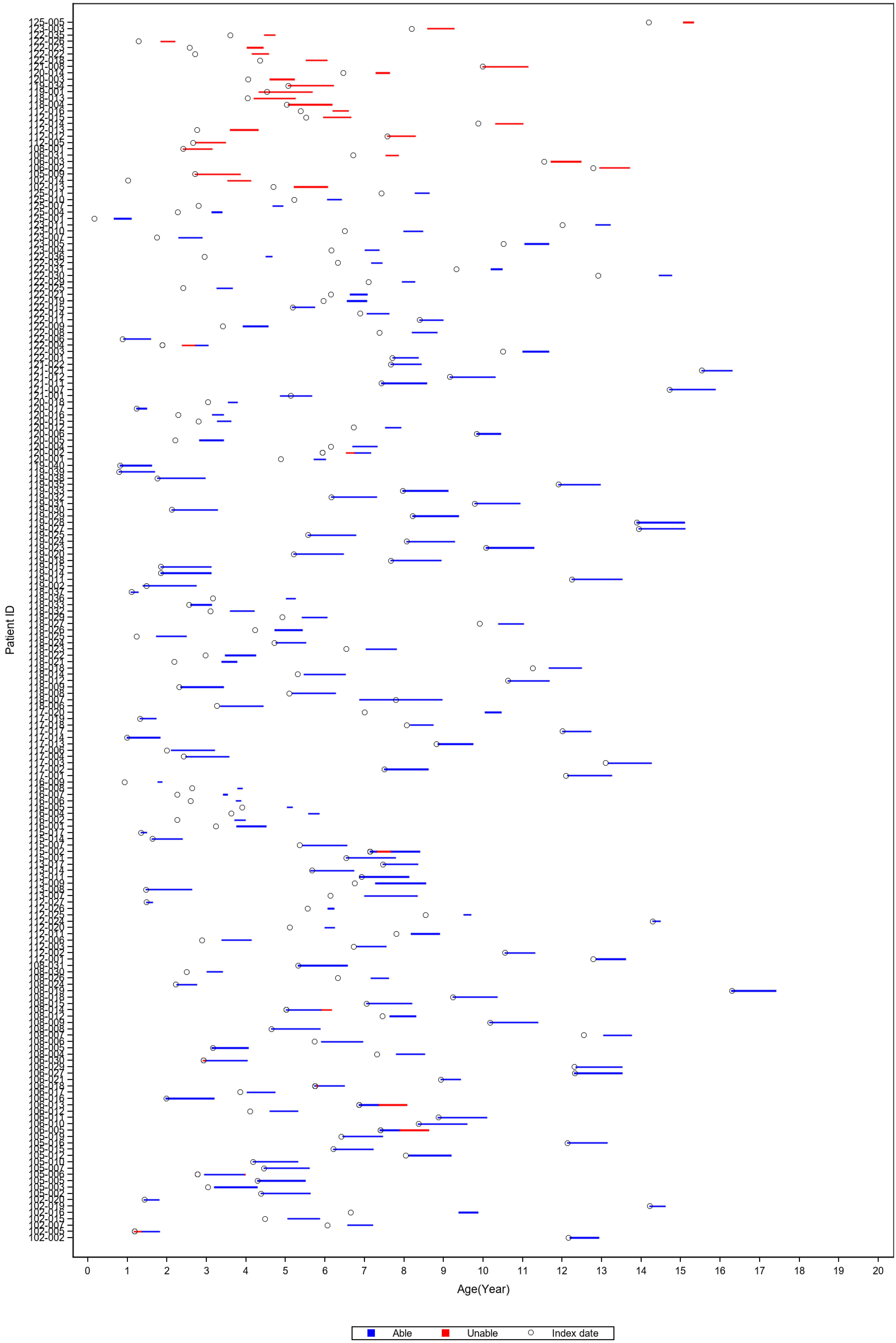

'Patient profile of the change of proportion of patients gaining Motor Function assessed by Rolling onto side over time for SMA Type 2'

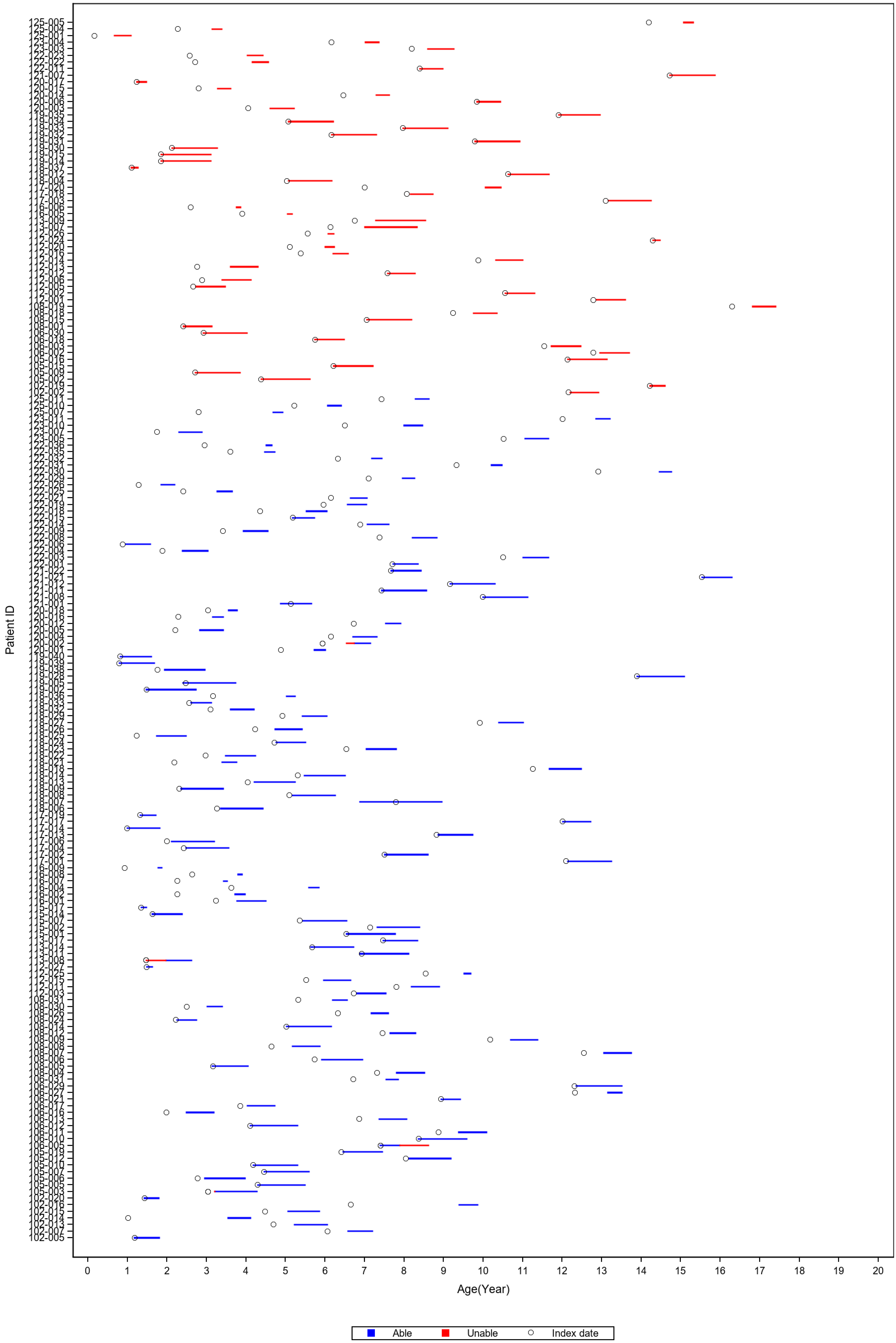

'Patient profile of the change of proportion of patients gaining Motor Function assessed by Able to walk 10 meters unaided over time for SMA Type 2'

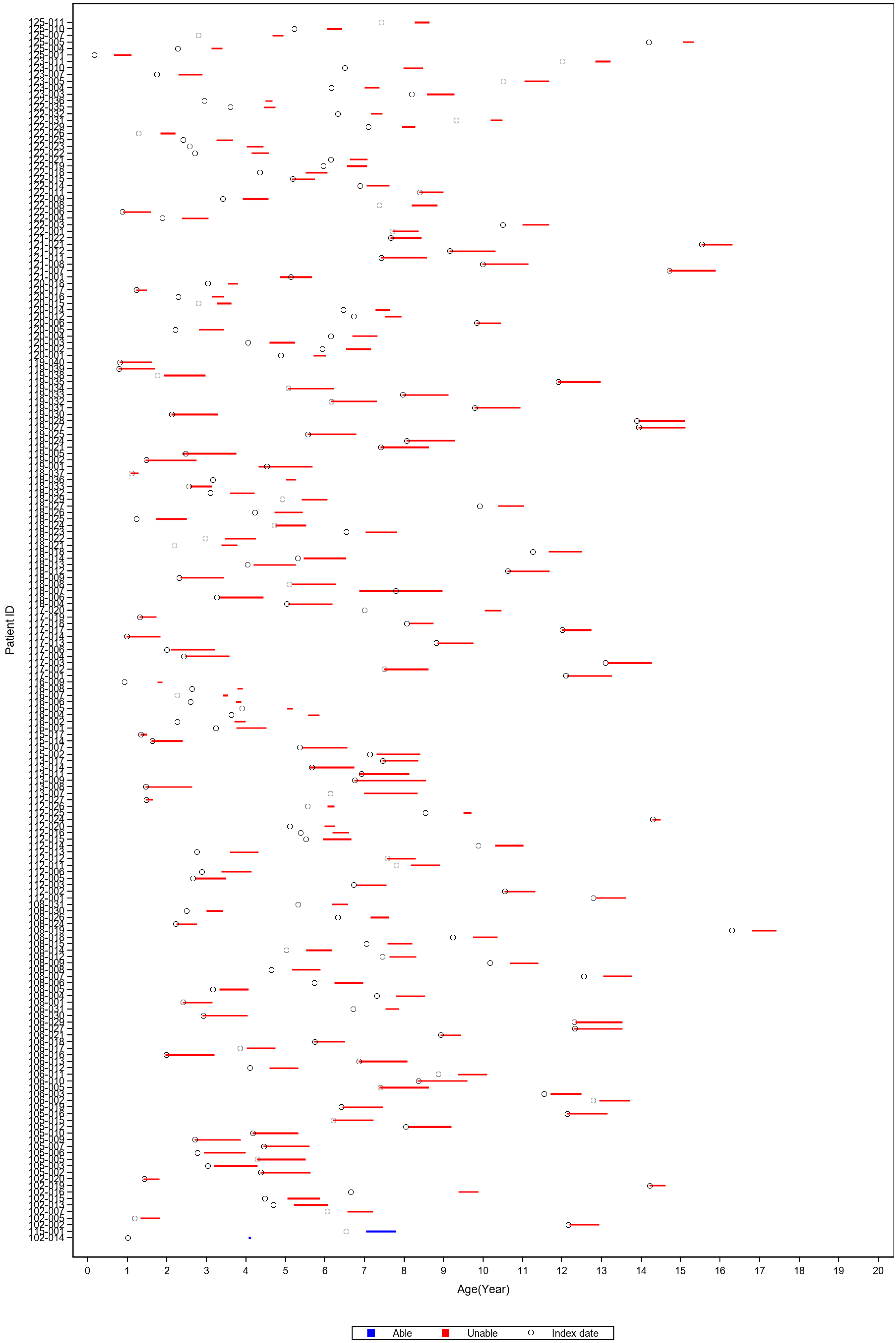

'Patient profile of the change of proportion of patients gaining Motor Function assessed by Climbing stairs over time for SMA Type 2'

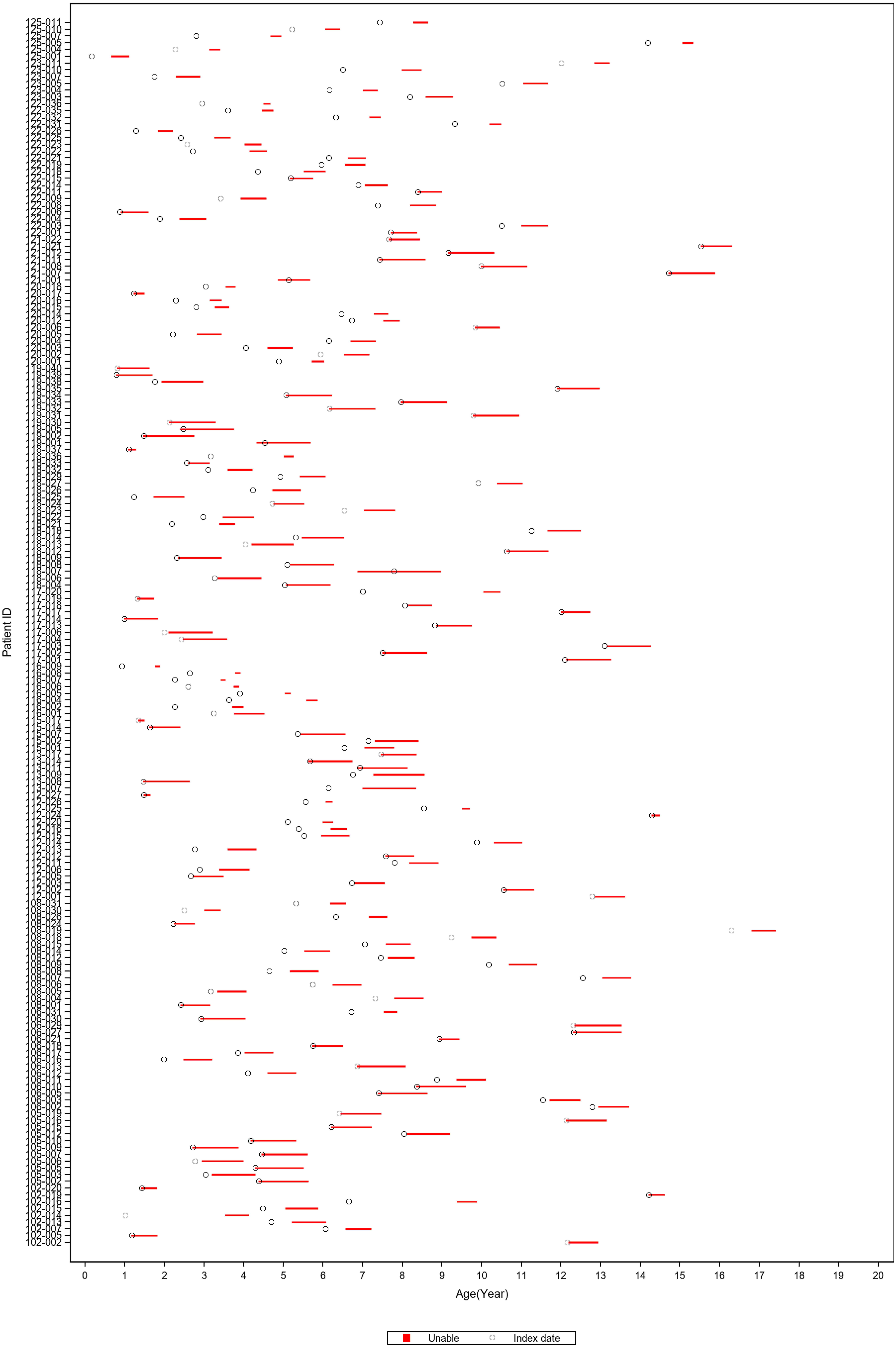

'Patient profile of the change of proportion of patients gaining Motor Function assessed by Useful function of hands over time for SMA Type 2'

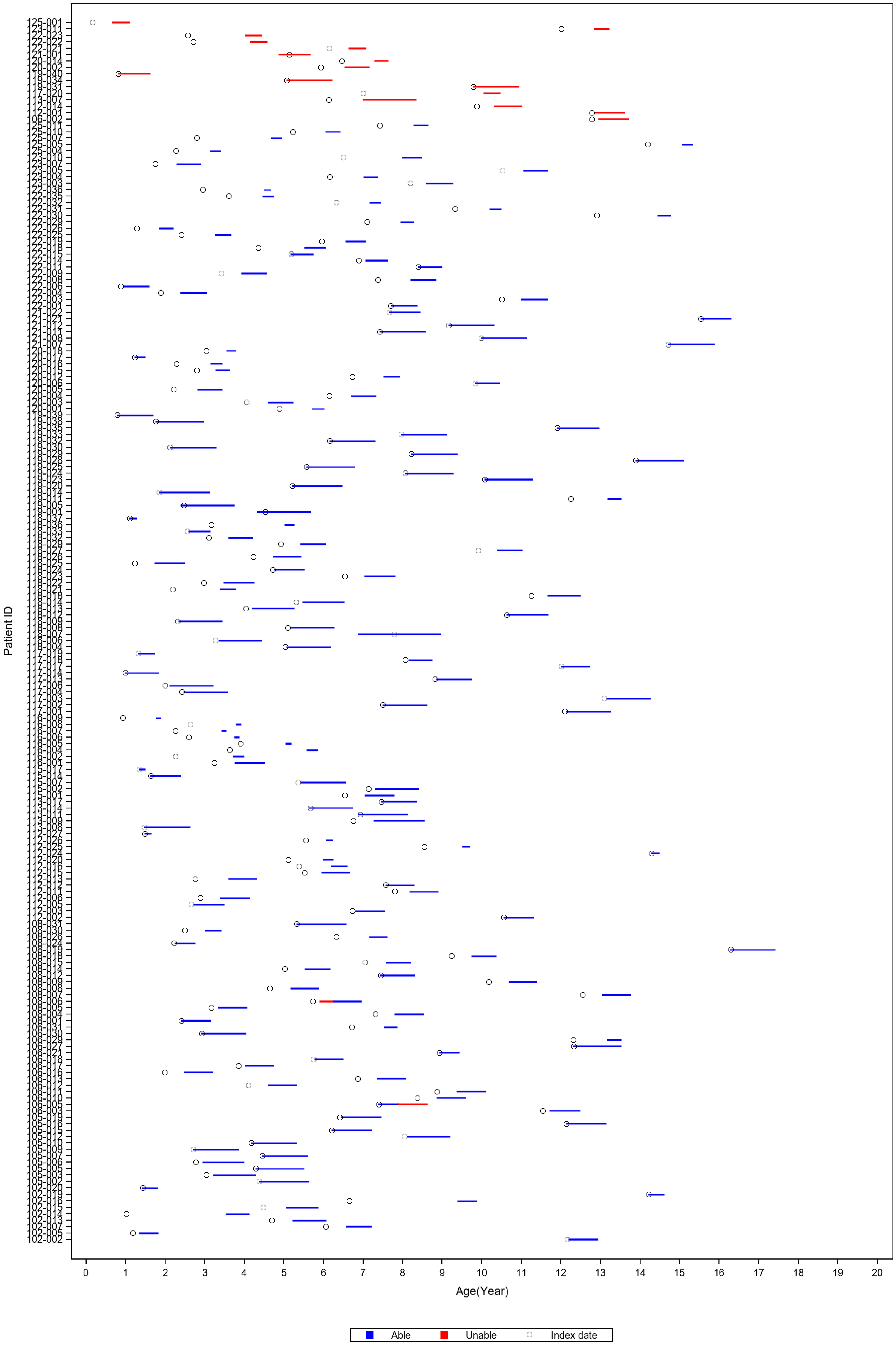

'Patient profile of the change of proportion of patients gaining Motor Function assessed by Reaching overhead in a sitting position over time for SMA Type 2'

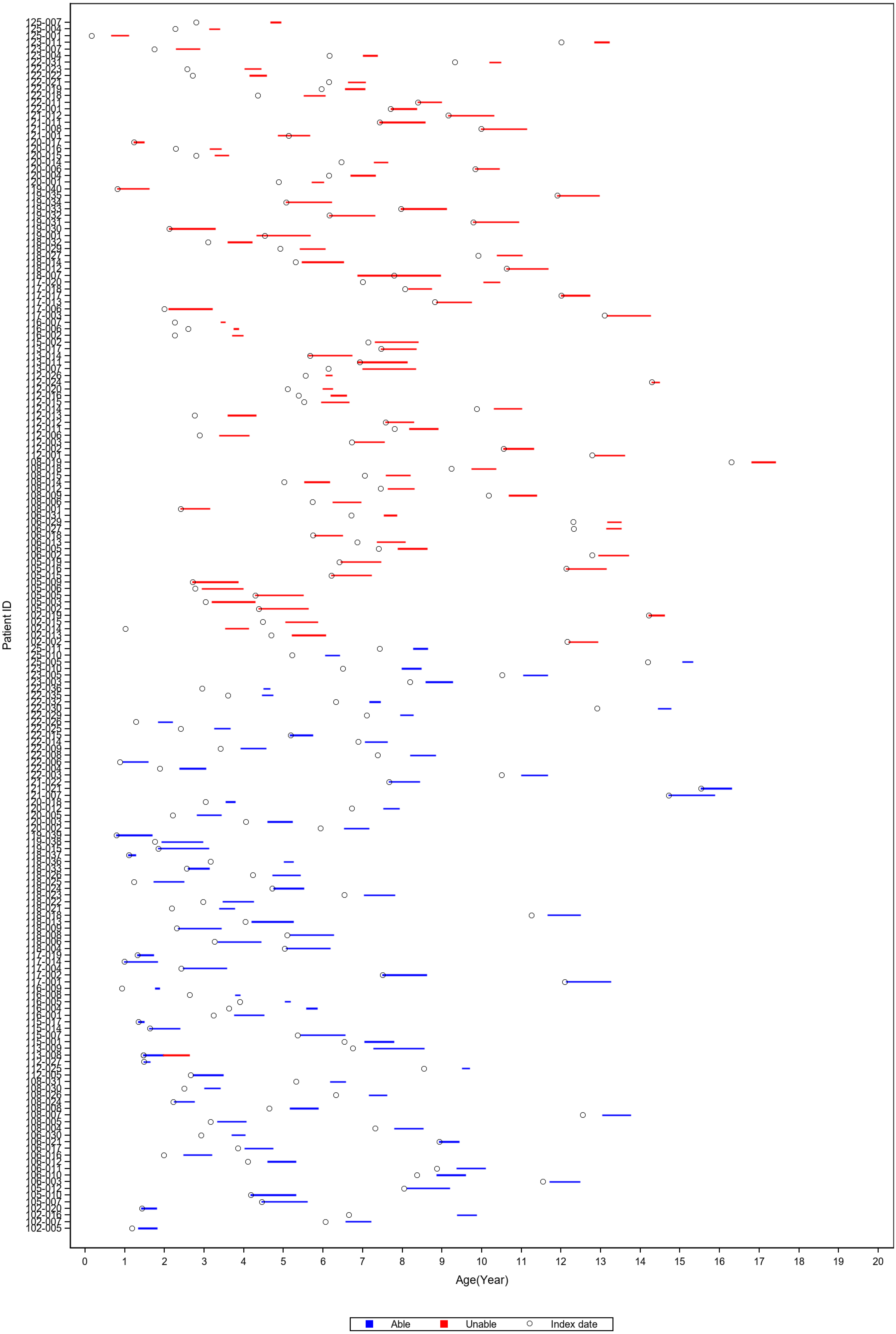

'Patient profile of the change of proportion of patients gaining Motor Function assessed by Raising hands to mouth in a sitting position over time for SMA Type 2'

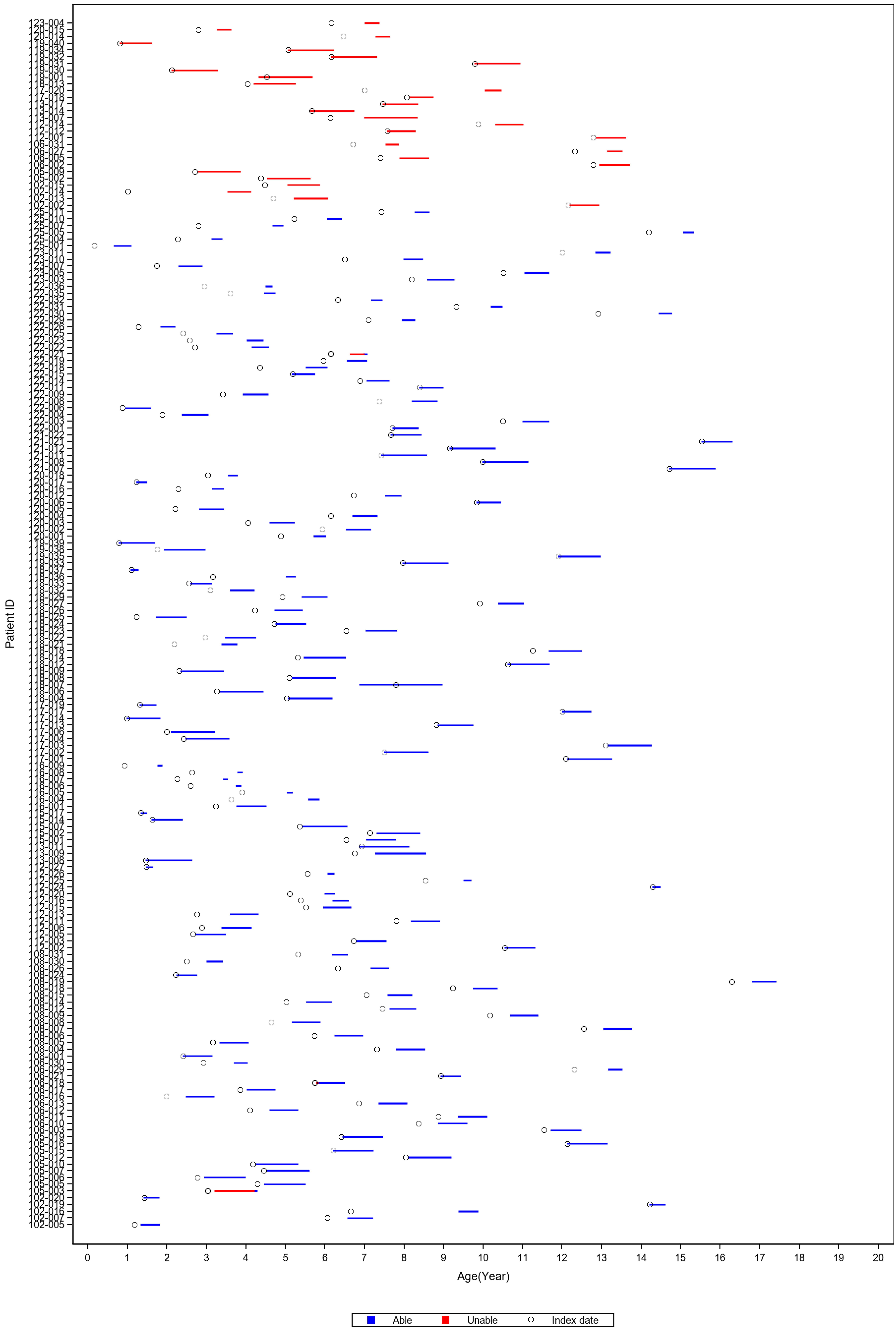

'Patient profile of the change of proportion of patients gaining Motor Function assessed by Holding Head Up without Support over time for SMA Type 3'

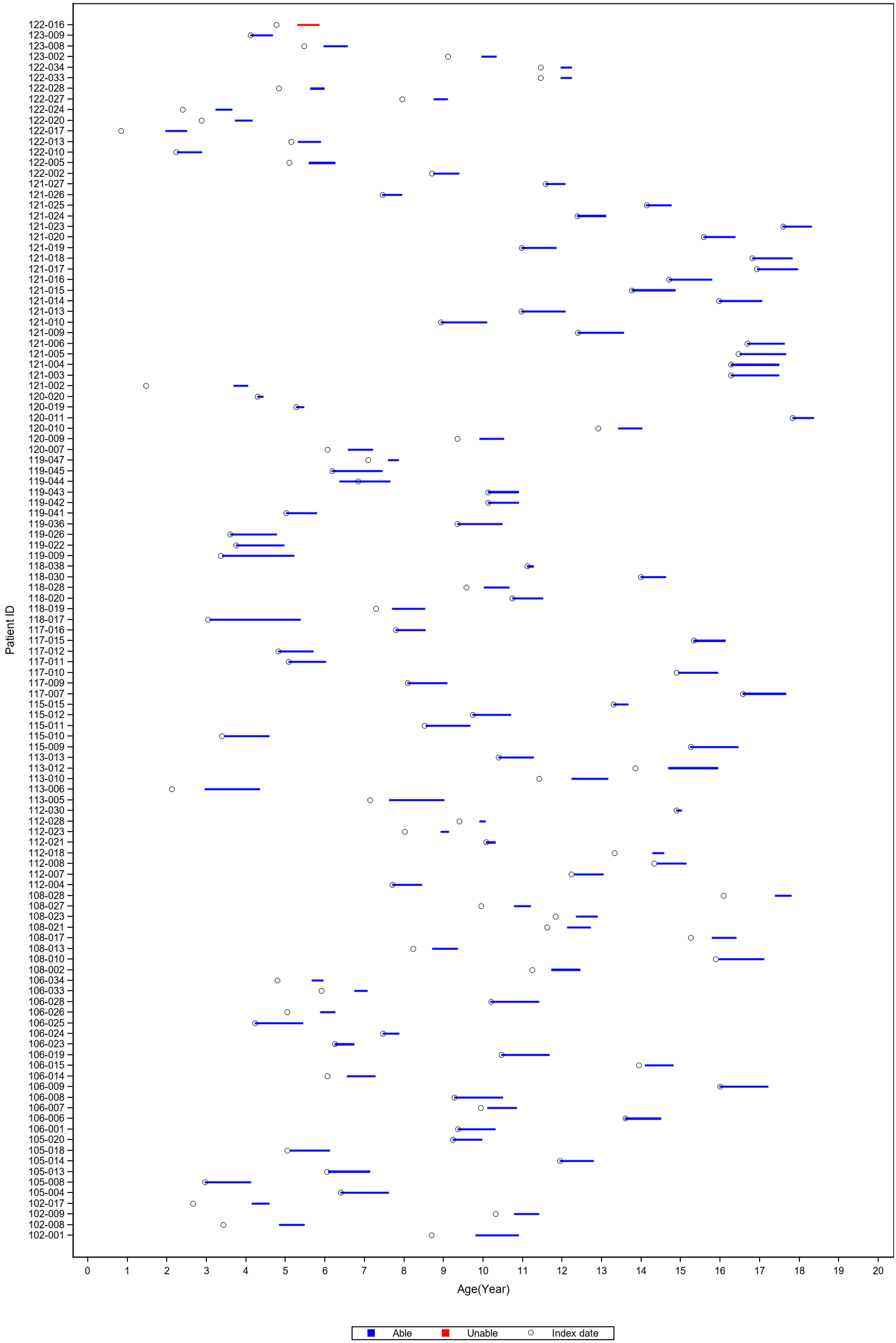

'Patient profile of the change of proportion of patients gaining Motor Function assessed by Rolling onto side over time for SMA Type 3'

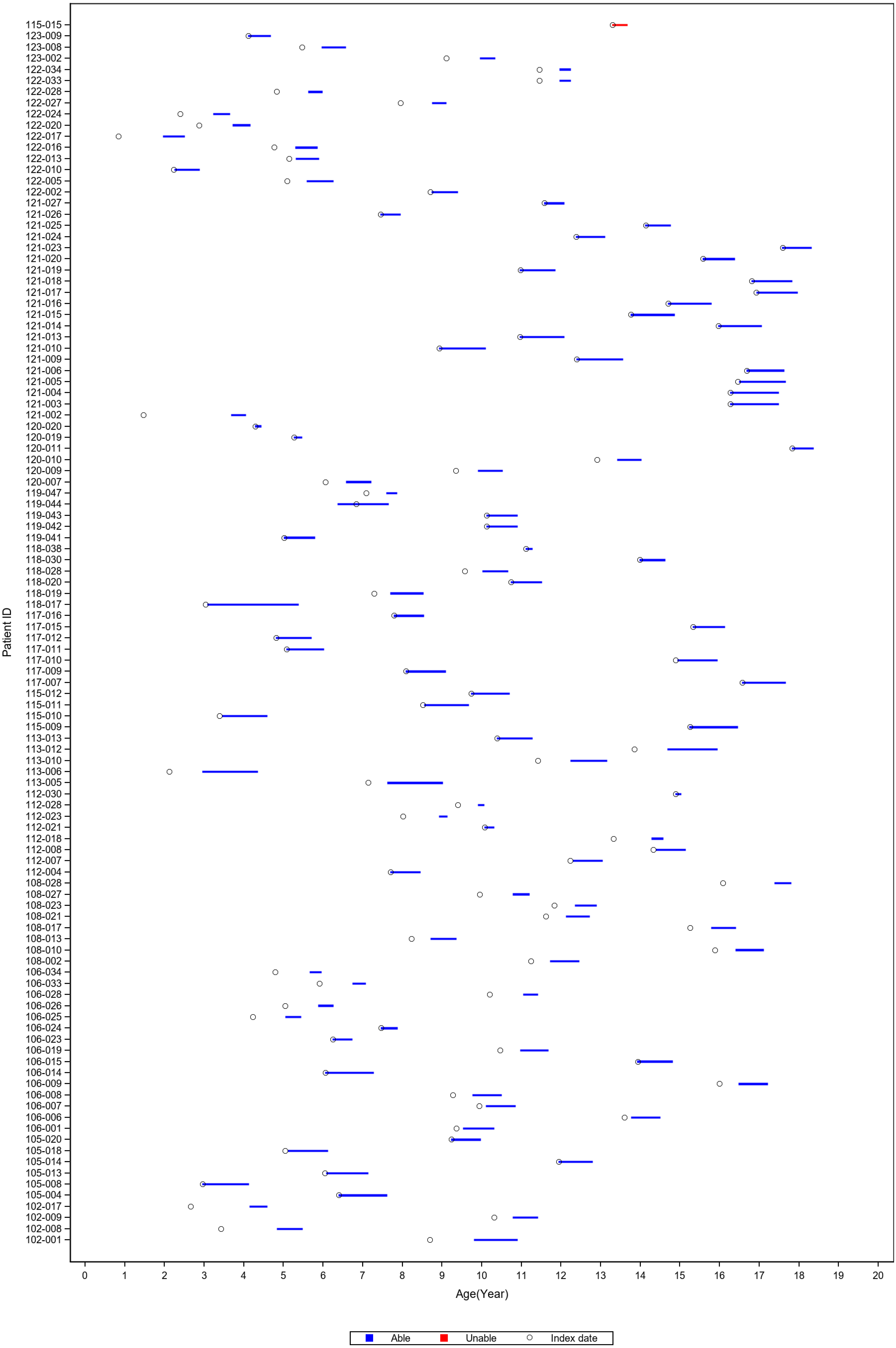

'Patient profile of the change of proportion of patients gaining Motor Function assessed by Able to walk 10 meters unaided over time for SMA Type 3'

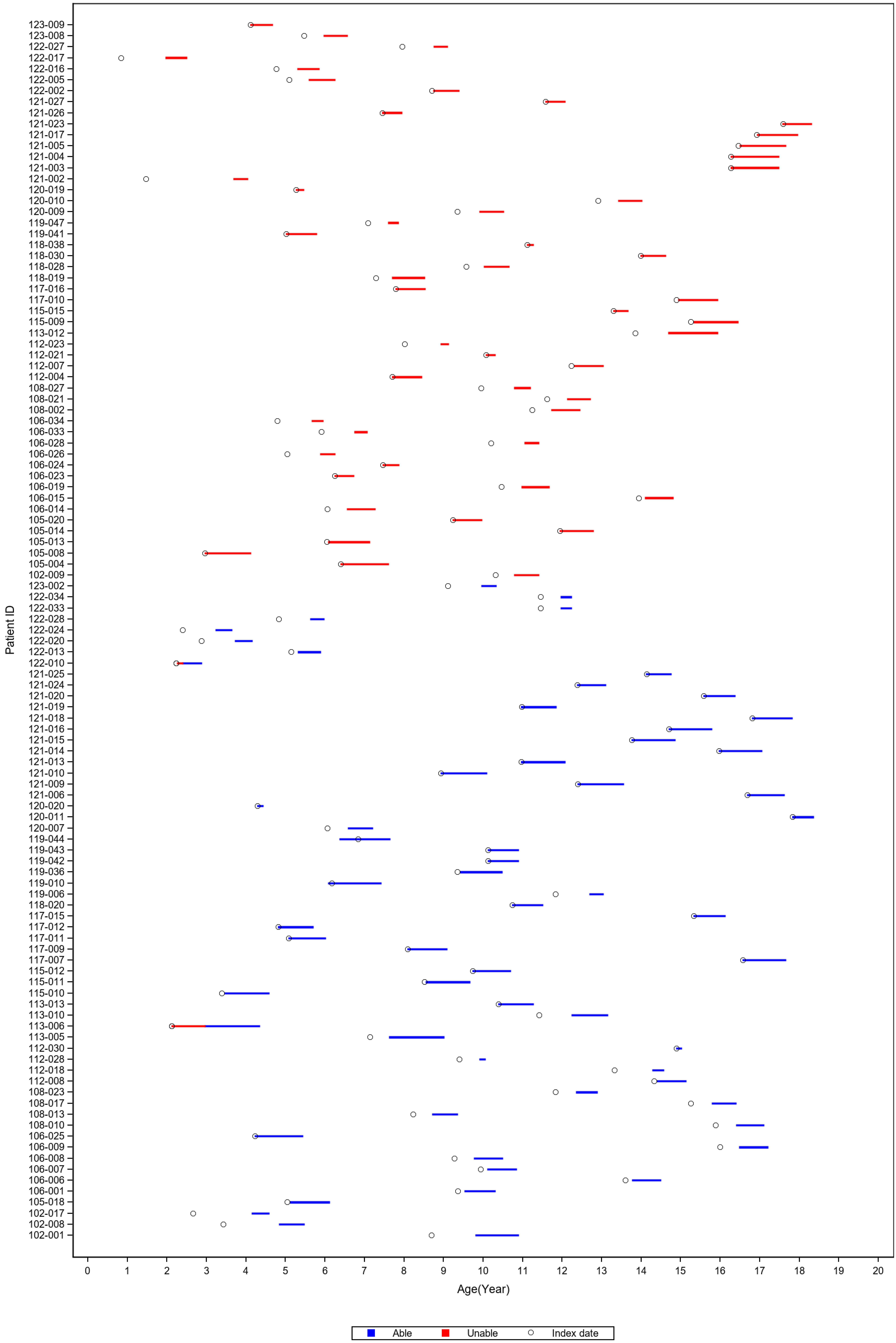

'Patient profile of the change of proportion of patients gaining Motor Function assessed by Climbing stairs over time for SMA Type 3'

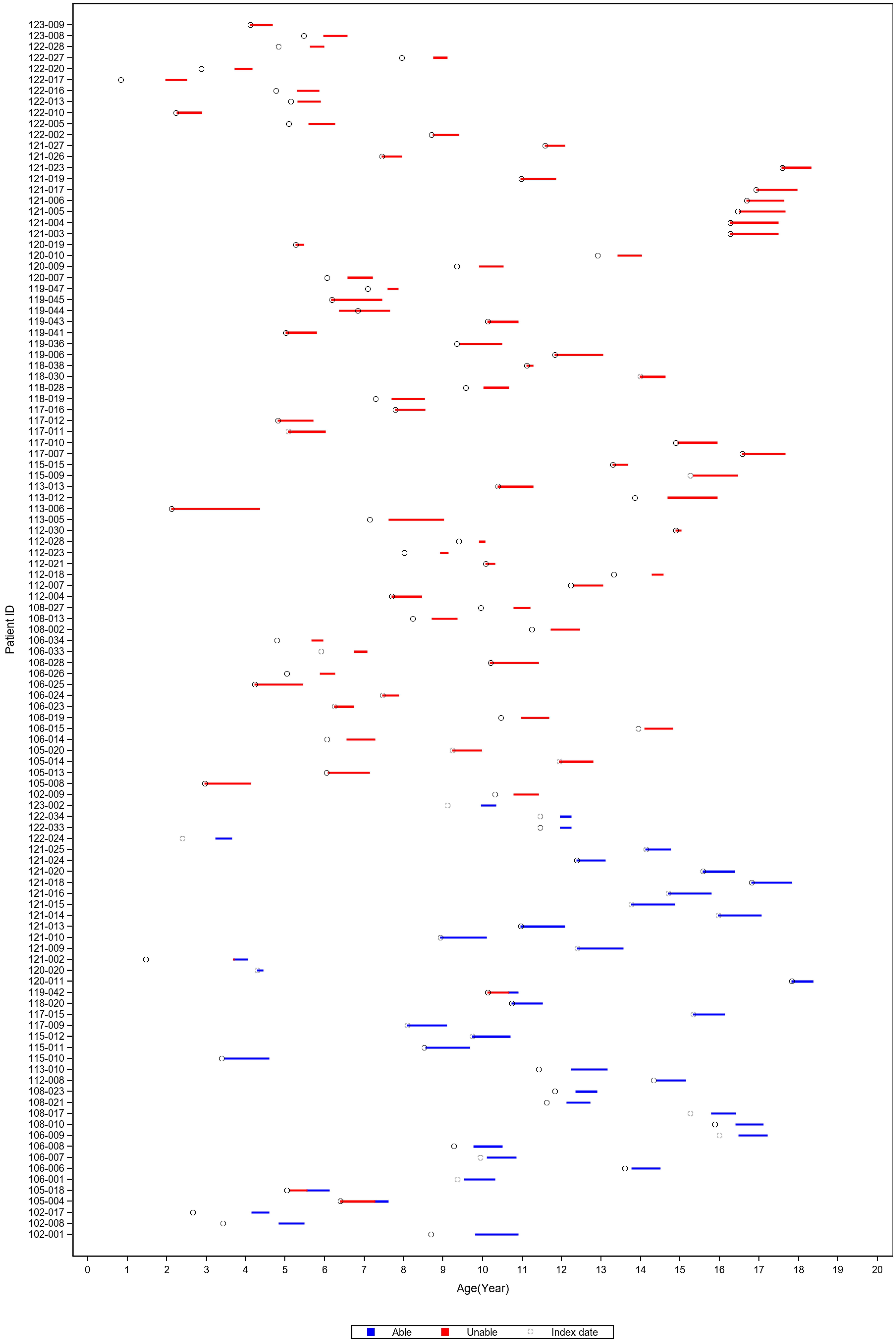

'Patient profile of the change of proportion of patients gaining Motor Function assessed by Useful function of hands over time for SMA Type 3'

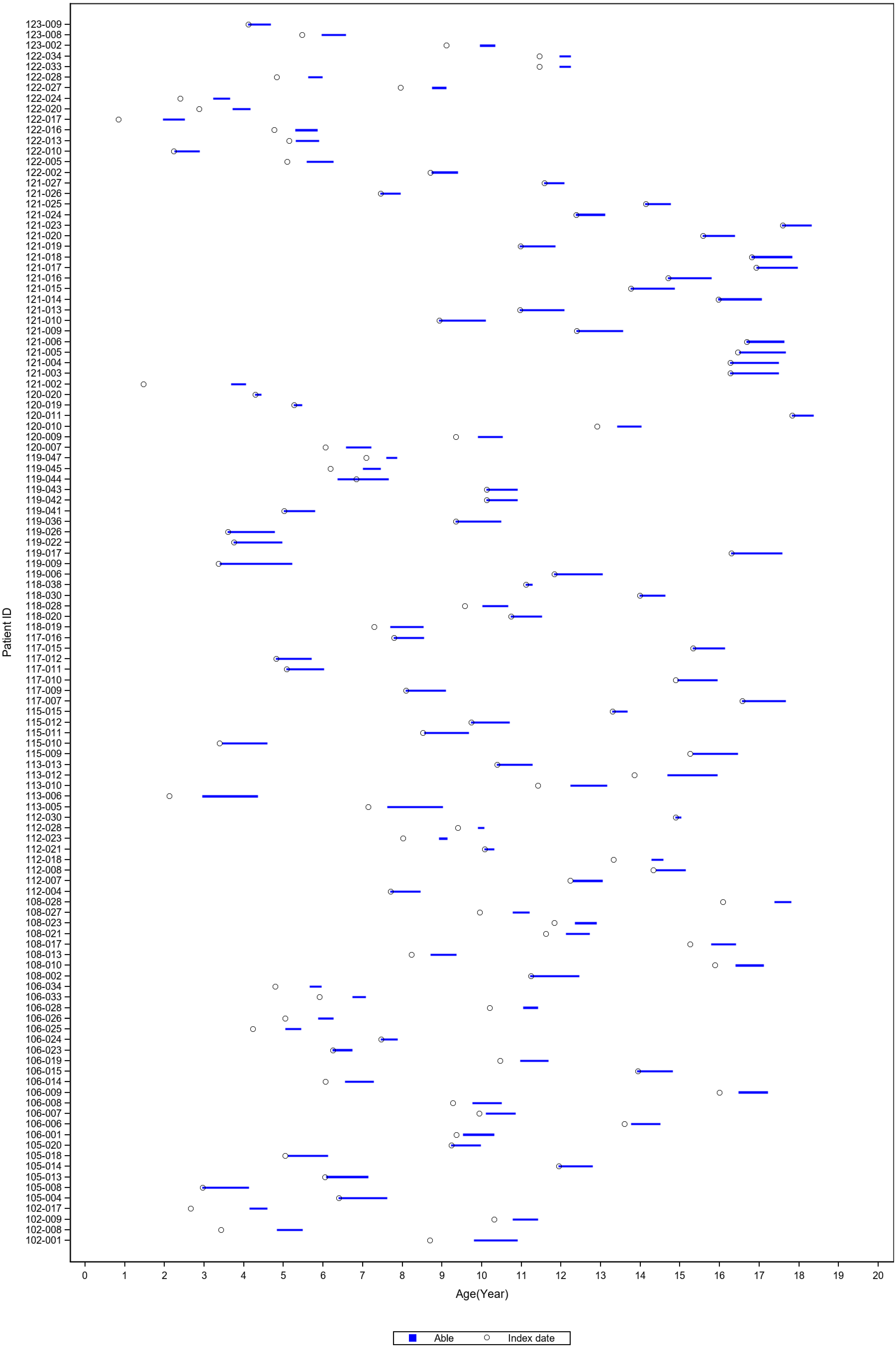

'Patient profile of the change of proportion of patients gaining Motor Function assessed by Reaching overhead in a sitting position over time for SMA Type 3'

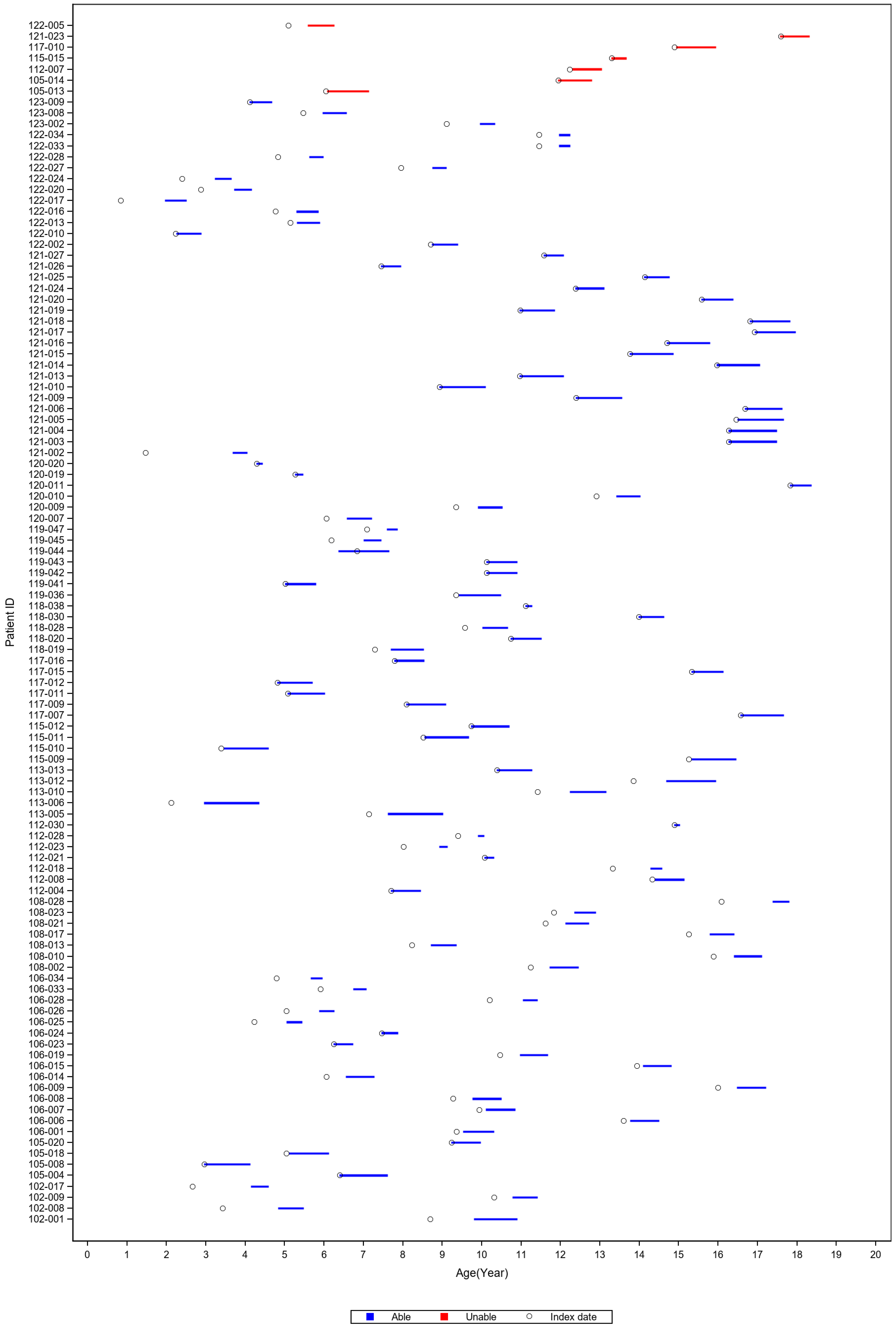

'Patient profile of the change of proportion of patients gaining Motor Function assessed by Raising hands to mouth in a sitting position over time for SMA Type 3'

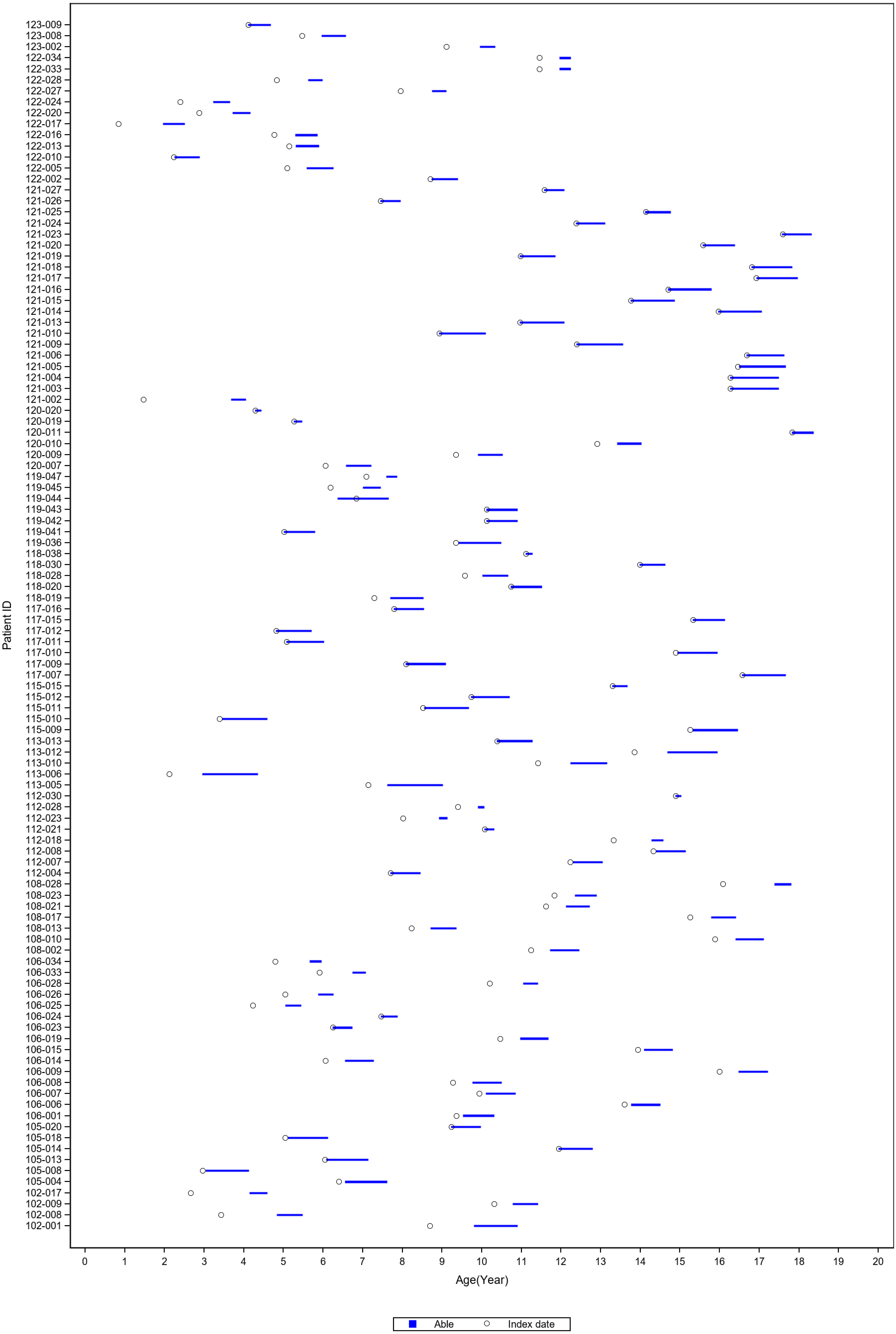

Supplement: Supplementary file 2 — Supplementary file2 (PDF 5091 KB) [file 415_2024_12442_MOESM2_ESM.pdf]
