# Supplementary material for: Nusinersen effectiveness and safety in pediatric patients with 5q-spinal muscular atrophy: a multi-center disease registry in China
Source: J Neurol. 2024 Jul 2;271(8):5378–91. doi: 10.1007/s00415-024-12442-w (PMC11319379; doi:10.1007/s00415-024-12442-w)
Supplement: Supplementary file 3 — Supplementary file3 (PDF 306 KB) [file 415_2024_12442_MOESM3_ESM.pdf]

Table 1a. Score change of motor measure from baseline to each visit for patients with SMA Type I and age less than 7 months

	M6	M10	M14
**Score change in CHOP-INTEND from baseline**			
Nx	1	2	1
Mean (SD)	23.0 (-)	18.5 (12.02)	32.0 (-)
Median	23	18.5	32
Q1, Q3	23, 23	10, 27	32, 32
Min, Max	23, 23	10, 27	32, 32
**Score change in HINE-2 score from baseline**			
Nx	1	2	1
Mean (SD)	3.0 (-)	2.5 (0.71)	4.0 (-)
Median	3	2.5	4
Q1, Q3	3, 3	2, 3	4, 4
Min, Max	3, 3	2, 3	4, 4

Table 1b. Score change of motor measure from baseline to each visit for patients with SMA Type I and age more than 7 months

	M6	M10	M14
**Score change in CHOP-INTEND from baseline**			
Nx	5	2	2
Mean (SD)	3.4 (4.28)	8.5 (3.54)	5.5 (3.54)
Median	2	8.5	5.5
Q1, Q3	1, 2	6, 11	3, 8
Min, Max	1, 11	6, 11	3, 8
**Score change in HINE-2 score from baseline**			
Nx	4	2	0
Mean (SD)	0.0 (2.16)	0.0 (0.00)	-
Median	-0.5	0	-
Q1, Q3	-1.5, 1.5	0, 0	-
Min, Max	-2, 3	0, 0	-

Table 2a. Score change of motor measure from baseline to each visit for patients with SMA Type I and two SMN2 copy numbers

	M6	M10	M14
**Score change in CHOP-INTEND from baseline**			
Nx	3	2	1
Mean (SD)	8.7 (12.42)	16.5 (14.85)	32.0 (-)
Median	2	16.5	32
Q1, Q3	1, 23	6, 27	32, 32
Min, Max	1, 23	6, 27	32, 32
**Score change in HINE-2 score from baseline**			
Nx	3	2	1
Mean (SD)	0.7 (2.08)	1.5 (2.12)	4.0 (-)
Median	0	1.5	4
Q1, Q3	-1, 3	0, 3	4, 4
Min, Max	-1, 3	0, 3	4, 4

Table 2b. Score change of motor measure from baseline to each visit for patients with SMA Type I and three SMN2 copy numbers

	M6	M10	M14
**Score change in CHOP-INTEND from baseline**			
Nx	2	2	1
Mean (SD)	6.5 (6.36)	10.5 (0.71)	8.0 (-)
Median	6.5	10.5	8
Q1, Q3	2, 11	10, 11	8, 8
Min, Max	2, 11	10, 11	8, 8
**Score change in HINE-2 score from baseline**			
Nx	2	2	0
Mean (SD)	0.5 (3.54)	1.0 (1.41)	-
Median	0.5	1	-
Q1, Q3	-2, 3	0, 2	-
Min, Max	-2, 3	0, 2	-

Table 3a. Score change of motor measure from baseline to each visit for patients with SMA Type II and age less than 5 years old

	M6	M10	M14
**Score change in HFMSE score from baseline**			
Nx	25	17	9
Mean (SD)	5.5 (5.53)	6.8 (5.07)	8.2 (5.36)
Median	6	5	7
Q1, Q3	3, 9	3, 10	5, 11
Min, Max	-15, 15	1, 16	1, 18
**Score change in RULM from baseline**			
Nx	16	9	6
Mean (SD)	3.3 (2.65)	4.3 (3.39)	5.7 (8.09)
Median	3	4	2.5
Q1, Q3	1.5, 4.5	2, 6	1, 8
Min, Max	0, 10	0, 11	-1, 21

Table 3b. Score change of motor measure from baseline to each visit for patients with SMA Type II and age between 5 to 13 years old

	M6	M10	M14
**Score change in HFMSE score from baseline**			
Nx	45	26	7
Mean (SD)	3.6 (3.45)	2.1 (2.79)	1.0 (2.83)
Median	3	1.5	2
Q1, Q3	1, 5	1, 4	-2, 3
Min, Max	-2, 12	-2, 10	-3, 5
**Score change in RULM from baseline**			
Nx	42	22	5
Mean (SD)	2.2 (2.85)	1.8 (2.44)	2.4 (2.70)
Median	2.5	2	3
Q1, Q3	0, 4	-1, 3	2, 4
Min, Max	-5, 9	-2, 8	-2, 5

Table 3c. Score change of motor measure from baseline to each visit for patients with SMA Type II and age more than 13 years old

	M6	M10
**Score change in HFMSE score from baseline**		
Nx	2	1
Mean (SD)	9.5 (2.12)	10.0 (-)
Median	9.5	10
Q1, Q3	8, 11	10, 10
Min, Max	8, 11	10, 10
**Score change in RULM from baseline**		
Nx	3	1
Mean (SD)	-0.7 (5.13)	-4.0 (-)
Median	-2	-4
Q1, Q3	-5, 5	-4, -4
Min, Max	-5, 5	-4, -4

Table 4a. Score change of motor measure from baseline to each visit for patients with SMA Type II and two SMN2 copy numbers

	M6	M10
**Score change in HFMSE score from baseline**		
Nx	3	3
Mean (SD)	4.0 (6.08)	3.0 (2.00)
Median	1	3
Q1, Q3	0, 11	1, 5
Min, Max	0, 11	1, 5
**Score change in RULM from baseline**		
Nx	3	2
Mean (SD)	1.0 (2.65)	2.0 (0.00)
Median	2	2
Q1, Q3	-2, 3	2, 2
Min, Max	-2, 3	2, 2

Table 4b. Score change of motor measure from baseline to each visit for patients with SMA Type II and three SMN2 copy numbers

	M6	M10	M14
**Score change in HFMSE score from baseline**			
Nx	50	27	10
Mean (SD)	4.6 (4.61)	3.6 (4.13)	3.8 (5.51)
Median	5	3	2.5
Q1, Q3	2, 8	1, 5	0, 7
Min, Max	-15, 15	-2, 16	-3, 14
**Score change in RULM from baseline**			
Nx	38	17	6
Mean (SD)	2.9 (3.26)	2.5 (3.89)	5.0 (8.17)
Median	3	3	2.5
Q1, Q3	1, 5	-1, 4	1, 5
Min, Max	-5, 10	-4, 11	-2, 21

Table 4c. Score change of motor measure from baseline to each visit for patients with SMA Type II and equal to or more than four SMN2 copy numbers

	M6	M10
**Score change in HFMSE score from baseline**		
Nx	1	1
Mean (SD)	2.0 (-)	5.0 (-)
Median	2	5
Q1, Q3	2, 2	5, 5
Min, Max	2, 2	5, 5
**Score change in RULM from baseline**		
Nx	2	1
Mean (SD)	2.0 (1.41)	3.0 (-)
Median	2	3
Q1, Q3	1, 3	3, 3
Min, Max	1, 3	3, 3

Table 5a. Score change of motor measure from baseline to each visit for patients with SMA Type III and age less than 5 years old

	M6	M10	M14
**Score change in HFMSE score from baseline**			
Nx	8	6	3
Mean (SD)	7.5 (6.44)	8.5 (8.46)	5.3 (0.58)
Median	5.5	5.5	5
Q1, Q3	2, 13	1, 18	5, 6
Min, Max	1, 18	1, 20	5, 6
**Score change in RULM from baseline**			
Nx	7	4	3
Mean (SD)	4.6 (5.88)	4.8 (5.25)	1.7 (1.53)
Median	4	3.5	2
Q1, Q3	-1, 12	1, 8.5	0, 3
Min, Max	-3, 12	0, 12	0, 3

Table 5b. Score change of motor measure from baseline to each visit for patients with SMA Type III and age between 5 to 13 years old

	M6	M10	M14
**Score change in HFMSE score from baseline**			
Nx	33	19	5
Mean (SD)	2.8 (5.00)	2.6 (5.23)	2.6 (6.11)
Median	3	3	0
Q1, Q3	1, 6	-2, 6	-1, 3
Min, Max	-10, 14	-6, 15	-2, 13
**Score change in RULM from baseline**			
Nx	27	15	3
Mean (SD)	1.6 (2.53)	1.8 (2.24)	4.0 (1.73)
Median	1	2	3
Q1, Q3	0, 4	0, 4	3, 6
Min, Max	-2, 7	-2, 7	3, 6

Table 5c. Score change of motor measure from baseline to each visit for patients with SMA Type III and age more than 13 years old

	M6	M10	M14
**Score change in HFMSE score from baseline**			
Nx	20	17	5
Mean (SD)	4.5 (5.84)	4.6 (4.23)	5.2 (4.92)
Median	4	4	5
Q1, Q3	0, 8	1, 6	2, 9
Min, Max	-7, 15	0, 13	-1, 11
**Score change in RULM from baseline**			
Nx	17	14	5
Mean (SD)	1.9 (3.05)	0.2 (5.48)	-5.0 (6.67)
Median	1	0	-4
Q1, Q3	0, 3	-2, 4	-9, -1
Min, Max	-2, 10	-11, 9	-14, 3

Table 6a. Score change of motor measure from baseline to each visit for patients with SMA Type III and two SMN2 copy numbers

	M6	M10	M14
**Score change in HFMSE score from baseline**			
Nx	3	2	1
Mean (SD)	5.7 (7.64)	6.0 (7.07)	9.0 (-)
Median	4	6	9
Q1, Q3	-1, 14	1, 11	9, 9
Min, Max	-1, 14	1, 11	9, 9
**Score change in RULM from baseline**			
Nx	3	2	1
Mean (SD)	0.7 (1.15)	2.0 (2.83)	3.0 (-)
Median	0	2	3
Q1, Q3	0, 2	0, 4	3, 3
Min, Max	0, 2	0, 4	3, 3

Table 6b. Score change of motor measure from baseline to each visit for patients with SMA Type III and three SMN2 copy numbers

	M6	M10	M14
**Score change in HFMSE score from baseline**			
Nx	31	15	6
Mean (SD)	4.5 (6.35)	4.4 (6.38)	5.3 (6.09)
Median	3	2	5.5
Q1, Q3	1, 7	1, 7	-1, 11
Min, Max	-10, 18	-4, 18	-2, 13
**Score change in RULM from baseline**			
Nx	26	12	5
Mean (SD)	2.2 (3.80)	2.8 (3.96)	1.4 (1.82)
Median	1.5	2	2
Q1, Q3	0, 4	0, 4.5	0, 3
Min, Max	-3, 12	-2, 12	-1, 3

Table 6c Score change of motor measure from baseline to each visit for patients with SMA Type III and equal to or more than four SMN2 copy numbers

	M6	M10	M14
**Score change in HFMSE score from baseline**			
Nx	10	8	1
Mean (SD)	2.6 (3.20)	3.4 (2.77)	5.0 (-)
Median	2	3.5	5
Q1, Q3	0, 4	1, 5	5, 5
Min, Max	-1, 10	0, 8	5, 5
**Score change in RULM from baseline**			
Nx	8	6	1
Mean (SD)	1.5 (2.33)	1.2 (2.04)	3.0 (-)
Median	0	0	3
Q1, Q3	0, 3	0, 2	3, 3
Min, Max	0, 6	0, 5	3, 3

Table 7a. Response to treatment in terms of CHOP-INTEND by visits for patients with SMA Type I and age less than 7 months

	M6	M10	M14
**CHOP-INTEND, n(%)**			
Improving	1 (100.00%)	2 (100.00%)	1 (100.00%)
Stable	0 (0.00%)	0 (0.00%)	0 (0.00%)
Worsening	0 (0.00%)	0 (0.00%)	0 (0.00%)

Table 7b. Response to treatment in terms of CHOP-INTEND by visits for patients with SMA Type I and age more than 7 months

	M6	M10	M14
**CHOP-INTEND, n(%)**			
Improving	1 (20.00%)	2 (100.00%)	1 (50.00%)
Stable	4 (80.00%)	0 (0.00%)	1 (50.00%)
Worsening	0 (0.00%)	0 (0.00%)	0 (0.00%)

Table 8a. Response to treatment in terms of CHOP-INTEND by visits for patients with SMA Type I and two SMN2 copy numbers

	M6	M10	M14
**CHOP-INTEND, n(%)**			
Improving	1 (33.33%)	2 (100.00%)	1 (100.00%)
Stable	2 (66.67%)	0 (0.00%)	0 (0.00%)
Worsening	0 (0.00%)	0 (0.00%)	0 (0.00%)

Table 8b. Response to treatment in terms of CHOP-INTEND by visits for patients with SMA Type I and three SMN2 copy numbers

	M6	M10	M14
**CHOP-INTEND, n(%)**			
Improving	1 (50.00%)	2 (100.00%)	1 (100.00%)
Stable	1 (50.00%)	0 (0.00%)	0 (0.00%)
Worsening	0 (0.00%)	0 (0.00%)	0 (0.00%)

Table 9a. Response to treatment in terms of RULM and HFMSE by visits for patients with SMA Type II and age less than 5 years old

	M6	M10	M14
**RULM, n(%)**			
Improving	10 (62.50%)	6 (66.67%)	3 (50.00%)
Stable	6 (37.50%)	3 (33.33%)	3 (50.00%)
Worsening	0 (0.00%)	0 (0.00%)	0 (0.00%)
**HFMSE, n(%)**			
Improving	20 (80.00%)	14 (82.35%)	8 (88.89%)
Stable	4 (16.00%)	3 (17.65%)	1 (11.11%)
Worsening	1 (4.00%)	0 (0.00%)	0 (0.00%)

Table 9b. Response to treatment in terms of RULM and HFMSE by visits for patients with SMA Type II and age between 5 to 13 years old

	M6	M10	M14
**RULM, n(%)**			
Improving	21 (50.00%)	9 (40.91%)	3 (60.00%)
Stable	19 (45.24%)	13 (59.09%)	2 (40.00%)
Worsening	2 (4.76%)	0 (0.00%)	0 (0.00%)
**HFMSE, n(%)**			
Improving	23 (51.11%)	9 (34.62%)	2 (28.57%)
Stable	22 (48.89%)	17 (65.38%)	4 (57.14%)
Worsening	0 (0.00%)	0 (0.00%)	1 (14.29%)

Table 9c. Response to treatment in terms of RULM and HFMSE by visits for patients with SMA Type II and age more than 13 years old

	M6	M10
**RULM, n(%)**		
Improving	1 (33.33%)	0 (0.00%)
Stable	1 (33.33%)	0 (0.00%)
Worsening	1 (33.33%)	1 (100.00%)
**HFMSE, n(%)**		
Improving	2 (100.00%)	1 (100.00%)
Stable	0 (0.00%)	0 (0.00%)
Worsening	0 (0.00%)	0 (0.00%)

Table 10a. Response to treatment in terms of RULM and HFMSE by visits for patients with SMA Type II and two SMN2 copy numbers

	M6	M10
**RULM, n(%)**		
Improving	1 (33.33%)	0 (0.00%)
Stable	2 (66.67%)	2 (100.00%)
Worsening	0 (0.00%)	0 (0.00%)
**HFMSE, n(%)**		
Improving	1 (33.33%)	2 (66.67%)
Stable	2 (66.67%)	1 (33.33%)
Worsening	0 (0.00%)	0 (0.00%)

Table 10b. Response to treatment in terms of RULM and HFMSE by visits for patients with SMA Type II and three SMN2 copy numbers

	M6	M10	M14
**RULM, n(%)**			
Improving	23 (60.53%)	9 (52.94%)	3 (50.00%)
Stable	13 (34.21%)	7 (41.18%)	3 (50.00%)
Worsening	2 (5.26%)	1 (5.88%)	0 (0.00%)
**HFMSE, n(%)**			
Improving	35 (70.00%)	14 (51.85%)	5 (50.00%)
Stable	14 (28.00%)	13 (48.15%)	4 (40.00%)
Worsening	1 (2.00%)	0 (0.00%)	1 (10.00%)

Table 10c. Response to treatment in terms of RULM and HFMSE by visits for patients with SMA Type II and equal to or more than four SMN2 copy numbers

	M6	M10
**RULM, n(%)**		
Improving	1 (50.00%)	1 (100.00%)
Stable	1 (50.00%)	0 (0.00%)
Worsening	0 (0.00%)	0 (0.00%)
**HFMSE, n(%)**		
Improving	0 (0.00%)	1 (100.00%)
Stable	1 (100.00%)	0 (0.00%)
Worsening	0 (0.00%)	0 (0.00%)

Table 11a. Response to treatment in terms of RULM and HFMSE by visits for patients with SMA Type III and age less than 5 years old

	M6	M10	M14
**RULM, n(%)**			
Improving	4 (57.14%)	2 (50.00%)	1 (33.33%)
Stable	2 (28.57%)	2 (50.00%)	2 (66.67%)
Worsening	1 (14.29%)	0 (0.00%)	0 (0.00%)
**HFMSE, n(%)**			
Improving	6 (75.00%)	4 (66.67%)	3 (100.00%)
Stable	2 (25.00%)	2 (33.33%)	0 (0.00%)
Worsening	0 (0.00%)	0 (0.00%)	0 (0.00%)

Table 11b. Response to treatment in terms of RULM and HFMSE by visits for patients with SMA Type III and age between 5 to 13 years old

	M6	M10	M14
**RULM, n(%)**			
Improving	7 (25.93%)	4 (26.67%)	3 (100.00%)
Stable	20 (74.07%)	11 (73.33%)	0 (0.00%)
Worsening	0 (0.00%)	0 (0.00%)	0 (0.00%)
**HFMSE, n(%)**			
Improving	19 (57.58%)	11 (57.89%)	2 (40.00%)
Stable	10 (30.30%)	5 (26.32%)	3 (60.00%)
Worsening	4 (12.12%)	3 (15.79%)	0 (0.00%)

Table 11c. Response to treatment in terms of RULM and HFMSE by visits for patients with SMA Type III and age more than 13 years old

	M6	M10	M14
**RULM, n(%)**			
Improving	6 (35.29%)	5 (35.71%)	1 (20.00%)
Stable	11 (64.71%)	7 (50.00%)	1 (20.00%)
Worsening	0 (0.00%)	2 (14.29%)	3 (60.00%)
**HFMSE, n(%)**			
Improving	12 (60.00%)	9 (52.94%)	3 (60.00%)
Stable	7 (35.00%)	8 (47.06%)	2 (40.00%)
Worsening	1 (5.00%)	0 (0.00%)	0 (0.00%)

Table 12a. Response to treatment in terms of RULM and HFMSE by visits for patients with SMA Type III and two SMN2 copy numbers

	M6	M10	M14
**RULM, n(%)**			
Improving	0 (0.00%)	1 (50.00%)	1 (100.00%)
Stable	3 (100.00%)	1 (50.00%)	0 (0.00%)
Worsening	0 (0.00%)	0 (0.00%)	0 (0.00%)
**HFMSE, n(%)**			
Improving	2 (66.67%)	1 (50.00%)	1 (100.00%)
Stable	1 (33.33%)	1 (50.00%)	0 (0.00%)
Worsening	0 (0.00%)	0 (0.00%)	0 (0.00%)

Table 12b. Response to treatment in terms of RULM and HFMSE by visits for patients with SMA Type III and three SMN2 copy numbers

	M6	M10	M14
**RULM, n(%)**			
Improving	8 (30.77%)	3 (25.00%)	2 (40.00%)
Stable	17 (65.38%)	9 (75.00%)	3 (60.00%)
Worsening	1 (3.85%)	0 (0.00%)	0 (0.00%)
**HFMSE, n(%)**			
Improving	20 (64.52%)	7 (46.67%)	4 (66.67%)
Stable	8 (25.81%)	7 (46.67%)	2 (33.33%)
Worsening	3 (9.68%)	1 (6.67%)	0 (0.00%)

Table 12c. Response to treatment in terms of RULM and HFMSE by visits for patients with SMA Type III and equal to or more than four SMN2 copy numbers

	M6	M10	M14
**RULM, n(%)**			
Improving	2 (25.00%)	1 (16.67%)	1 (100.00%)
Stable	6 (75.00%)	5 (83.33%)	0 (0.00%)
Worsening	0 (0.00%)	0 (0.00%)	0 (0.00%)
**HFMSE, n(%)**			
Improving	5 (50.00%)	5 (62.50%)	1 (100.00%)
Stable	5 (50.00%)	3 (37.50%)	0 (0.00%)
Worsening	0 (0.00%)	0 (0.00%)	0 (0.00%)
